# Adaptation of the Mitsunobu Reaction for Facile Synthesis of Dorsomorphin-Based Library

**DOI:** 10.3390/molecules30112258

**Published:** 2025-05-22

**Authors:** Daria Novikova, Svetlana Vorona, Anastasiya Zenina, Tatyana Grigoreva, Vyacheslav Tribulovich

**Affiliations:** Laboratory of Molecular Pharmacology, St. Petersburg State Institute of Technology, St. Petersburg 190013, Russia; s.vorona@bk.ru (S.V.); asyazen@mail.ru (A.Z.); rozentatiana@gmail.com (T.G.)

**Keywords:** pyrazolo[1,5-a]pyrimidine, target-focused library, AMPK inhibitor, compound C, cross-coupling, Mitsunobu reaction

## Abstract

Pyrazolo[1,5-a]pyrimidine is a nitrogen-containing fused heterocycle that imitates the nitrogenous base adenine with varying degrees of reliability. This fact determines its frequent use in drug design, including the development of ATP-competitive kinase inhibitors. These include dorsomorphin which shows compromised kinase selectivity but is still widely used as an AMPK inhibitor. ATP-binding pockets of many proteins have a fairly conservative spatial structure and there is a high probability of obtaining a compound with low target selectivity during drug development. In the case of a common scaffold, the careful selection of side substituents that determine the activity and selectivity of the final compound plays an important role. In this work, a convergent strategy for the synthesis of dorsomorphin and its close analogs was developed and implemented. The resulting small series of compounds is distinguished by the maximum possible diversification and allows for an assessment of the biological activity towards AMPK. An original route to obtain variants of the phenoxy-alkylamine moiety of dorsomorphin via the Mitsunobu reaction will be useful for generating targeted-focused libraries of ATP-competitive kinase inhibitors and highly active receptor ligands.

## 1. Introduction

AMP-activated protein kinase (AMPK) has been a subject of intense research since its discovery in the 1980s. It plays a central role in the signaling pathway that triggers the metabolic switch from anabolic to catabolic processes, which is a response to energy stress [1]. AMPK is activated by an increase in the AMP/ATP ratio during hypoxia, starvation, glucose deficiency, or physical exercise and controls not only the distribution of energy in the cell, but also regulates the overall bioenergetics of the body, coordinating the reactions in tissues in accordance with the nutrition supply [2].

The key role of AMPK in regulating energy metabolism makes it an attractive therapeutic target for metabolic, inflammatory, and neurodegenerative diseases, and cancer [3]. It is particularly interesting that AMPK is activated by physiological regulators associated with health and longevity, such as calorie restriction [4] and physical activity [5], as well as by natural products used as traditional herbal medicines [6]. Many of these natural drugs, such as resveratrol [7] and berberine [8], are modified to enhance their activity and bioavailability.

Although the main efforts of pharmaceutical companies over the past decades have been directed at the search for and development of activators, recent studies of the AMPK role in cancer development have generated serious interest in its inhibitors [9]. In the context of cancer, AMPK plays a dual role, exhibiting protumor or antitumor effects [10]. In some cancers, AMPK activators may be beneficial for reducing the probability of disease occurrence in the risk group [11], but it is inhibitors that will be useful in the treatment of patients [12]. In this regard, there is a need for new selective AMPK inhibitors for both research purposes and therapeutic application.

Dorsomorphin discovered during high-throughput screening is considered to be the first selective AMPK inhibitor [13]. It was found that it binds to the catalytic site of AMPK, competing for binding with the ATP molecule, and thus inhibits the kinase activity of the enzyme [14]. Such an inhibition mechanism has its drawbacks since the ATP binding cavity is highly conserved [15], which can lead to the low selectivity of a potential inhibitor.

Since dorsomorphin was identified as the inhibitor by screening [16], no studies have been reported on the relationship of its structure and AMPK inhibitory activity. Here, we present the development of a method for the preparation of dorsomorphin based on an optimized synthesis strategy that allows minor variations in its structure. We identified a number of significant points for the modification of dorsomorphin that can be used for initial studies of the structure–activity relationship and implemented the proposed modifications using the developed synthetic scheme. The result of this work was the creation of a series of structural analogs of dorsomorphin that allow the study of the contribution of each structural dorsomorphin element to the inhibitory activity towards AMPK.

## 2. Results

**Figure 1:**
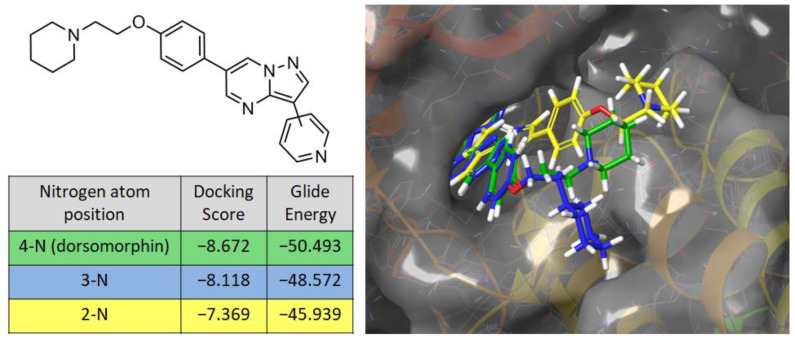
Binding energy parameters for dorsomorphin and two derivatives with different nitrogen atom positions in the pyridine core during docking at the ATP-binding site of AMPK.

The careful selection of protein-binding fragments of a small molecule and the way they are arranged in space is required to increase the selectivity of inhibitors. When studying the structure of dorsomorphin, we found that its position in the binding site does not allow for freely interpreting the pyrazolo[1,5-a]pyrimidine fragment of the molecule. Our computer modeling showed that an attempt to replace the 3-pyridyl substituent leads to a change in the binding mode compared with the X-ray data for dorsomorphin, while the calculated binding energy increases (Figure 1).

**Figure 2:**
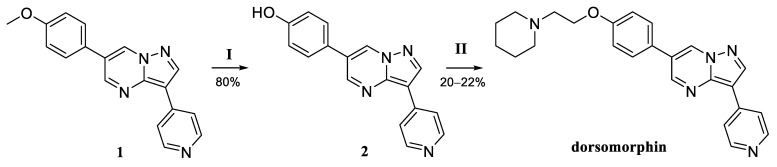
Scheme for obtaining dorsomorphin. Reagents and conditions: (**I**) BBr_3_, CH_2_Cl_2_, –78 °C, and 1 h; (**II**) 1-(2-chloroethyl) piperidine, K_2_CO_3_, DMF, 60 °C, and 16 h.

At the previous stage of the work, we synthesized a series of pyrazolo[1,5-a]pyrimidine derivatives, in which we implemented the variation of the 3-pyridyl substituent and the rotation of the phenyl fragment by introducing substituents into positions 5 and 7 of the pyrazolopyrimidine core [17]. It was assumed that these derivatives would be subjected to demethylation and alkylation with various halogen alkylamines to obtain the final compounds. In particular, to obtain exactly dorsomorphin, it is necessary to carry out the synthesis according to the scheme (Figure 2).

To obtain dorsomorphin, the first stage of demethylation was carried out in methylene chloride using BBr_3_ [18]. Then, the *O*-alkylation reaction of the released hydroxy group was carried out using 1-(2-chloroethyl) piperidine as a free base in dimethylformamide in the presence of K_2_CO_3_. The total yield after two stages did not exceed 15–18%, despite varying the reaction conditions. This failure forced us to reconsider the initial plans and develop another scheme for the synthesis of dorsomorphin and its analogs, making the most of the available groundwork.

**Figure 3:**
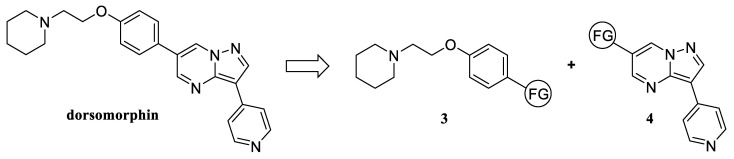
Retrosynthetic scheme for obtaining dorsomorphin.

Since we have previously obtained a series of substituted 6-bromopyrazolo[1,5-a]pyrimidines, we assumed that the synthesis of dorsomorphin could be realized based on a retrosynthetic scheme (Figure 3), where the final stage is the Suzuki–Miyaura cross-coupling between functionalized 1-(2-phenoxyethyl) piperidine **3** and pyrazolo[1,5-a]pyrimidine core 4. Speaking about the preparation of a series of compounds, the bulk of the modifications should be carried out with fragment **3**, which becomes responsible for the diversity of the library of dorsomorphin analogs with such a synthesis scheme.

**Figure 4:**
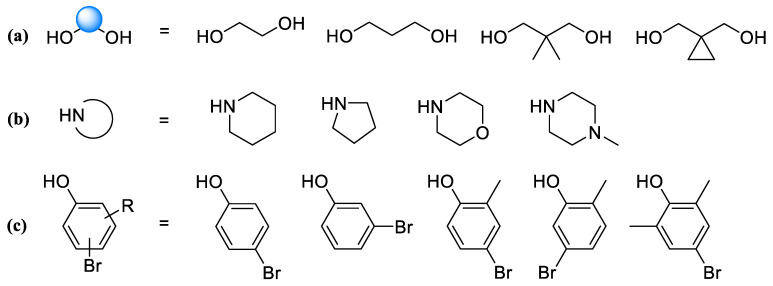
Reagents used to vary the structure of fragment **3**: glycols (**a**), cyclic amines (**b**), bromophenols (**c**).

When developing a series of dorsomorphin analogs, we primarily considered variants of fragment **3**, consisting in varying the alkyl chain, cycloamine fragment, and phenyl ring, for the implementation of which the following set of reagents was used during synthesis (Figure 4):

(a) Glycols—ethylene glycol, 1,3-propanediol, 2,2-dimethylpropane-1,3-diol, and 1,1-bis(hydroxymethyl)cyclopropane;
(b) Cyclic amines—piperidine, pyrrolidine, morpholine, and *N*-methylpiperazine;
(c) Bromophenols—4-bromophenol, 3-bromophenol, 4-bromo-2-methylphenol, 5-bromo-2-methylphenol, and 4-bromo-2,6-dimethylphenol.

**Figure 5:**
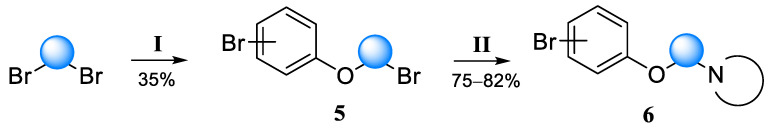
The most obvious scheme to synthesize **6** starting from dibromoethane. Reagents and conditions: (**I**) Br(CH_2_)_2_Br, K_2_CO_3_, MeCN, 60 °C, and 6 h; (**II**) R_2_NH, K_2_CO_3_, DMF, 60 °C, and 16 h.

The most obvious route to the synthesis of different variants of fragment **3**, where FG = Br, is shown in Figure 5. According to this scheme, the starting dibromoethane is first used for the *O*-alkylation of bromophenol to form the monophenyl-substituted product **5**, which is then *N*-alkylated with the corresponding secondary amine.

If we talk about the synthesis of fragment **5**, then in the literature one can find a large number of variants of the alkylation reaction of phenol with dibromoethane under a wide variety of conditions, with yields varying from 43% to 67%, while some authors claim a 95% yield of the product [19,20]. The main condition is the use of a 3–5-fold excess of dibromoethane to reduce the formation of diphenoxyethanes. We attempted to implement the scheme shown in Figure 5, but already at the first stage we were unable to obtain 1-bromo-4-(2-bromoethoxy)benzene with a yield of more than 35%. Considering the low yield and extremely high consumption of dibromoalkane, this route is of little use for introducing fragments based on glycols more complex than ethylene glycol or 1,3-propanediol into the final molecule.

**Figure 6:**
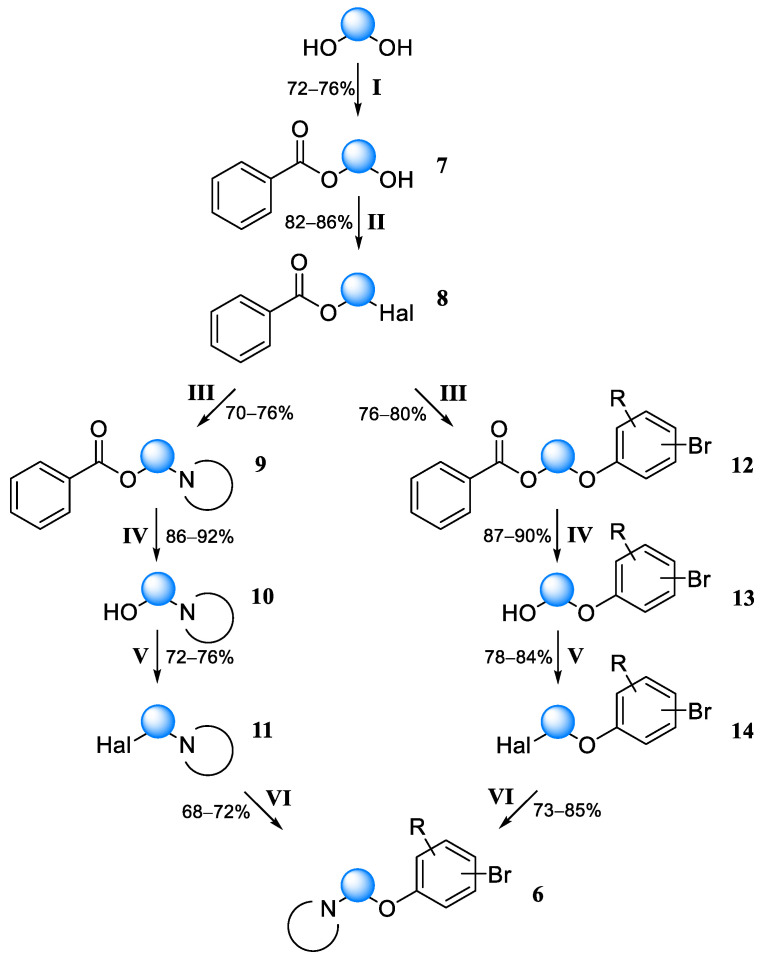
Synthetic scheme for obtaining variants of fragment **3** (compounds **6a**–**c**). Reagents and conditions: (**I**) BzCl, CH_2_Cl_2,_ N(Et)_3_, 5 °C, and 2 h then r.t., 12 h; (**II**) SOCl_2_, CH_2_Cl_2_, and 15 °C then r.t., 16 h or Br_2_, P(Ph)_3_, MeCN, 0 °C, and 30 min then reflux, 8 h; (**III**) R_2_NH or ArOH, K_2_CO_3_, DMF, 60 °C, and 16 h; (**IV**) NaOH, THF/H_2_O, 40 °C, and 12 h; (**V**) SOCl_2_, CH_2_Cl_2_, and 15 °C then r.t., 16 h or Br_2_, P(Ph)_3_, MeCN, 0 °C, and 30 min then reflux, 8 h; and (**VI**) R_2_NH or ArOH, K_2_CO_3_, DMF, 60 °C, and 16 h.

In this regard, we developed a more complex but universal scheme to synthesize analogs of fragment **3**, presented below (Figure 6, left path). We considered not only benzoic acid, but also acetic acid as an acyl component when implementing stage I. However, the monobenzoylation of glycols has two undoubted advantages:

− A high increment of the lipophilicity of the phenyl group [21], which is manifested in low solubility in water; this helps to get rid of excess water-soluble glycol at the stage of processing the reaction mixture;
− A sufficiently large difference in the boiling points of the original glycol and its mono- and dibenzoylated derivatives; this allows the isolation of the monobenzoylated product by vacuum distillation at a pressure of 0.5–1 Pa.

However, intermediates **9** obtained after the third stage of the synthesis (Figure 6) had, in most cases, too high of a boiling point for successful distillation. At the same time, we deliberately tried to avoid flash chromatography at the early stages of the synthesis to allow for potential scaling. From our point of view, monoacetylation should solve the problem of the high boiling point of intermediates. Acetylated glycols were distilled at a significantly lower temperature, but without the complete separation of mono- and diacetylated products. At the same time, the low lipophilicity increment of the methyl group made the monoacetyl derivative water-soluble, excluding the possibility of separating the excess of the starting glycol by aqueous washing. Thus, we opted for benzoic acid, which was subsequently used as an acyl component.

It is known that bromine is preferable to chlorine for nucleophilic substitution reactions (Figure 6, stage III). We prepared bromine derivatives by reacting a monoacyl derivative with bromine in acetonitrile in the presence of triphenylphosphine according to the procedure described in [22]. The nucleophilic substitution of halogen was carried out in dimethylformamide with K_2_CO_3_ as a base, and in the case of bromides the yields reached 84–92%, whereas in the case of chlorides the yields were noticeably lower. The hydrolysis of esters (Figure 6, stage IV) was carried out with sodium hydroxide in a water/THF mixture, with yields of 90–94%. The halogenation of amino alcohols at stage V of the synthesis was carried out similarly to stage II. The final stage was carried out according to the method used for stage III, but with slightly lower yields, while the use of pre-obtained phenolates did not lead to a significant increase in the reaction yield.

We also tested a synthesis scheme with an inverted order of introducing lateral substituents into glycols (Figure 6, right path). Differences in the paths were observed only at the last stage, since *O*-alkylation was replaced by *N*-alkylation. In the case of the reaction of a secondary amine with bromoalkoxybenzene, the yield of products reached 87%. Despite the available reports of almost quantitative yields when using chloroalkoxybenzenes [23], in our case the yields were significantly lower.

**Figure 7:**
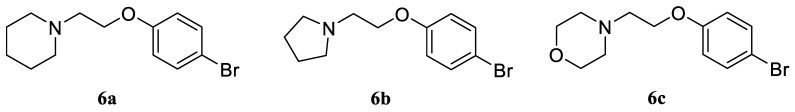
Variants of fragment **3** synthesized according to the scheme in Figure 6.

Three fragments were produced in preparative quantities according to the considered scheme (Figure 7). However, such a synthesis strategy is cumbersome and unsuitable for producing a larger number of variants for an expanded library of compounds.

**Figure 8:**
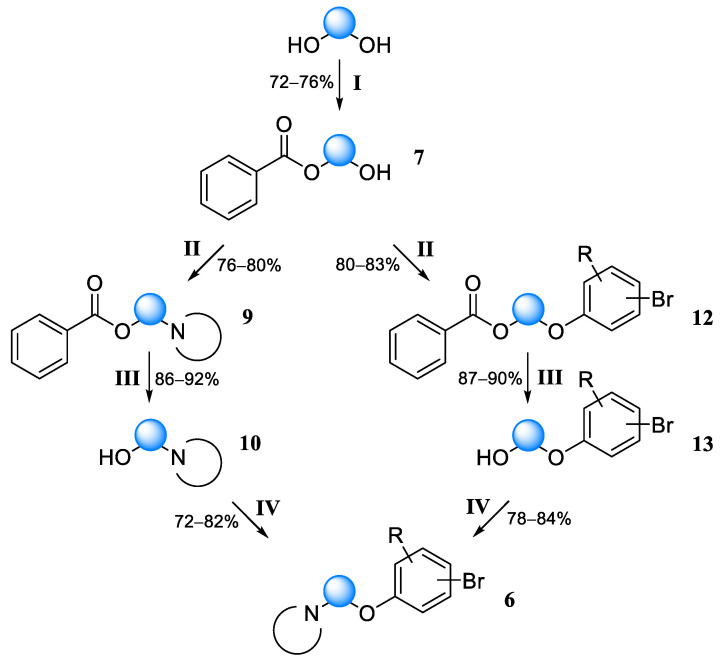
Synthetic scheme for obtaining variants of fragment **3** (compounds **6d**–**i**). Reagents and conditions: (**I**) BzCl, CH_2_Cl_2,_ N(Et)_3_, 5 °C, and 2 h then r.t., 12 h; (**II**) R_2_NH or ArOH, P(Ph)_3_, N(Et)_3_, DIAD, 4 °C, and 30 min then reflux 24 h; (**III**) NaOH, THF/H_2_O, 40 °C, and 12 h; and (**IV**) R_2_NH or ArOH, P(Ph)_3_, N(Et)_3_, DIAD, 4 °C, and 30 min then reflux 24 h.

To address this issue, we decided to eliminate the steps of halogenation (stages II and V in Figure 6) and perform direct dehydration using the Mitsunobu reaction. This reaction was discovered as a method for the formation of esters by inverting the configuration of alcohols [24]. The key reagent in this reaction is diisopropyl azodicarboxylate (DIAD). This method is quite applicable in various cases of dehydration, in particular, for the preparation of aryl-alkyl ethers [25] and formation of C–N bonds [26]. The synthesis scheme of fragment **3** and its analogs is significantly simplified using the Mitsunobu reaction (Figure 8).

Under the classical conditions of the Mitsunobu reaction (mixing the reagents at 0 °C, maintaining at room temperature), the yield was about 3% with a reaction time of 36 h, while HPLC analysis confirmed low conversion and the absence of by-products. An attempt to use ultrasound [27] did not lead to significant changes in the yield. The selection of conditions led to a reaction protocol of 24 h reflux in THF with triethylamine as a base catalyst [28], which gave good results even in the case of *N*-alkylation (yields 74–86%). Of course, there are other effective methods for the amination of alcohols [29], but in this case the Mitsunobu reaction has the advantages of synthesis simplicity and the availability of the reagents used.

**Figure 9:**
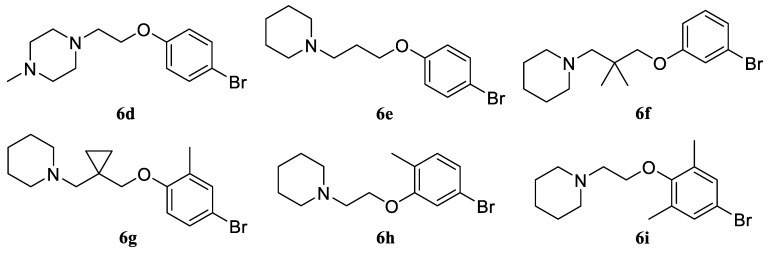
Variants of fragment **3** synthesized according to the scheme in Figure 8.

The purification of the products was straightforward and accomplished by passing through a layer of silica gel in a hexane/ethyl acetate system. The higher lipophilicity of the Mitsunobu reaction products compared with the starting materials allowed for obtaining compounds of satisfactory purity. Since the attack of the activated alcohol by the phenolate at the last stage of the Mitsunobu reaction occurs via the S_N_2 mechanism, synthesis involving sterically burdened ortho-substituted phenols often occurs in low yields [30]. In our case, the synthesis was successful not only with ortho-substituted phenols, but also with di-ortho-substituted phenols. Thus, using the developed scheme, six more variants of fragment **3** were obtained and characterized (Figure 9).

**Figure 10:**
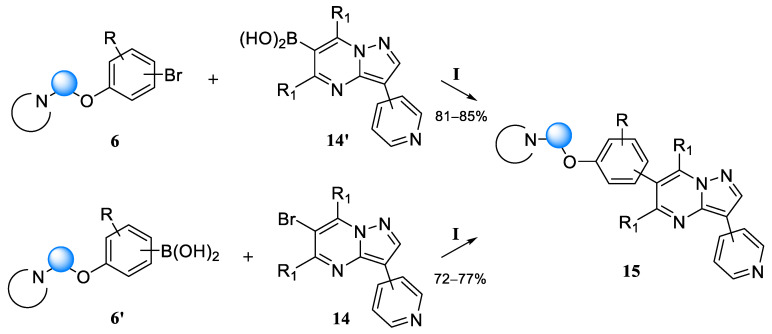
Options of final molecule assembly. Reagents and conditions: (**I**) Pd[P(Ph)_3_]_4_, K_2_CO_3_, THF/H_2_O, and reflux 4 h or Pd_2_(dba)_3_, Sphos, Cs_2_CO_3_, toluene, 100 °C, and 16 h.

The formation of the C–C bond between the synthesized fragments to obtain the final analogs of dorsomorphin was supposed to be carried out using the Suzuki–Miyaura method, which requires the functionalization of one of the combined fragments (Figure 10). From the point of view of constructing a library of compounds, it is more expedient to functionalize the building block, which varies to a lesser extent. In our case, a smaller number of modifications are implemented in the 6-bromo-3-pyridyl-substituted pyrazolo[1,5-a]pyrimidine fragment **14**.

**Figure 11:**
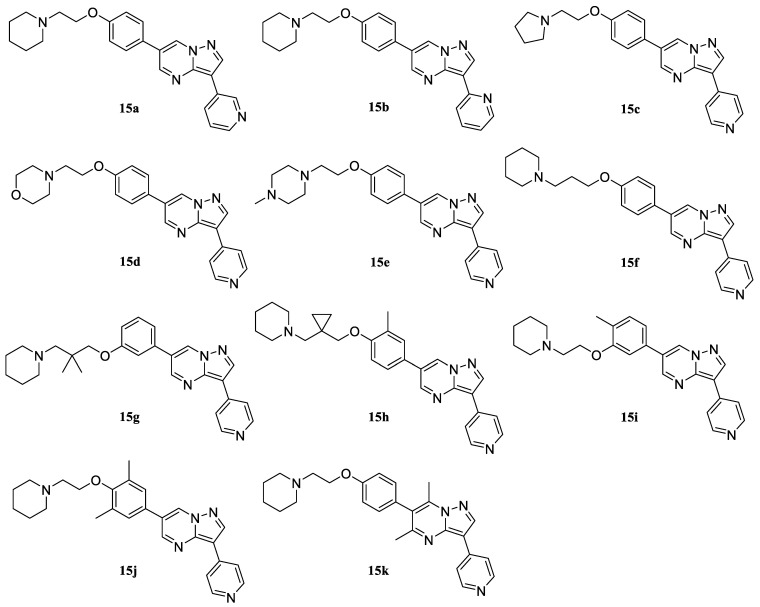
Series of dorsomorphin analogs.

The introduction of the boronic function into 6-bromo-3-pyridyl-substituted pyrazolo[1,5 a]pyrimidines was carried out by the interaction of trimethyl borate with lithium derivatives of pyrazolopyrimidines, which were obtained by the exchange reaction of bromine for lithium using n-butyl lithium in THF at −78 °C with yields of 57–62%. The formation of the C–C bond was carried out using the Suzuki–Miyaura reaction according to the method used earlier [17]. Thus, ten structures using various combinations of the combined fragments were synthesized according to this scheme (compounds **15a**–**j**, Figure 11).

In the case of the 5,7-dimethyl-substituted derivative, we failed to obtain boronic acid from the pyrazolo[1,5-a]pyrimidine part. Therefore, the synthesis of the final compound with 5,7-substitution in the core was carried out with the preliminary functionalization of 1-(2-(4-bromophenoxy)ethyl)piperidine **6a**, which can also be performed via the Grignard reagent [31]. The subsequent use of standard Pd[P(Ph)_3_]_4_ as a catalyst for the formation of the C–C bond did not lead to the production of detectable amounts of the target product. At the same time, the use of the Pd_2_dba_3_/S-Phos catalytic system proposed by S. Buchwald for sterically hindered cases of cross-coupling [17] showed satisfactory results and allowed us to obtain compound **15k** (Figure 11).

## 3. Discussion

The synthesis of targeted libraries of small molecules followed by testing the activity towards a specific target is critical for the identification of lead compounds in drug discovery. They also help to clarify the details of ligand–receptor interactions. Target-focused libraries are often based around a single core or scaffold with one or more (typically two or three) attachment points to which are appended specific substituents, or side chains, to arrive at the desired molecules [32]. The synthetic protocols presented in this paper yield a classical target-focused library of 480 compounds (Table S1) that is focused on ATP-binding enzymes but is not limited to kinases, since the pyrazolopyrimidine core is a recognized analog of adenosine [33]. The described approach can also be used to create sp^3^-enriched compounds based on the given pyrazolopyrimidines [34], as well as to study the structure–activity relationship by computer modeling.

Another feature of this work is the original route for obtaining fragment **3** variants, which can be useful not only for kinase-targeted libraries, but also for the synthesis of other biologically active compounds and drugs. The use of the proposed synthesis scheme involving the Mitsunobu reaction will allow for generating target-focused libraries based on various fragments of **6**, for example, to study binding to estrogen receptors [35] or to develop highly active ligands of histamine receptors [36].

Despite the fact that dorsomorphin was discovered quite a long time ago, its synthetic availability leaves much to be desired. Nevertheless, it remains one of the indispensable tools for studying the processes of AMPK inhibition [37], since no such potent inhibitor has been proposed yet. The synthesis presented in this article is an attempt to make available not only dorsomorphin, but also its closest structural analogs to researchers involved in the search and development of kinase inhibitors. The work conducted consisted not only in developing an optimal synthesis scheme, but also in verifying the methods and scaling up the reactions, which allows us to propose a method for the preparative production of compounds for more advanced stages of molecular design and the study of biological activity.

## 4. Materials and Methods

All starting compounds and reagents used are commercially available. The progress of reactions was monitored by TLC on silica gel 60 F254 plates (Merk, Rahway, NJ, USA) using n-hexane/ethyl acetate eluent. The purification and isolation of the products by flash chromatography was carried out using an Isolera Four Flash chromatograph on SNAP KP-Sil 100 g cartridges (Biotage, Uppsala, Sweden) with n-hexane/ethyl acetate eluent. HPLC analysis was performed on an LC-20 Prominence (Shimadzu, Kyoto, Japan) using a Nucleodur PolarTec column (Macherey-Nagel, Düren, Germany), with a length of 150 mm, internal diameter of 3.0 mm, and particle size of 3 µm, in acetonitrile–0.1% trifluoroacetic acid (50/50), and with a flow rate of 0.4 mL/min and oven temperature of 40 °C. Mass spectra were recorded on an LCMS-2020 device (Shimadzu) with a single quadrupole detector under positive mode, electrospray ionization (ESI).

**General procedure for demethylation.** BBr_3_ (1 M in dichloromethane, 28 mL, 28 mmol) was added at −50 °C dropwise to a suspension of ether (4.6 mmol) in dichloromethane (20 mL). The reaction mixture was stirred at room temperature overnight. The mixture was concentrated to dryness, cooled on an ice bath and diluted with saturated aqueous NaHCO_3_. The resulting precipitate was filtered and air-dried, the crude product was used without further purification.

*4-(3-(pyridin-4-yl)pyrazolo[1,5-a]pyrimidin-6-yl)phenol* (**2**). Yellowish solid. ^1^H NMR (400 MHz, DMSO-*d*_6_) δ 9.86 (s, 1H), 9.60 (d, *J* = 2.3 Hz, 1H), 9.23 (d, *J* = 2.3 Hz, 1H), 9.22 (s, 1H), 8.82 (d, *J* = 6.8 Hz, 2H), 8.66 (d, *J* = 6.8 Hz, 2H), 7.74 (d, *J* = 8.7 Hz, 2H), and 6.94 (d, *J* = 8.7 Hz, 2H). ^13^C NMR (101 MHz, DMSO) δ 158.36, 152.53, 145.68, 144.82, 142.38, 142.33, 133.18, 128.47, 123.95, 123.13, 120.49, 116.14, and 104.36. MS (ESI) *m*/*z:* 289.1 (100) [M + H]^+^.

**General procedure for nucleophilic substitution.** Halide (0.05 mol), *O*- or *N*-nucleophile (0.055 mol), and finely ground K_2_CO_3_ were dissolved in 200 mL of anhydrous DMF and stirred at 60 °C for 16 h. The solution was filtered from an inorganic precipitate, and DMF was distilled off under reduced pressure. The residue was poured into 200 mL of water, extracted with 3 × 100 mL of ethyl acetate, washed twice with water and saturated NaCl solution, and dried over Na_2_SO_4_. The product was isolated by flash chromatography using hexane/ethyl acetate (6:1–9:1) as eluent.

*6-(4-(2-(Piperidin-1-yl)ethoxy)phenyl)-3-(pyridin-4-yl)pyrazolo[1,5-a]pyrimidine* (dorsomorphin). Yield 18% (two stages), yellowish solid. ^1^H NMR (400 MHz, DMSO-*d*_6_) δ 9.52 (d, *J* = 2.3 Hz, 1H), 9.12 (d, *J* = 2.3 Hz, 1H), 8.97 (s, 1H), 8.59 (d, *J* = 6.2 Hz, 2H), 8.16 (d, *J* = 6.2 Hz, 2H), 7.82 (d, *J* = 8.8 Hz, 2H), 7.11 (d, *J* = 8.8 Hz, 2H), 4.14 (t, *J* = 5.9 Hz, 2H), 2.68 (t, *J* = 5.9 Hz, 2H), 2.47–2.40 (m, 4H), 1.54–1.47 (m, 4H), and 1.41–1.36 (m, 2H). ^13^C NMR (101 MHz, DMSO) δ 158.97, 150.80, 149.99, 143.85, 143.81, 139.29, 132.61, 128.27, 125.30, 122.18, 119.63, 115.29, 106.08, 65.81, 57.35, 54.44, 25.61, and 23.97. MS (ESI) *m*/*z:* 400.2 (100) [M + H]^+^.

*2-(Piperidin-1-yl)ethyl benzoate* (**9a**). Yield 73%, colorless liquid. ^1^H NMR (400 MHz, DMSO-*d*_6_) δ 7.97–7.93 (m, 2H), 7.65 (t, *J* = 7.4 Hz, 1H), 7.52 (t, *J* = 7.7 Hz, 2H), 4.35 (t, *J* = 5.9 Hz, 2H), 2.63 (t, *J* = 5.9 Hz, 2H), 2.45–2.37 (m, 4H), 1.50–1.43 (m, 4H), and 1.38–1.31 (m, 2H). ^13^C NMR (101 MHz, DMSO) δ 165.65, 133.26, 129.83, 129.10, 128.74, 62.40, 56.79, 54.17, 25.63, and 23.89.

*2-(4-Bromophenoxy)ethyl benzoate* (**12a**). Yield 77%, colorless liquid. ^1^H NMR (400 MHz, DMSO-*d*_6_) δ 7.97–7.93 (m, 2H), 7.66 (tt, *J* = 7.0, 1.3 Hz, 1H), 7.52 (t, *J* = 7.7 Hz, 2H), 7.45 (d, *J* = 9.0 Hz, 2H), 6.97 (d, *J* = 9.1 Hz, 2H), 4.61–4.58 (m, 2H), and 4.35–4.32 (m, 2H). ^13^C NMR (101 MHz, DMSO) δ 165.66, 157.60, 133.46, 132.18, 129.45, 129.21, 128.77, 116.96, 112.30, 66.14, and 63.28.

**General procedure for obtaining glycol monobenzoates.** To a solution of diole (0.4 mol) in 300 mL CH_2_Cl_2_ containing 55.6 mL triethylamine (40.5 g, 0.4 mol) and cooled to 0 °C, a solution of 28 g benzoyl chloride (0.2 mol) in 100 mL of CH_2_Cl_2_ was added dropwise over 2 h, maintaining the temperature no higher than 5 °C. After complete addition, the solution was stirred at room temperature for 12 h. The resulting solution was washed with water 2 × 200 mL, then with 200 mL of 5% hydrochloric acid, and again with water 2 × 200 mL, 5% NaHCO_3_ solution, and saturated NaCl solution, and dried over Na_2_SO_4_. The solvent was removed under reduced pressure. The residue was distilled at a pressure of 5 mbar.

*2-Hydroxyethyl benzoate* (**7a**). 74%, colorless liquid. ^1^H NMR (400 MHz, DMSO-*d*_6_) δ 8.02–7.99 (m, 2H), 7.64 (tt, *J* = 6.9, 1.3 Hz, 1H), 7.52 (t, *J* = 7.7 Hz, 1H), 4.90 (br s, 1H), 4.31–4.27 (m, 2H), and 3.74–3.70 (m, 2H). ^13^C NMR (101 MHz, DMSO) δ 165.91, 133.27, 129.92, 129.28, 128.69, 66.60, and 59.14.

**General procedure for obtaining chlorides.** To a solution of carbinol (0.1 mol) in 300 mL dichloromethane cooled to 4 °C, 14.3 mL thionyl chloride (23.4 g, 0.2 mol) was added dropwise with stirring at such a rate that the temperature of the mixture did not exceed 15 °C. After the addition was complete, the mixture was left at room temperature for 16 h. The solvent was distilled to dryness under reduced pressure, 100 mL of 10% NaHCO_3_ solution and 150 mL of ethyl acetate were added to the residue, and the mixture was stirred for 20 min. The layers were separated, and the aqueous layer was extracted with ethyl acetate 2 × 150 mL. The organic layer was dried over Na_2_SO_4_ and distilled under reduced pressure. The residue was distilled at a pressure of 5–10 mbar.

*2-Chloroethyl benzoate* (**8a-Cl**). Yield 85%, colorless liquid. ^1^H NMR (400 MHz, DMSO-*d*_6_) δ 8.01–7.97 (m, 2H), 7.70–7.65 (m, 1H), 7.57–7.52 (m, 2H), 4.56–4.52 (m, 2H), and 3.99–3.95 (m, 2H). ^13^C NMR (101 MHz, DMSO) δ 165.44, 133.57, 129.31, 129.23, 128.83, 64.63, and 42.69.

*1-(2-Chloroethyl)piperidine* (**11a-Cl**). Yield 76%, colorless liquid. ^1^H NMR (400 MHz, DMSO-*d*_6_) δ 3.64 (t, *J* = 6.9 Hz, 2H), 2.58 (t, *J* = 6.9 Hz, 2H), 2.44–2.33 (m, 4H), 1.51–1.43 (m, 4H), and 1.40–1.33 (m, 2H). ^13^C NMR (101 MHz, DMSO) δ 60.01, 53.87, 41.60, 25.51, and 23.90.

**General procedure for obtaining bromides.** To a solution of 13.1 g triphenylphosphine (0.05 mol) in 200 mL anhydrous acetonitrile, 2.6 mL bromine (8 g, 0.05 mol) and 0.05 mol of carbinol were added with stirring at 0 °C. The mixture was stirred at room temperature for 30 min, then refluxed for 8 h. After the reaction was complete, the solvent was distilled to dryness under reduced pressure, and 300 mL of a hexane/ethyl acetate mixture (6:1) was added to the residue and stirred for an hour. The solution was filtered from the precipitated triphenylphosphine oxide, washed with 10% NaHCO_3_ solution and twice with water and saturated NaCl solution. The organic layer was dried over Na_2_SO_4_ and passed through a 10 cm layer of silica gel, which was then washed with 500 mL of the same mixture. After the distillation of the solvent under reduced pressure, a crude bromine derivative suitable for further reactions was obtained.

*2-Bromoethyl benzoate* (**8a-Br**). Yield 84%, colorless liquid. ^1^H NMR (400 MHz, DMSO-*d*_6_) δ 8.02–7.99 (m, 2H), 7.61–7.56 (m, 1H), 7.49–7.44 (m, 2H), 4.51–4.48 (m, 2H), and 3.68–3.65 (m, 2H). ^13^C NMR (100 MHz, DMSO) δ 166.45, 133.06, 130.46, 129.75, 128.85, 65.27, and 29.18.

*1-(2-Bromoethyl)piperidine* (**11a-Br**). Yield 73%, colorless liquid. ^1^H NMR (400 MHz, DMSO-*d*_6_) δ 3.53 (t, *J* = 7.1 Hz, 2H), 3.00 (t, *J* = 7.1 Hz, 2H), 2.48–2.37 (m, 4H), 1.54–1.46 (m, 4H), and 1.42–1.35 (m, 2H). ^13^C NMR (101 MHz, DMSO) δ 59.41, 53.79, 29.24, 25.55, and 23.90.

*1-Bromo-4-(2-bromoethoxy)benzene* (**14a-Br**). Yield 82%, colorless liquid. ^1^H NMR (400 MHz, DMSO-*d*_6_) δ 7.45 (d, *J* = 9.1 Hz, 2H), 6.94 (d, *J* = 9.1 Hz, 2H), 4.32–4.29 (m, 2H), and 3.81–3.77 (m, 2H). ^13^C NMR (101 MHz, DMSO) δ 157.20, 132.21, 116.94, 112.45, 67.98, and 31.25.

**General procedure for alkaline hydrolysis.** Benzoate (0.05 mol) was dissolved in 150 mL THF with stirring, and a solution of 8 g sodium hydroxide (0.2 mol) in 100 mL water was added. The resulting mixture was kept for 12 h at 40 °C with vigorous stirring. Upon the completion of the reaction, the aqueous layer was separated, and the organic layer was evaporated to dryness under reduced pressure. The residue was dissolved in 200 mL ethyl acetate, filtered, and passed through a 10 cm layer of silica gel, which was additionally washed with 400 mL ethyl acetate. The solvent was distilled off under reduced pressure. If necessary, the resulting product was distilled at 5–10 mbar.

*2-(Piperidin-1-yl)ethan-1-ol* (**10a**). Yield 92%, colorless liquid. ^1^H NMR (400 MHz, DMSO-*d*_6_) δ 4.32 (t, *J* = 5.2 Hz, 1H), 3.46 (td, *J* = 6.4, 5.1 Hz, 2H), 2.36–2.30 (m, 4H), 2.31 (t, *J* = 6.5 Hz, 2H), 1.50–1.43 (m, 4H), and 1.38–1.32 (m, 1H). ^13^C NMR (101 MHz, DMSO) δ 61.12, 58.58, 54.55, 25.62, and 24.09.

*2-(4-Bromophenoxy)ethan-1-ol* (**13a**). Yield 87%, colorless liquid. ^1^H NMR (400 MHz, DMSO-*d*_6_) δ 7.43 (d, *J* = 9.0 Hz, 2H), 6.91 (d, *J* = 9.0 Hz, 2H), 4.89 (t, *J* = 5.5 Hz, 1H), 3.96 (t, *J* = 4.9 Hz, 2H), and 3.70 (td, *J* = 5.4, 5.0 Hz, 2H). ^13^C NMR (101 MHz, DMSO) δ 158.01, 132.11, 116.76, 111.81, 69.82, and 59.48.

Using the above protocols, the following fragments were obtained according to the scheme presented in Figure 6:

*1-(2-(4-Bromophenoxy)ethyl)piperidine* (**6a**). Yield 33% (six stages), colorless liquid. ^1^H NMR (400 MHz, DMSO-*d*_6_) δ 7.42 (d, *J* = 9.1 Hz, 2H), 6.90 (d, *J* = 9.1 Hz, 2H), 4.03 (t, *J* = 5.9 Hz, 2H), 2.62 (t, *J* = 5.9 Hz, 2H), 2.43–2.36 (m, 4H), 1.51–1.44 (m, 4H), and 1.39–1.32 (m, 2H). ^13^C NMR (101 MHz, DMSO) δ 157.79, 132.05, 116.77, 111.81, 65.89, 57.24, 54.38, 25.57, and 23.92. MS (ESI) *m*/*z*: 284.1 (100) [M + H]^+^.

*1-(2-(4-Bromophenoxy)ethyl)pyrrolidine* (**6b**). Yield 31% (six stages), colorless liquid. ^1^H NMR (400 MHz, DMSO-*d*_6_) δ 7.38 (d, *J* = 9.0 Hz, 2H), 6.86 (d, *J* = 9.0 Hz, 2H), 4.03 (t, *J* = 5.9 Hz, 2H), 2.73 (t, *J* = 5.9 Hz, 2H), 2.45–2.38 (m, 4H), and 1.69–1.60 (m, 4H). ^13^C NMR (101 MHz, DMSO) δ 157.80, 132.01, 116.75, 111.81, 64.76, 56.93, 53.82, and 23.07. MS (ESI) *m*/*z*: 270.0 (100) [M + H]^+^.

*4-(2-(4-Bromophenoxy)ethyl)morpholine* (**6c**). Yield 33% (six stages), colorless liquid. ^1^H NMR (400 MHz, DMSO-*d*_6_) δ 7.35 (d, *J* = 9.0 Hz, 2H), 6.83 (d, *J* = 9.0 Hz, 2H), 4.06 (t, *J* = 5.9 Hz, 2H), 3.57–2.53 (m, 4H), 2.68 (t, *J* = 5.9 Hz, 2H), and 2.39–2.34 (m, 4H). ^13^C NMR (101 MHz, DMSO) δ 157.82, 132.03, 116.76, 111.80, 66.21, 65.55, 57.02, and 53.66. MS (ESI) *m*/*z*: 286.0 (100) [M + H]^+^.

**General procedure for obtaining compounds by the Mitsunobu reaction.** Substituted phenol or secondary amine (0.05 mol), alcohol component (0.055 mol), triphenylphosphine (14.4 g, 0.055 mol), and triethylamine (5 g, 0.05 mol) were dissolved in 100 mL THF with stirring. The mixture was cooled and a solution of DIAD (11.1 g, 0.055 mol) in 30 mL of THF was added dropwise at 4 °C over 30 min. After complete addition, the cooling was removed and the mixture was stirred for another 30 min, then refluxed for 24 h with stirring. The solvent was distilled off under reduced pressure. The residue was diluted with 400 mL hexane/ethyl acetate (6:1) mixture and stirred for an hour. The solution was filtered from the precipitated triphenylphosphine oxide, washed with 10% NaHCO_3_ solution, twice with water, and with saturated NaCl, and dried over Na_2_SO_4_. The solvent was distilled off under reduced pressure. The residue was dissolved in a hexane/ethyl acetate mixture (9:1–19:1) and passed through a 10 cm layer of silica gel, which was then washed with 500 mL of the same mixture. After distilling off the solvent, a viscous oil representing a product with a purity of about 95% was obtained.

*1-(2-(4-Bromophenoxy)ethyl)-4-methylpiperazine* (**6d**). Yield 46% (four stages), colorless liquid. ^1^H NMR (400 MHz, DMSO-*d*_6_) δ 7.40 (d, *J* = 9.1 Hz, 2H), 6.85 (d, *J* = 9.1 Hz, 2H), 4.03 (t, *J* = 5.8 Hz, 2H), 2.65 (t, *J* = 5.8 Hz, 2H), 2.43–2.33 (m, 4H), 2.32–2.22 (m, 4H), and 2.12 (s, 3H). ^13^C NMR (101 MHz, DMSO) δ 157.80, 132.11, 116.74, 111.85, 65.00, 57.22, 54.74, 53.21, and 45.83. MS (ESI) *m*/*z*: 299.1 (100) [M + H]^+^.

*1-(3-(4-Bromophenoxy)propyl)piperidine* (**6e**). Yield 49% (four stages), colorless liquid. ^1^H NMR (400 MHz, DMSO-*d*_6_) δ 7.42 (d, *J* = 9.0 Hz, 2H), 6.91 (d, *J* = 9.0 Hz, 2H), 4.09 (t, *J* = 6.2, 2H), 2.61 (t, *J* = 6.2 Hz, 2H), 2.46–2.36 (m, 4H), 1.86 (p, *J* = 6.2 Hz, 2H), 1.55–1.48 (m, 4H), and 1.39–1.33 (m, 2H). ^13^C NMR (101 MHz, DMSO) δ 157.78, 132.12, 116.75, 111.86, 66.72, 62.20, 54.37, 27.88, 25.58, and 23.91. MS (ESI) *m*/*z*: 298.1 (100) [M + H]^+^.

*1-(3-(3-Bromophenoxy)-2,2-dimethylpropyl)piperidine* (**6f**). Yield 42% (four stages), colorless liquid. ^1^H NMR (400 MHz, DMSO-*d*_6_) δ 7.22 (t, *J* = 8.1 Hz, 1H), 7.13–7.08 (m, 2H), 6.93 (ddd, *J* = 8.3, 2.4, 0.9 Hz, 1H), 3.88 (s, 2H), 3.78 (s, 2H), 2.43–2.36 (m, 4H), 1.50–1.44 (m, 4H), 1.39–1.31 (m, 2H), and 0.91 (s, 6H). ^13^C NMR (101 MHz, DMSO) δ 160.15, 131.12, 123.16, 122.02, 117.29, 113.97, 69.33, 64.59, 54.38, 36.35, 25.57, 23.93, and 21.49. MS (ESI) *m*/*z*: 326.1 (100) [M + H]^+^.

*1-((1-((4-Bromo-2-methylphenoxy)methyl)cyclopropyl)methyl)piperidine* (**6g**). Yield 40% (four stages), colorless liquid. ^1^H NMR (400 MHz, DMSO-*d*_6_) δ 7.31–7.22 (m, 2H), 6.87 (d, *J* = 8.6 Hz, 1H), 3.85 (s, 2H), 3.63 (s, 2H), 2.47–2.32 (m, 4H), 2.11 (s, 3H), 1.53–1.44 (m, 4H), 1.44–1.31 (m, 2H), and 0.79–0.71 (m, 4H). ^13^C NMR (101 MHz, DMSO) δ 156.30, 132.52, 131.54, 129.32, 113.47, 111.31, 68.66, 65.03, 54.38, 25.99, 24.46, 22.46, 15.62, and 7.91. MS (ESI) *m*/*z*: 338.1 (100) [M + H]^+^.

*1-(2-(5-Bromo-2-methylphenoxy)ethyl)piperidine* (**6h**). Yield 41% (four stages), colorless liquid. ^1^H NMR (400 MHz, DMSO-*d*_6_) δ 7.10–7.06 (m, 2H), 7.03–6.98 (m, 1H), 4.05 (t, *J* = 5.8 Hz, 2H), 2.66 (t, *J* = 5.8 Hz, 2H), 2.45–2.33 (m, 4H), 2.10 (s, 3H), 1.55–1.43 (m, 4H), and 1.40–1.34 (m, 2H). ^13^C NMR (101 MHz, DMSO) δ 157.46, 131.75, 125.50, 122.81, 118.99, 114.49, 66.12, 57.38, 54.39, 25.58, 23.92, and 15.60. MS (ESI) *m*/*z*: 298.1 (100) [M + H]^+^.

*1-(2-(4-Bromo-2,6-dimethylphenoxy)ethyl)piperidine* (**6i**). Yield 39% (four stages), colorless liquid. ^1^H NMR (400 MHz, DMSO-*d*_6_) δ 7.85 (d, *J* = 2.3 Hz, 2H), 3.97 (t, *J* = 5.8 Hz, 2H), 2.64 (t, *J* = 5.8 Hz, 2H), 2.44–2.37 (m, 4H), 2.22 (s, 6H), 1.51–1.44 (m, 4H), and 1.39–1.33 (m, 2H). ^13^C NMR (101 MHz, DMSO) δ 156.13, 132.99, 120.02, 113.42, 66.01, 57.38, 54.40, 25.55, 23.90, and 15.38. MS (ESI) *m*/*z*: 312.1 (100) [M + H]^+^.

**General procedure for obtaining aryl-, heteroarylboronic acids.** Aryl bromide (10 mmol) and 40 mL THF were placed in a dried flask purged with argon. The resulting solution was cooled to −78 °C, and 10 mL of n-BuLi 1 M solution in hexane was added dropwise and stirred for 2 h at −78 °C. Then, trimethyl borate (0.98 mL, 11 mmol) in 10 mL THF was added and stirred for 1 h at −78 °C. After warming to room temperature, the reaction mixture was diluted with 20 mL water, and 10 mL of saturated NH_4_Cl solution was added and stirred for 1 h. Then, the solvent was distilled to dryness under reduced pressure, and the residue was extracted with ethyl acetate. The resulting solution was dried with Na_2_SO_4_ and completely evaporated under reduced pressure. The residue was dissolved in 10 mL ethyl acetate and the product was precipitated by adding hexane. The resulting oil was suitable for cross-coupling according to Suzuki–Miyaura. If necessary, the product was purified by recrystallization from a hexane-ethyl acetate mixture without identification.

**General procedure for obtaining 6-aryl-substituted 3-pyridylpyrazolo[1,5-a]pyrimidines.** Substituted aryl bromide (10 mmol), 0.05 g Pd[P(Ph)_3_]_4_ (0.043 mmol, 0.3 mol%) and 3.1 g (3-pyridylpyrazolo[1,5-a]pyrimidin-6-yl)boronic acid (13 mmol) were dissolved in 100 mL THF with stirring. The resulting mixture was stirred for 30 min under a weak stream of argon, and then a solution of 5 g K_2_CO_3_ (36 mmol) in 50 mL water was added. The reaction mixture was refluxed for 4 h with vigorous stirring in a weak stream of argon. Upon the completion of the reaction, the solvent was distilled to dryness under reduced pressure. The residue was stirred in 200 mL of boiling butyl acetate, then filtered hot. The filtrate was clarified with 2 g silica gel while boiling and stirring, filtered again, and the volume of the solution was brought to 30 mL. The precipitate formed after 16 h was filtered.

**General procedure for obtaining 6-aryl-5,7-dimethyl-substituted 3-pyridylpyrazolo[1,5-a]pyrimidines.** A total of 0.04 g Pd_2_(dba)_3_ (0.044 mmol, 0.66 mol%) and 0.08 g 2-dicyclohexylphosphino-2′,6′-dimethoxybiphenyl (Sphos, 0.195 mmol) were dissolved in 120 mL toluene with stirring. The solution was stirred for 30 min under a slight stream of argon. 6-Bromo-5,7-dimethyl-substituted 3-pyridylpyrazolo[1,5-a]pyrimidine (2 g, 6.6 mmol), boronic acid (11 mmol), and 6 g Cs_2_CO_3_ (18.4 mmol) were added to the reaction mixture. The reaction was carried out at 100 °C with vigorous stirring in a weak stream of argon for 16 h. After the reaction was complete, the volume of toluene was brought to 200 mL, the mixture was heated until boiling and filtered hot. The filtrate was clarified with 1 g silica gel while boiling and stirring, filtered again, and the volume of the solution was brought to 15 mL. The precipitated product was filtered after 24 h.

*6-(4-(2-(Piperidin-1-yl)ethoxy)phenyl)-3-(pyridin-3-yl)pyrazolo[1,5-a]pyrimidine* (**15a**). Yield 49% (from two stages), yellowish solid. ^1^H NMR (400 MHz, DMSO-*d*_6_) δ 9.50 (d, *J* = 2.3 Hz, 1H), 9.39 (dd, *J* = 2.3, 0.8 Hz, 1H), 9.08 (d, *J* = 2.3 Hz, 1H), 8.89 (s, 1H), 8.54 (ddd, *J* = 8.0, 2.3, 1.6 Hz, 1H), 8.46 (dd, *J* = 4.8, 1.6 Hz, 1H), 7.82 (d, *J* = 8.8 Hz, 2H), 7.49 (ddd, *J* = 8.0, 4.8, 0.9 Hz, 1H), 7.11 (d, *J* = 8.8 Hz, 2H), 4.15 (t, *J* = 5.8 Hz, 2H), 2.69 (t, *J* = 5.8 Hz, 2H), 2.50–2.45 (m, 4H), 1.55–1.47 (m, 4H), and 1.43–1.35 (m, 2H). ^13^C NMR (101 MHz, DMSO) δ 159.75, 150.42, 146.88, 146.66, 143.28, 142.92, 132.39, 132.30, 128.20, 128.01, 125.47, 123.77, 121.80, 115.26, 106.43, 65.80, 57.34, 54.44, 25.60, and 23.96. MS (ESI) *m*/*z:* 400.2 (100) [M + H]^+^.

*6-(4-(2-(Piperidin-1-yl)ethoxy)phenyl)-3-(pyridin-2-yl)pyrazolo[1,5-a]pyrimidine* (**15b**). Yield 48% (from two stages), yellowish solid. ^1^H NMR (400 MHz, DMSO-*d*_6_) δ 9.49 (d, *J* = 2.2 Hz, 1H), 9.09 (d, *J* = 2.2 Hz, 1H), 8.81 (s, 1H), 8.60 (ddd, *J* = 4.9, 1.8, 1.0, 1H), 8.47 (dt, *J* = 7.7, 1.1 Hz, 1H), 7.88 (td, *J* = 7.7, 1.8 Hz, 1H), 7.82 (d, *J* = 8.7 Hz, 2H), 7.24 (ddd, *J* = 7.6, 4.8, 1.2 Hz, 1 H), 7.10 (d, *J* = 8.7 Hz, 2H), 4.13 (t, *J* = 5.6 Hz, 2H), 2.67 (t, *J* = 5.6 Hz, 2H), 2.49–2.39 (m, 4H), 1.55–1.46 (m, 4H), and 1.42–1.34 (m, 2H). ^13^C NMR (101 MHz, DMSO) δ 158.90, 150.93, 150.44, 149.39, 144.06, 143.55, 136.75, 132.54, 128.22, 125.42, 121.80, 121.11, 120.31, 115.26, 109.65, 65.79, 57.34, 54.43, 25.60, and 23.95. MS (ESI) *m*/*z:* 400.2 (100) [M + H]^+^.

*3-(Pyridin-4-yl)-6-(4-(2-(pyrrolidin-1-yl)ethoxy)phenyl)pyrazolo[1,5-a]pyrimidine* (**15c**). Yield 50% (from two stages), yellowish solid. ^1^H NMR (400 MHz, DMSO-*d*_6_) δ 9.54 (d, *J* = 2.3 Hz, 1H), 9.14 (d, *J* = 2.3 Hz, 1H), 8.99 (s, 1H), 8.60 (d, *J* = 5.3 Hz, 2H), 8.15 (d, *J* = 5.3 Hz, 2H), 7.85 (d, *J* = 8.9 Hz, 2H), 7.12 (d, *J* = 8.9 Hz, 2H), 4.14 (t, *J* = 5.7 Hz, 2H), 2.79 (t, *J* = 5.7 Hz, 2H), 2.46–2.39 (m, 4H), and 1.68–1.60 (m, 4H). ^13^C NMR (101 MHz, DMSO) δ 158.98, 150.75, 149.98, 143.86, 143.82, 139.31, 132.60, 128.28, 125.35, 122.20, 119.65, 115.30, 106.07, 64.67, 57.03, 53.88, and 23.11. MS (ESI) *m*/*z*: 386.2 (100) [M + H]^+^.

*4-(2-(4-(3-(Pyridin-4-yl)pyrazolo[1,5-a]pyrimidin-6-yl)phenoxy)ethyl)morpholine* (**15d**). Yield 46% (from two stages), yellowish solid. ^1^H NMR (400 MHz, DMSO-*d*_6_) δ 9.53 (d, *J* = 2.3 Hz, 1H), 9.13 (d, *J* = 2.3 Hz, 1H), 8.97 (s, 1H), 8.59 (d, *J* = 5.2 Hz, 2H), 8.16 (d, *J* = 5.2 Hz, 2H), 7.83 (d, *J* = 8.8 Hz, 2H), 7.11 (d, *J* = 8.8 Hz, 2H), 4.17 (t, *J* = 5.8 Hz, 2H), 3.56–3.52 (m, 4H), 2.74 (t, *J* = 5.8 Hz, 2H), and 2.40–2.35 (m, 4H). ^13^C NMR (101 MHz, DMSO) δ 158.82, 150.60, 149.99, 143.84, 143.80, 139.27, 132.58, 128.27, 125.31, 122.17, 119.63, 115.28, 106.71, 66.27, 65.50, 57.12, and 53.81. MS (ESI) *m*/*z*: 402.2 (100) [M + H]^+^.

*6-(4-(2-(4-Methylpiperazin-1-yl)ethoxy)phenyl)-3-(pyridin-4-yl)pyrazolo[1,5-a]pyrimidine* (**15e**). Yield 50% (from two stages), yellowish solid. ^1^H NMR (400 MHz, DMSO-*d*_6_) δ 9.54 (d, *J* = 2.2 Hz, 1H), 9.14 (d, *J* = 2.2 Hz, 1H), 8.98 (s, 1H), 8.60 (d, *J* = 5.2 Hz, 2H), 8.18 (d, *J* = 5.2 Hz, 2H), 7.85 (d, *J* = 8.8 Hz, 2H), 7.12 (d, *J* = 8.8 Hz, 2H), 4.17 (t, *J* = 5.8 Hz, 2H), 2.69 (t, *J* = 5.8 Hz, 2H), 2.49–2.39 (m, 4H), 2.38–2.28 (m, 4H), and 2.12 (s, 3H). ^13^C NMR (101 MHz, DMSO) δ 158.90, 150.70, 149.98, 143.84, 143.79, 139.25, 132.55, 128.26, 125.29, 122.16, 119.65, 115.29, 106.53, 64.91, 57.32, 54.80, 53.25, and 45.83. MS (ESI) *m*/*z*: 415.2 (100) [M + H]^+^.

*6-(4-(3-(Piperidin-1-yl)propoxy)phenyl)-3-(pyridin-4-yl)pyrazolo[1,5-a]pyrimidine* (**15f**). Yield 48% (from two stages), yellowish solid. ^1^H NMR (400 MHz, DMSO-*d*_6_) δ 9.53 (d, *J* = 2.2 Hz, 1H), 9.14 (d, *J* = 2.2 Hz, 1H), 8.97 (s, 1H), 8.59 (d, *J* = 5.2 Hz, 2H), 8.16 (d, *J* = 5.2 Hz, 2H), 7.83 (d, *J* = 8.9 Hz, 2H), 7.11 (d, *J* = 8.9 Hz, 2H), 4.13 (t, *J* = 5.9, 2H), 2.72 (t, *J* = 5.9 Hz, 2H), 2.45–2.35 (m, 4H), 1.89 (*p*, *J* = 5.9 Hz, 2H), 1.54–1.47 (m, 4H), and 1.39–1.32 (m, 2H). ^13^C NMR (101 MHz, DMSO) δ 158.98, 150.77, 149.99, 143.85, 143.81, 139.29, 132.61, 128.27, 125.30, 122.18, 119.62, 115.23, 106.03, 66.67, 62.30, 54.45, 27.88, 25.60, and 23.98. MS (ESI) *m*/*z*: 414.2 (100) [M + H]^+^.

*6-(3-(2,2-Dimethyl-3-(piperidin-1-yl)propoxy)phenyl)-3-(pyridin-4-yl)pyrazolo[1,5-a]pyrimidine* (**15g**). Yield 49% (from two stages), yellowish solid. ^1^H NMR (400 MHz, DMSO-*d*_6_) δ 9.62 (d, *J* = 2.3 Hz, 1H), 9.16 (d, *J* = 2.3 Hz, 1H), 8.99 (s, 1H), 8.59 (d, *J* = 6.2 Hz, 2H), 8.16 (d, *J* = 6.2 Hz, 2H), 7.48–7.44 (m, 3H), 7.07–7.00 (m, 1H), 3.90 (s, 2H), 3.76 (s, 2H), 2.46–2.36 (m, 4H), 1.53–1.41 (m, 4H), 1.41–1.32 (m, 2H), and 0.91 (s, 6H). ^13^C NMR (101 MHz, DMSO) δ 159.95, 150.93, 149.99, 144.14, 144.12, 139.20, 134.54, 133.66, 130.34, 122.15, 119.66, 119.17, 114.35, 112.35, 106.19, 68.81, 63.75, 54.44, 36.39, 25.62, 23.98, and 21.48. MS (ESI) *m*/*z*: 442.3 (100) [M + H]^+^.

*6-(3-Methyl-4-((1-(piperidin-1-ylmethyl)cyclopropyl)methoxy)phenyl)-3-(pyridin-4-yl)pyrazolo[1,5-a]pyrimidine* (**15h**). Yield 45% (from two stages), yellowish solid. ^1^H NMR (400 MHz, DMSO-*d*_6_) δ 9.59 (d, *J* = 2.3 Hz, 1H), 9.12 (d, *J* = 2.3 Hz, 1H), 8.98 (s, 1H), 8.61 (d, *J* = 5.2 Hz, 2H), 8.15 (d, *J* = 5.2 Hz, 2H), 7.63–7.55 (m, 2H), 7.07 (d, *J* = 8.6 Hz, 1H), 3.89 (s, 2H), 3.61 (s, 2H), 2.48–2.35 (m, 4H), 2.11 (s, 3H), 1.52–1.45 (m, 4H), 1.44–1.35 (m, 2H), and 0.75–0.68 (m, 4H). ^13^C NMR (101 MHz, DMSO) δ 158.12, 150.78, 149.99, 143.86, 143.84, 139.29, 133.15, 132.62, 131.40, 128.77, 122.18, 120.45, 119.65, 116.50, 106.84, 67.99, 64.23, 54.50, 25.63, 23.90, 22.40, 15.60, and 7.92. MS (ESI) *m*/*z*: 454.3 (100) [M + H]^+^.

*6-(4-Methyl-3-(2-(piperidin-1-yl)ethoxy)phenyl)-3-(pyridin-4-yl)pyrazolo[1,5-a]pyrimidine* (**15i**). Yield 47% (from two stages), yellowish solid. ^1^H NMR (400 MHz, DMSO-*d*_6_) δ 9.53 (d, *J* = 2.3 Hz, 1H), 9.13 (d, *J* = 2.3 Hz, 1H), 8.98 (s, 1H), 8.60 (d, *J* = 6.2 Hz, 2H), 8.17 (d, *J* = 6.2 Hz, 2H), 7.53–7.49 (m, 1H), 7.14–7.09 (m, 2H), 4.17 (t, *J* = 5.9 Hz, 2H), 2.70 (t, *J* = 5.9 Hz, 2H), 2.45–2.31 (m, 4H), 2.10 (s, 3H), 1.55–1.46 (m, 4H), and 1.41–1.32 (m, 2H). ^13^C NMR (101 MHz, DMSO) δ 158.56, 150.77, 149.96, 143.86, 143.80, 139.29, 132.57, 131.77, 125.60, 124.27, 122.75, 122.18, 119.63, 115.45, 106.29, 66.10, 57.35, 54.42, 25.60, 23.95, and 15.60. MS (ESI) *m*/*z*: 414.2 (100) [M + H]^+^.

*6-(3,5-Dimethyl-4-(2-(piperidin-1-yl)ethoxy)phenyl)-3-(pyridin-4-yl)pyrazolo[1,5-a]pyrimidine* (**15j**). Yield 50% (from two stages), yellowish solid. ^1^H NMR (400 MHz, DMSO-*d*_6_) δ 9.50 (d, *J* = 2.2 Hz, 1H), 9.11 (d, *J* = 2.2 Hz, 1H), 8.96 (s, 1H), 8.58 (d, *J* = 5.2 Hz, 2H), 8.14 (d, *J* = 5.2 Hz, 2H), 7.49 (d, *J* = 2.4 Hz, 2H), 4.08 (t, *J* = 5.8 Hz, 2H), 2.70 (t, *J* = 5.8 Hz, 2H), 2.48–2.40 (m, 4H), 2.22 (s, 6H), 1.52–1.45 (m, 4H), and 1.40–1.35 (m, 2H). ^13^C NMR (101 MHz, DMSO) δ 156.22, 150.75, 149.97, 143.86, 143.82, 139.30, 132.94, 129.80, 127.78, 125.30, 122.18, 119.60, 106.15, 65.98, 57.42, 54.44, 25.61, 23.97, and 15.38. MS (ESI) *m*/*z:* 428.2 (100) [M + H]^+^.

*5,7-Dimethyl-6-(4-(2-(piperidin-1-yl)ethoxy)phenyl)-3-(pyridin-4-yl)pyrazolo[1,5-a]pyrimidine* (**15k**). Yield 35% (from two stages), yellowish solid. ^1^H NMR (400 MHz, DMSO-*d*_6_) δ 8.91 (s, 1H), 8.56 (d, *J* = 6.2 Hz, 2H), 8.19 (d, *J* = 6.2 Hz, 2H), 7.32 (d, *J* = 8.7 Hz, 2H), 7.11 (d, *J* = 8.7 Hz, 2H), 4.14 (t, *J* = 5.6 Hz, 2H), 2.68 (t, *J* = 5.6 Hz, 2H), 2.48 (s, 3H), 2.48–2.38 (m, 4H), 2.35 (s, 3H), 1.53–1.44 (m, 4H), and 1.40–1.33 (m, 2H). ^13^C NMR (101 MHz, DMSO) δ 159.63, 159.23, 149.85, 144.14, 143.84, 142.82, 139.81, 131.25, 126.90, 122.23, 119.42, 114.82, 105.89, 65.81, 57.34, 54.44, 25.60, 24.87, 23.96, and 14.89. MS (ESI) *m*/*z*: 428.2 (100) [M + H]^+^.

## 5. Conclusions

Thus, in the course of the work conducted, a convergent strategy for the synthesis of dorsomorphin was implemented; based on this strategy, 11 structural analogs were first synthesized for further study of the structure–activity relationship. Eight structures were first obtained and described. The synthesized small series of compounds is distinguished by the maximum possible diversification within the target-focused library of compounds, which allows for an evaluative determination of biological activity towards AMPK to select a further direction of research.

## Data Availability

The synthesized intermediate products (**6a**–**i**) and final compounds (**15a**–**k**), as well as the target-focused library of 480 compounds as a *.sdf file, are available upon request.

## Supplementary Materials

The following supporting information can be downloaded at https://www.mdpi.com/article/10.3390/molecules30112258/s1: Table S1: Annotation of the structure identifier (XXXXX) for compounds that make up the target-focused library of dorsomorphin analogs; Figures S1–S42: ^1^H and ^13^C NMR spectra of compounds.

## Abbreviations

The following abbreviations are used in this manuscript:

AMPK	AMP-activated protein kinase
THF	Tetrahydrofuran
DIAD	Diisopropyl azodicarboxylate
TLC	Thin-layer chromatography
DMF	Dimethylformamide
