# Supplementary material for: Adaptation of the Mitsunobu Reaction for Facile Synthesis of Dorsomorphin-Based Library
Source: Molecules. 2025 May 22;30(11):2258. doi: 10.3390/molecules30112258 (PMC12156883; doi:10.3390/molecules30112258)

Table S1. Annotation of the structure identifier (XXXXX) for compounds that make up the target-focused library of dorsomorphin analogs.

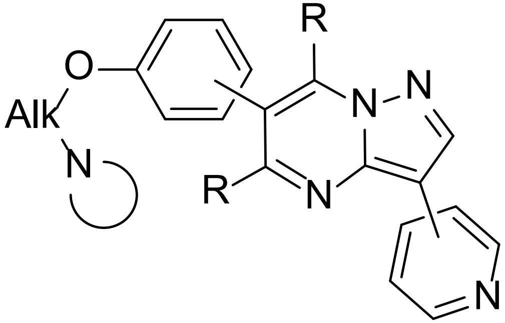

Identifier position	Point of structure variation	Variation is realized by	Variants	Designation	Structure
First number	Nitrogen atom position in the pyridine ring	Corresponding pyridinecarboxylic acids:<br>- 4-pyridinecarboxylic acid (isonicotinic acid)<br>- 3-pyridinecarboxylic acid (nicotinic acid)<br>- 2-pyridinecarboxylic acid (picolinic acid)	Nitrogen atom at position 4	4XXXX	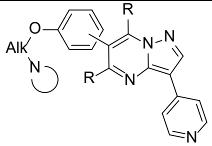
			Nitrogen atom at position 3	3XXXX	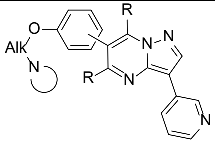
			Nitrogen atom at position 2	2XXXX	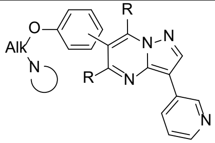
Second number	Substitution in the pyrimidine ring	Corresponding β-dicarbonyl compounds:<br>- bromomalonic aldehyde<br>- 3-bromoacetylacetone	No substitution at positions 5 and 7 (R = H)	X0XXX	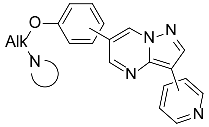
			Dimethyl substitution at positions 5 and 7 (R = CH_3)	X2XXX	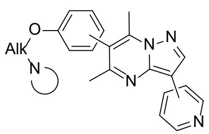

Third number	Orientation of alkylamine moiety and substitution in the phenyl ring	Corresponding bromophenols:<br>- 4-bromophenol<br>- 3-bromophenol<br>- 4-bromo-2-methylphenol<br>- 5-bromo-2-methylphenol<br>- 4-bromo-2,6-dimethylphenol	Para-orientation of the chain, no substitution	XX1XX	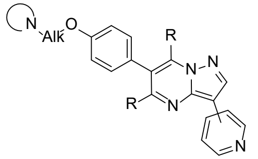
			Meta-orientation of the chain, no substitution	XX2XX	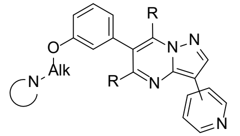
			Para-orientation of the chain, methyl substitution	XX3XX	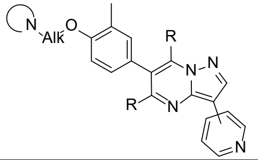
			Meta-orientation of the chain, methyl substitution	XX4XX	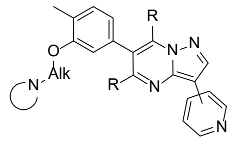
			Para-orientation of the chain, dimethyl substitution	XX5XX	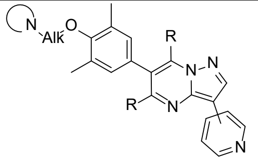
Fourth number	Length and substitution in the alkyl chain	Corresponding glycols:<br>- ethylene glycol<br>- 1,3-propanediol<br>- 2,2-dimethylpropane-1,3-diol<br>- 1,1-bis(hydroxymethyl)cyclopropane	Two-carbon chain, no substitution	XXX1X	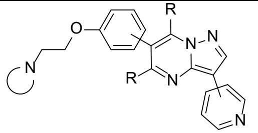
			Three-carbon chain, no substitution	XXX2X	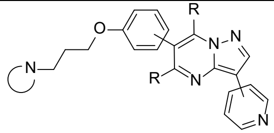
			Three-carbon chain, dimethyl substitution	XXX3X	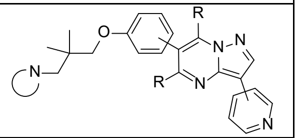
			Three-carbon chain, cyclopropyl substitution	XXX4X	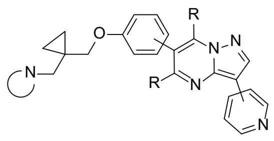

Fifth number	Cyclic amine moiety	Corresponding cyclic amines:<br>- piperidine<br>- pyrrolidine<br>- morpholine<br>- *N*-methylpiperazine	*N*-piperidinyl moiety	XXXX1	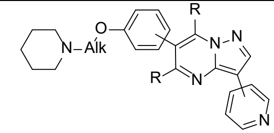
			N-pyrrolidinyl moiety	XXXX2	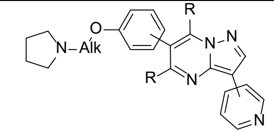
			N-morpholinyl moiety	XXXX3	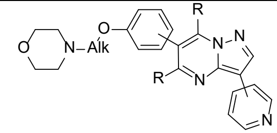
			N-(4-methylpiperazinyl) moiety	XXXX4	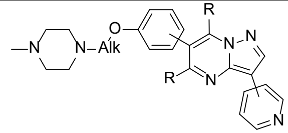

Note. Dorsomorphin is also included in the library and designated as 40111.

Figure S1. ^{1}H NMR spectrum of **dorsomorphin**.

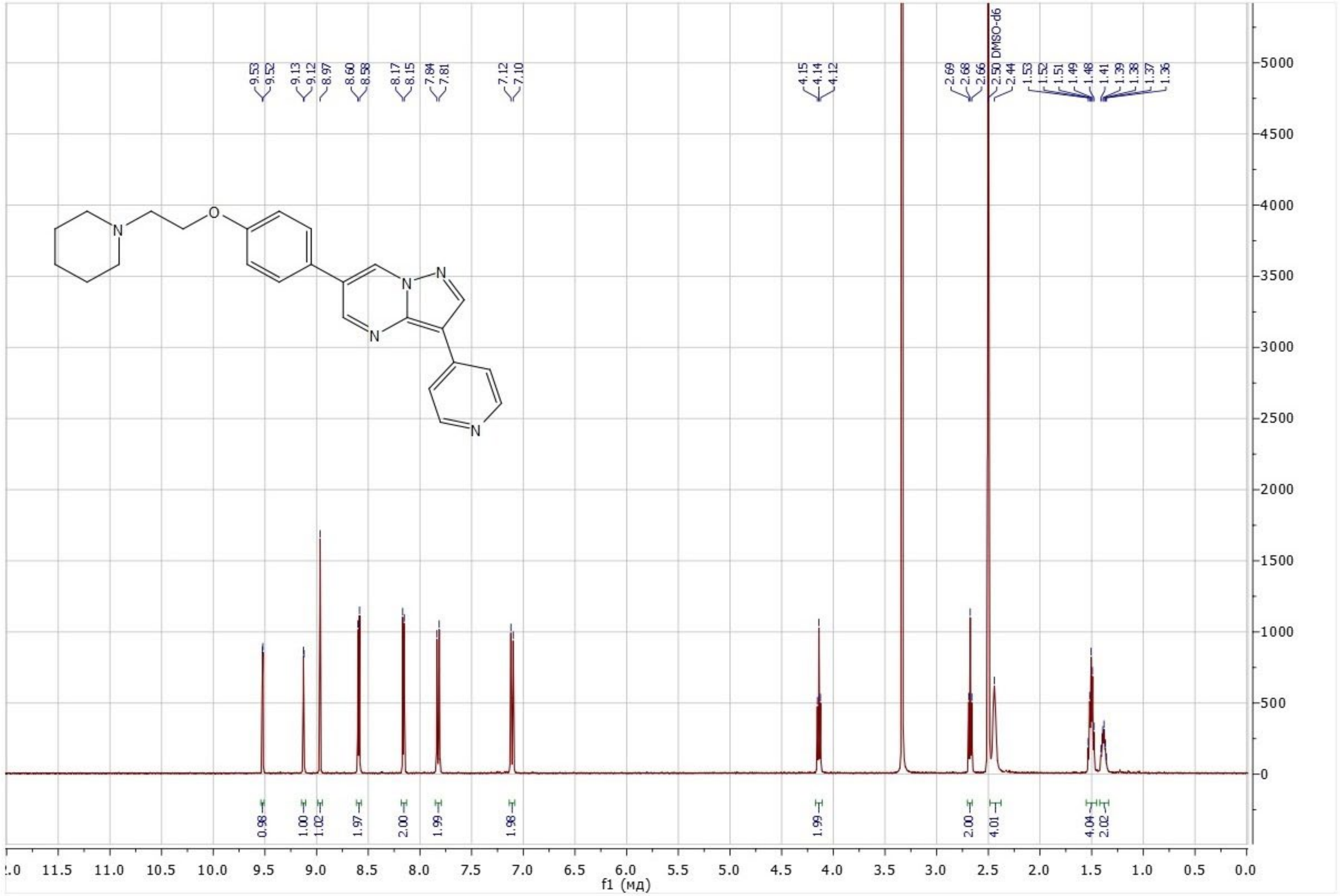

Figure S2. ^{13}C NMR spectrum of **dorsomorphin**.

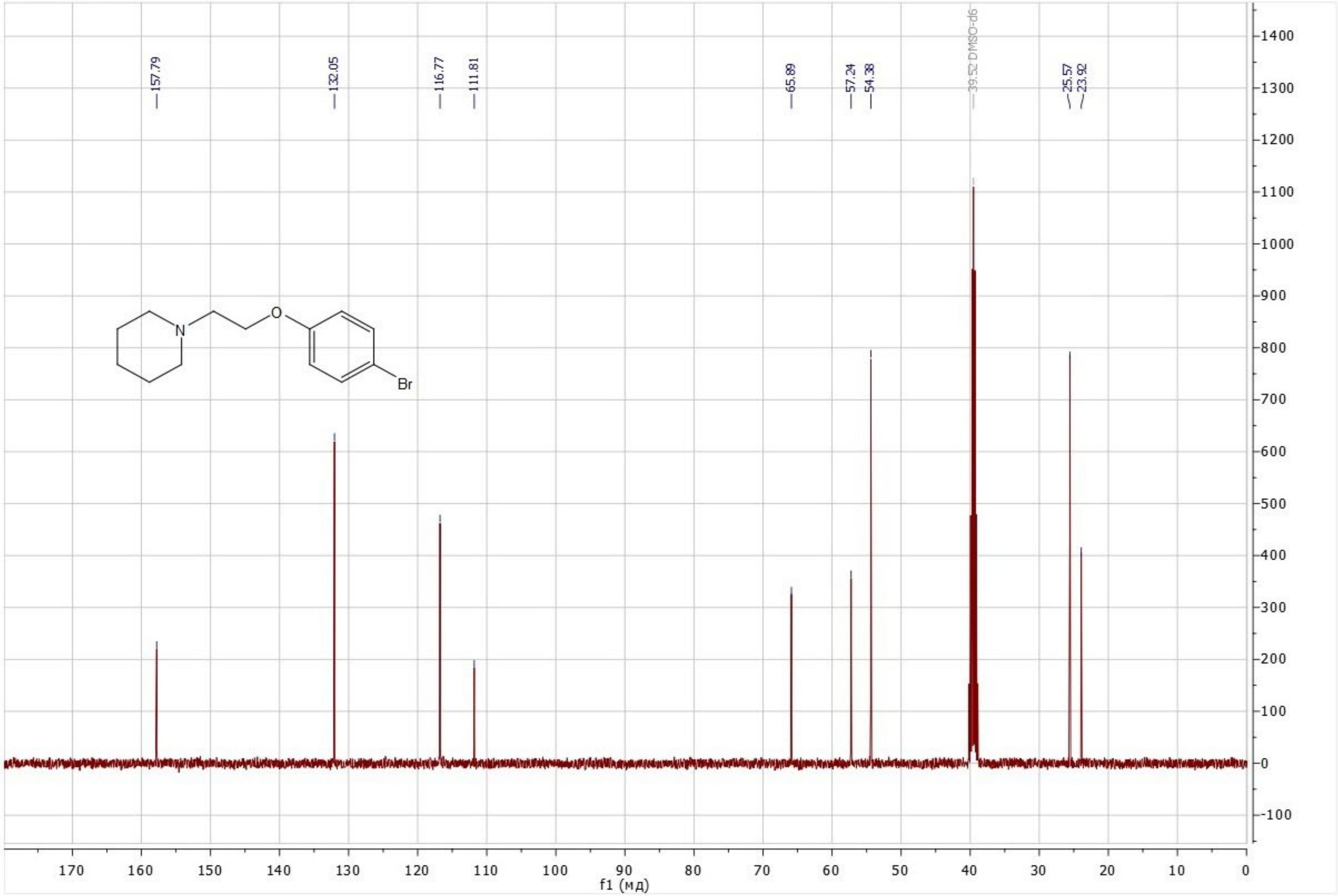

Figure S3. ^{1}H NMR spectrum of **6a**.

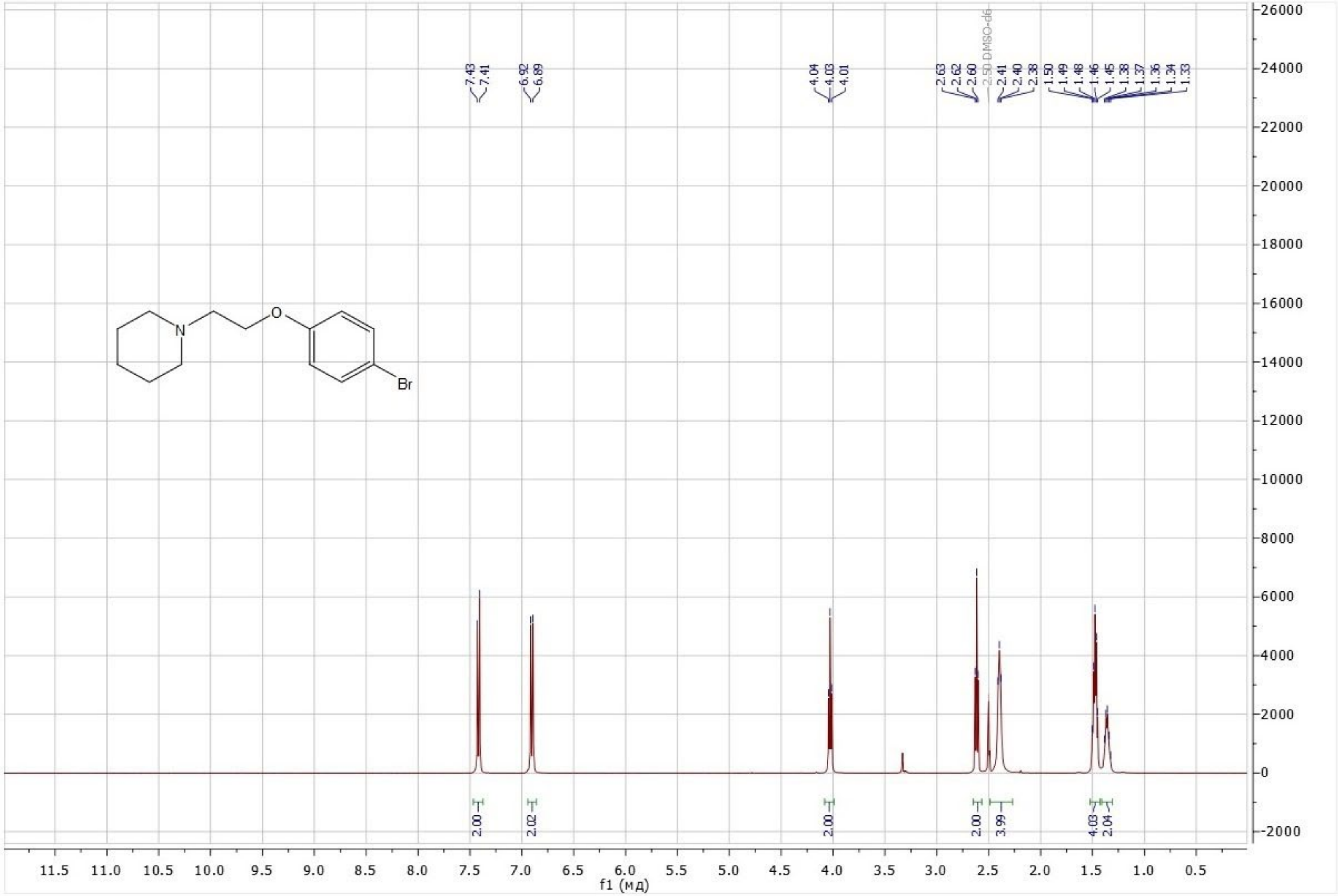

Figure S4. ^{13}C NMR spectrum of **6a**.

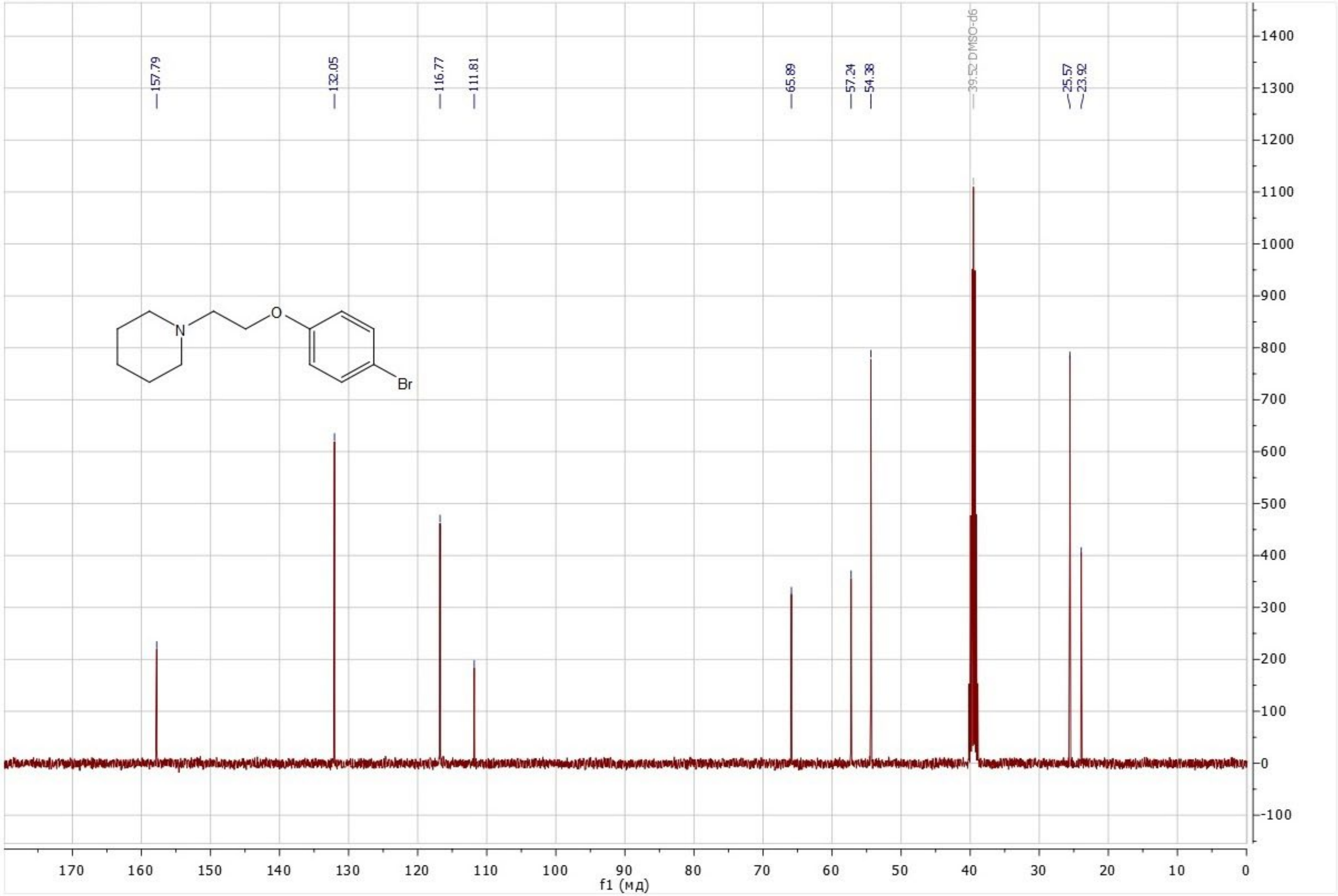

Figure S5. ^{1}H NMR spectrum of **6b**.

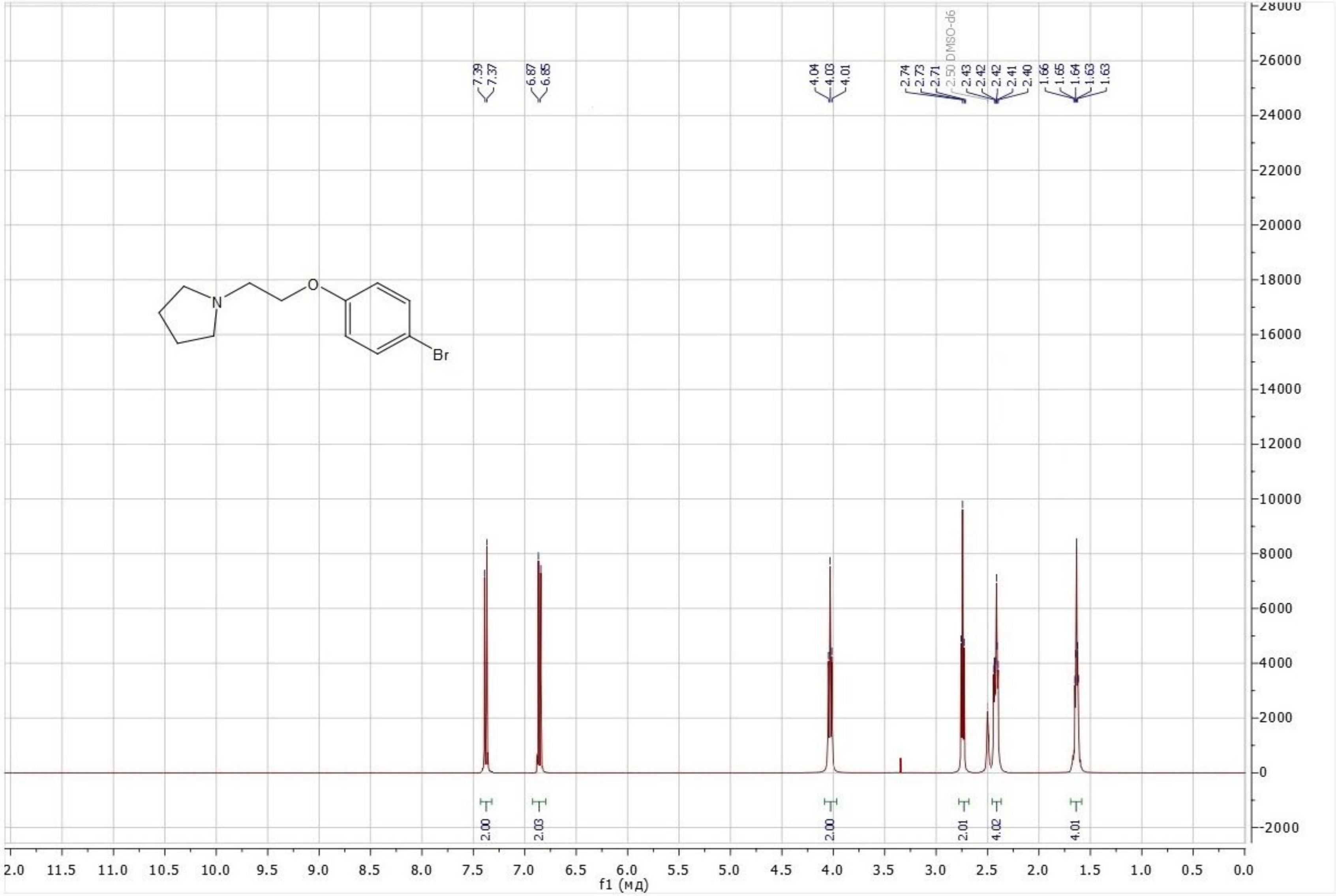

Figure S6. ^{13}C NMR spectrum of **6b**.

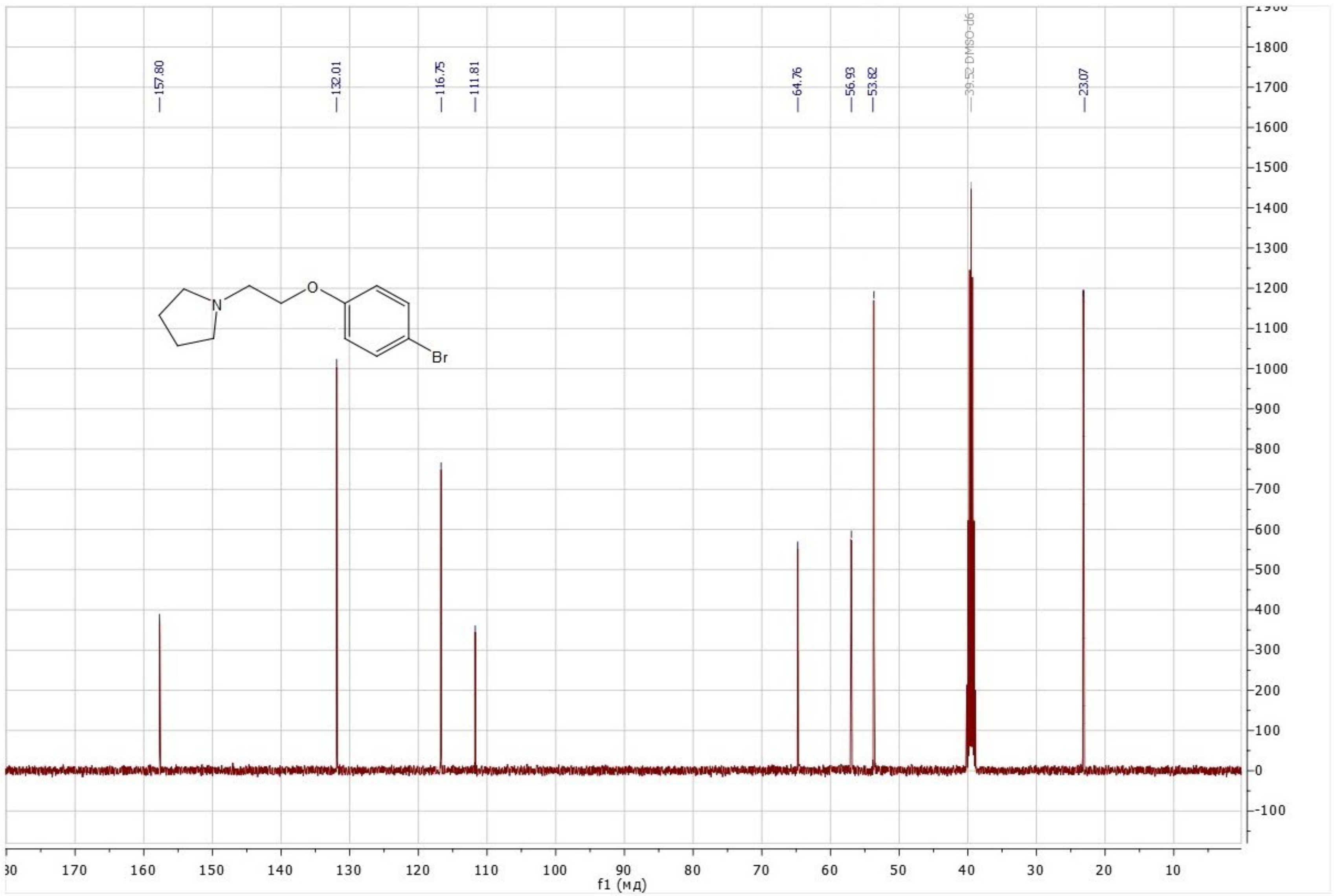

Figure S7. ^{1}H NMR spectrum of **6c**.

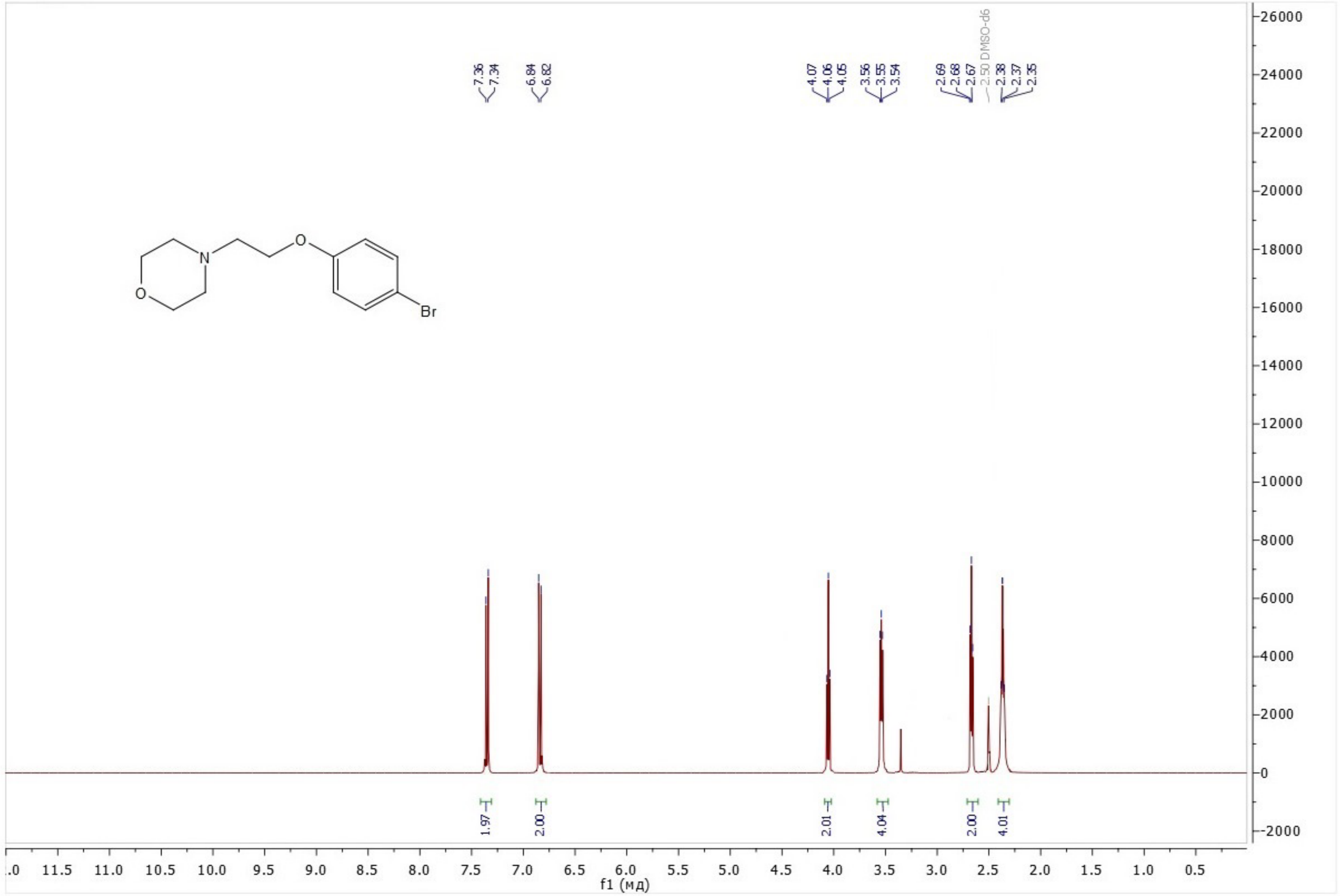

Figure S8. ^{13}C NMR spectrum of **6c**.

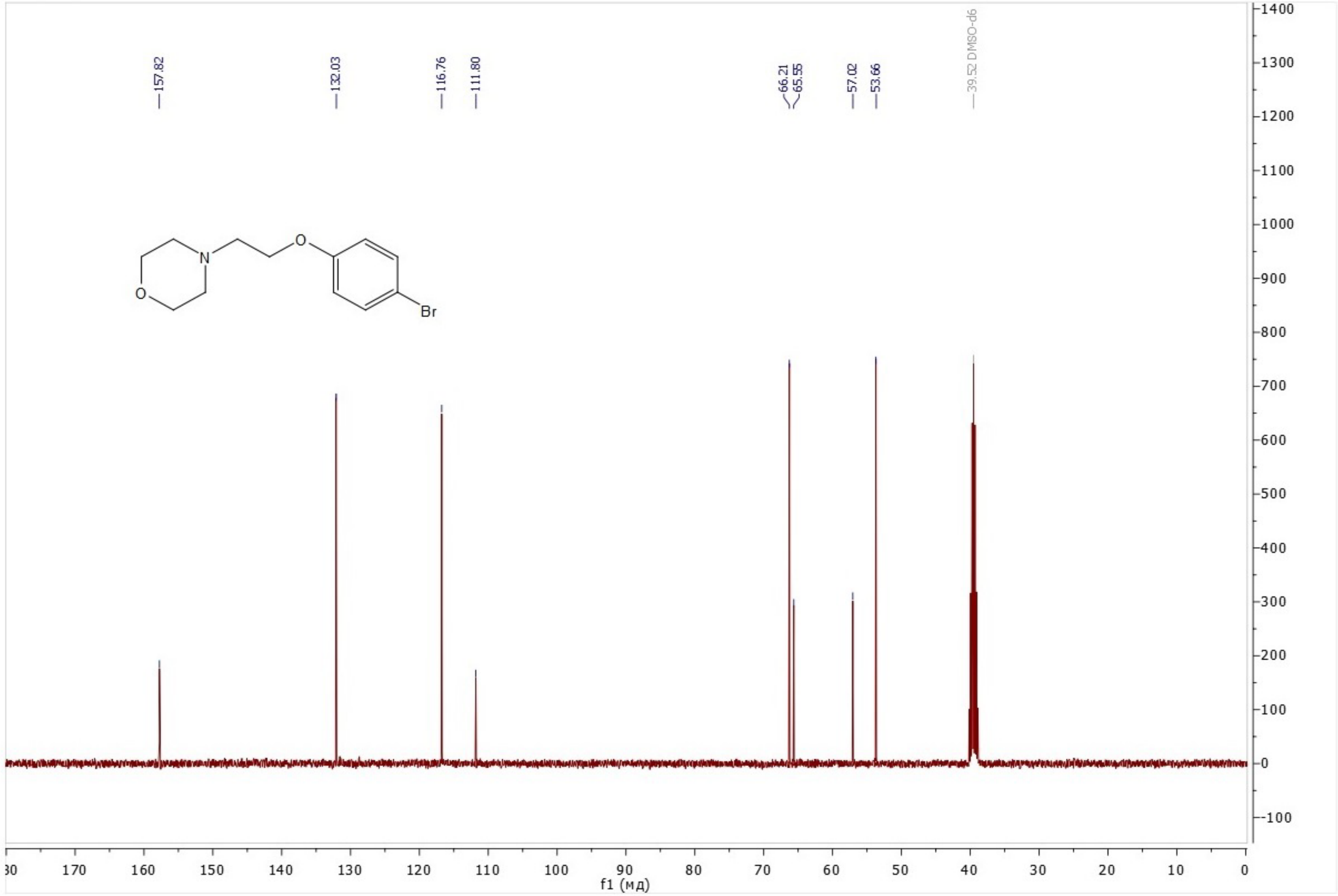

Figure S9. ^{1}H NMR spectrum of **6d**.

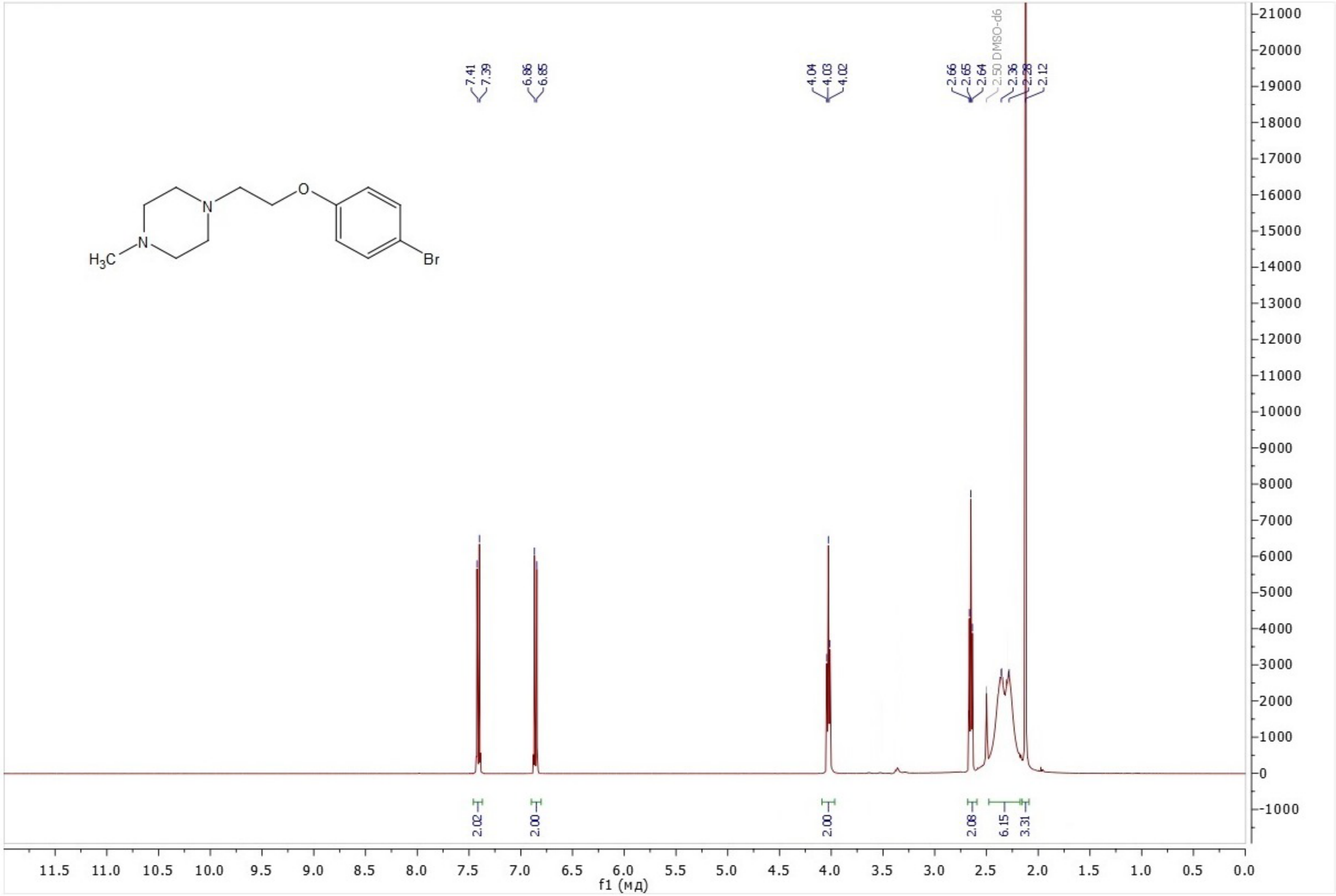

Figure S10. ^{13}C NMR spectrum of **6d**.

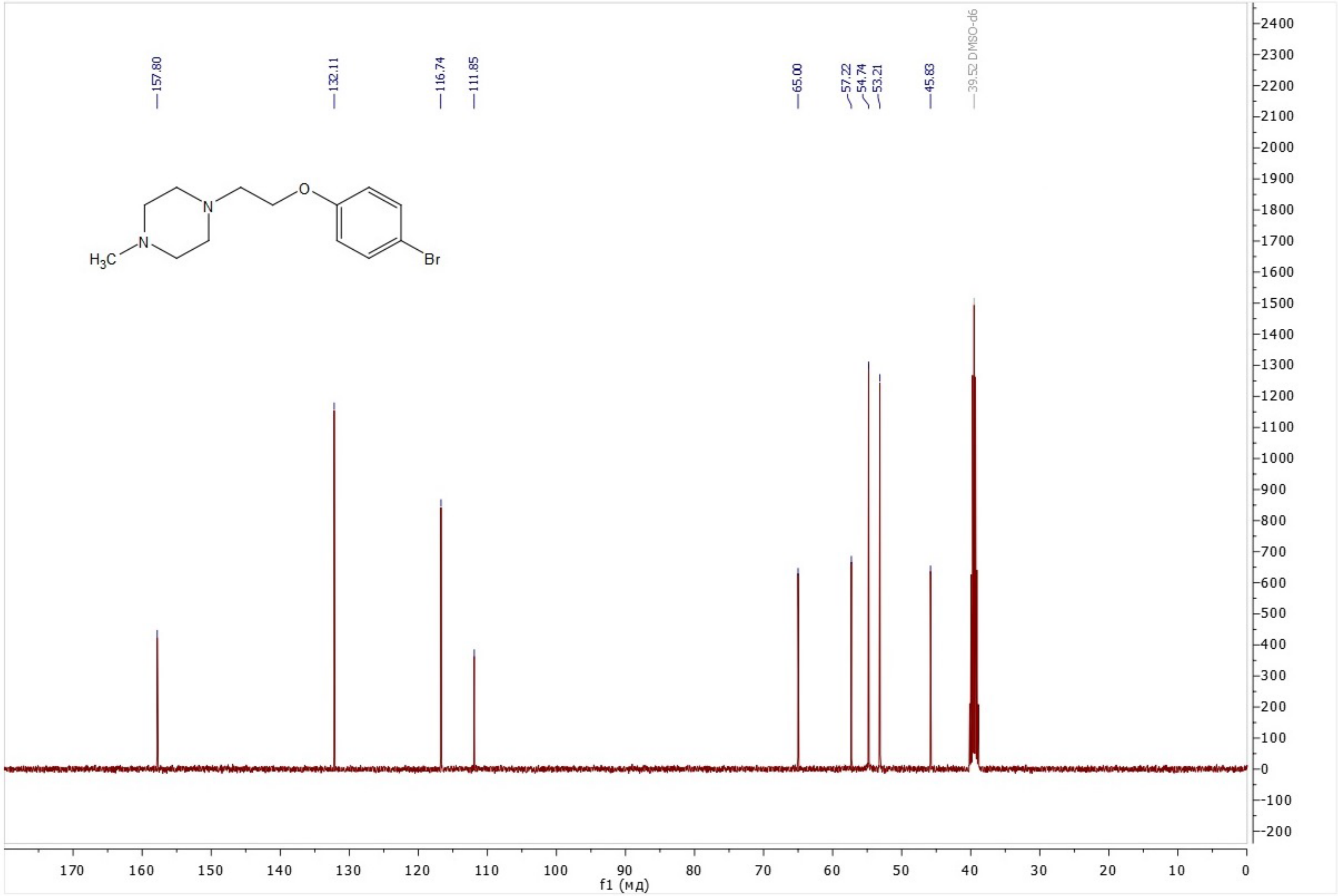

Figure S11. ^{1}H NMR spectrum of **6e**.

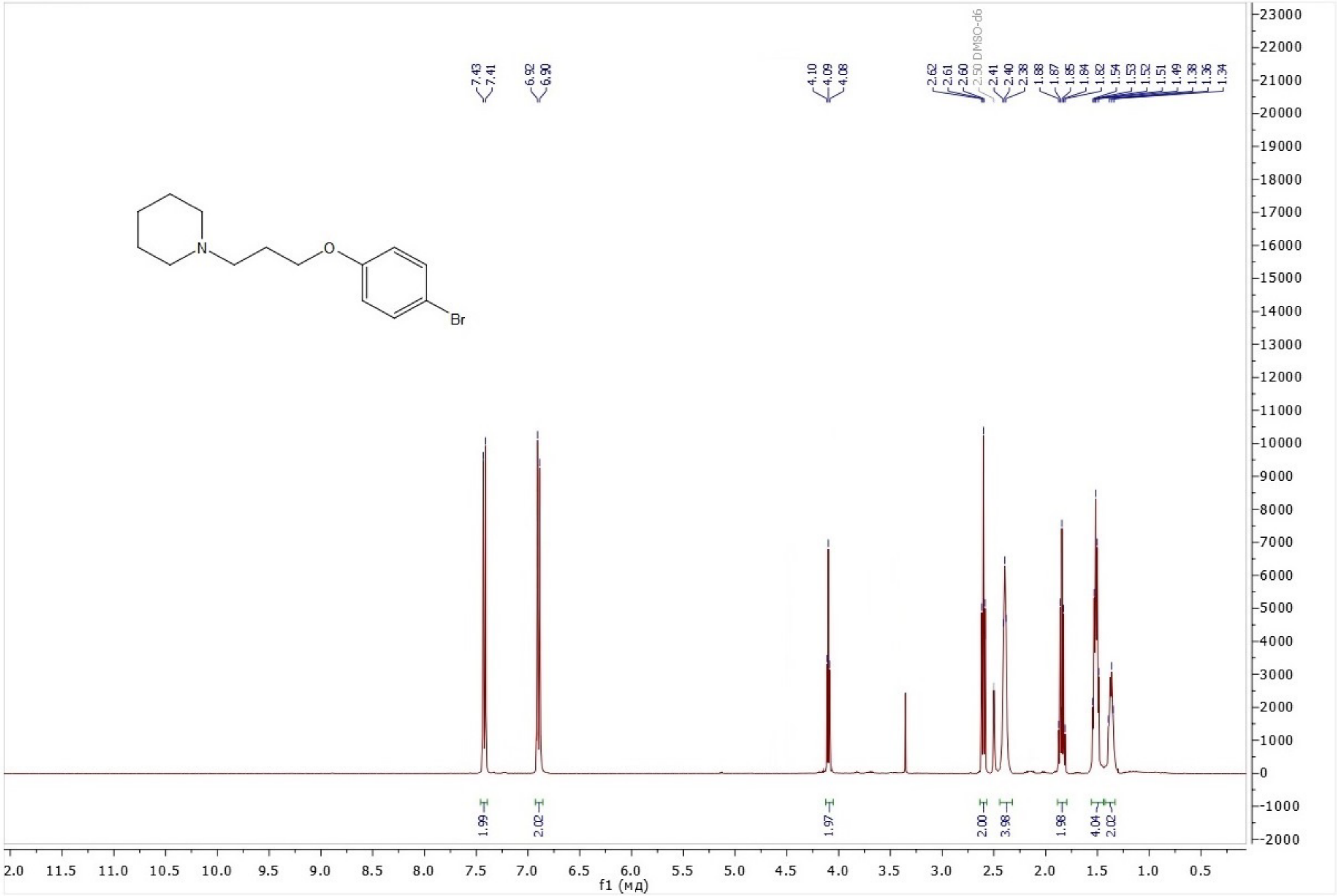

Figure S12. ^{13}C NMR spectrum of **6e**.

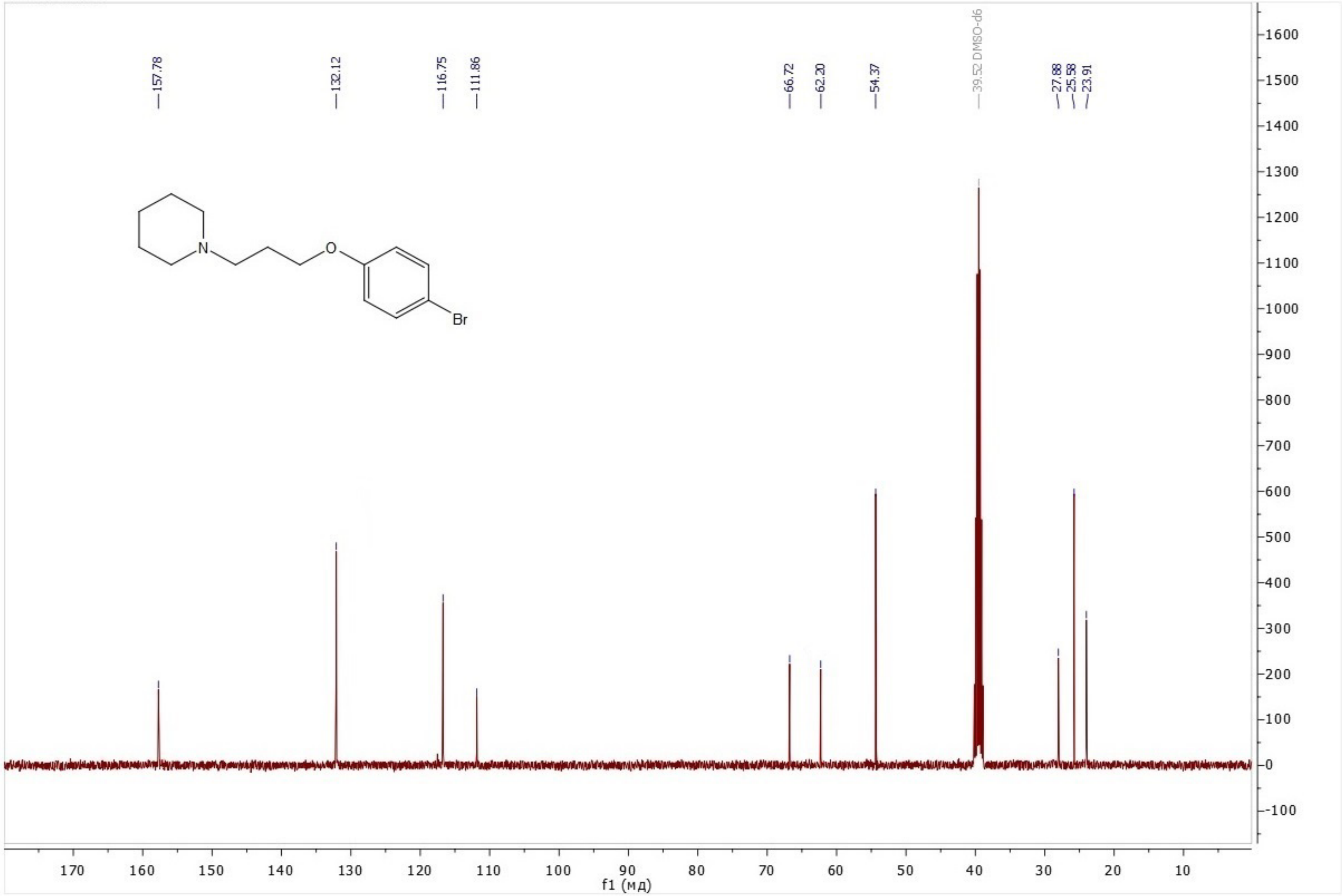

Figure S13. ^{1}H NMR spectrum of **6f**.

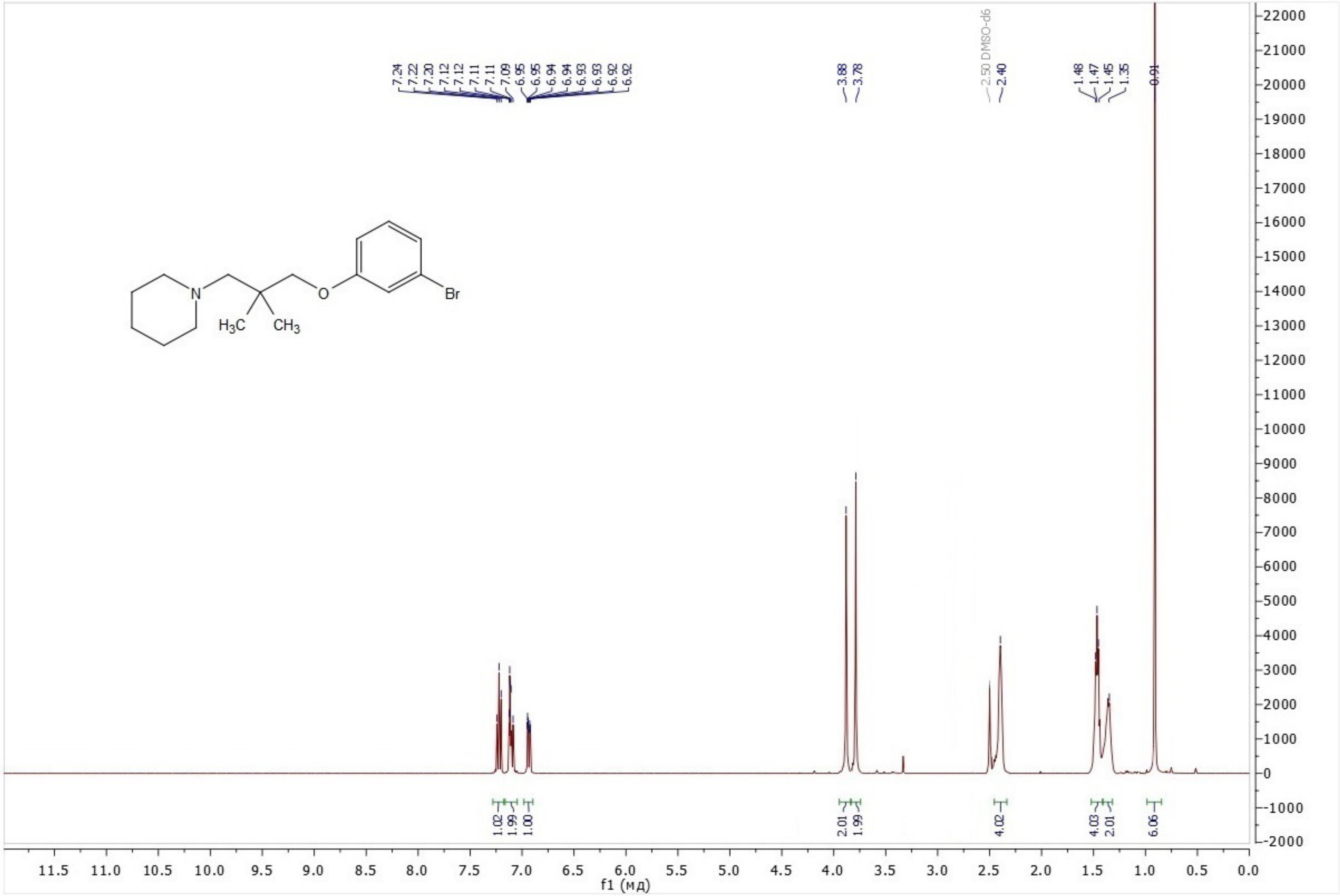

Figure S14. ^{13}C NMR spectrum of **6f**.

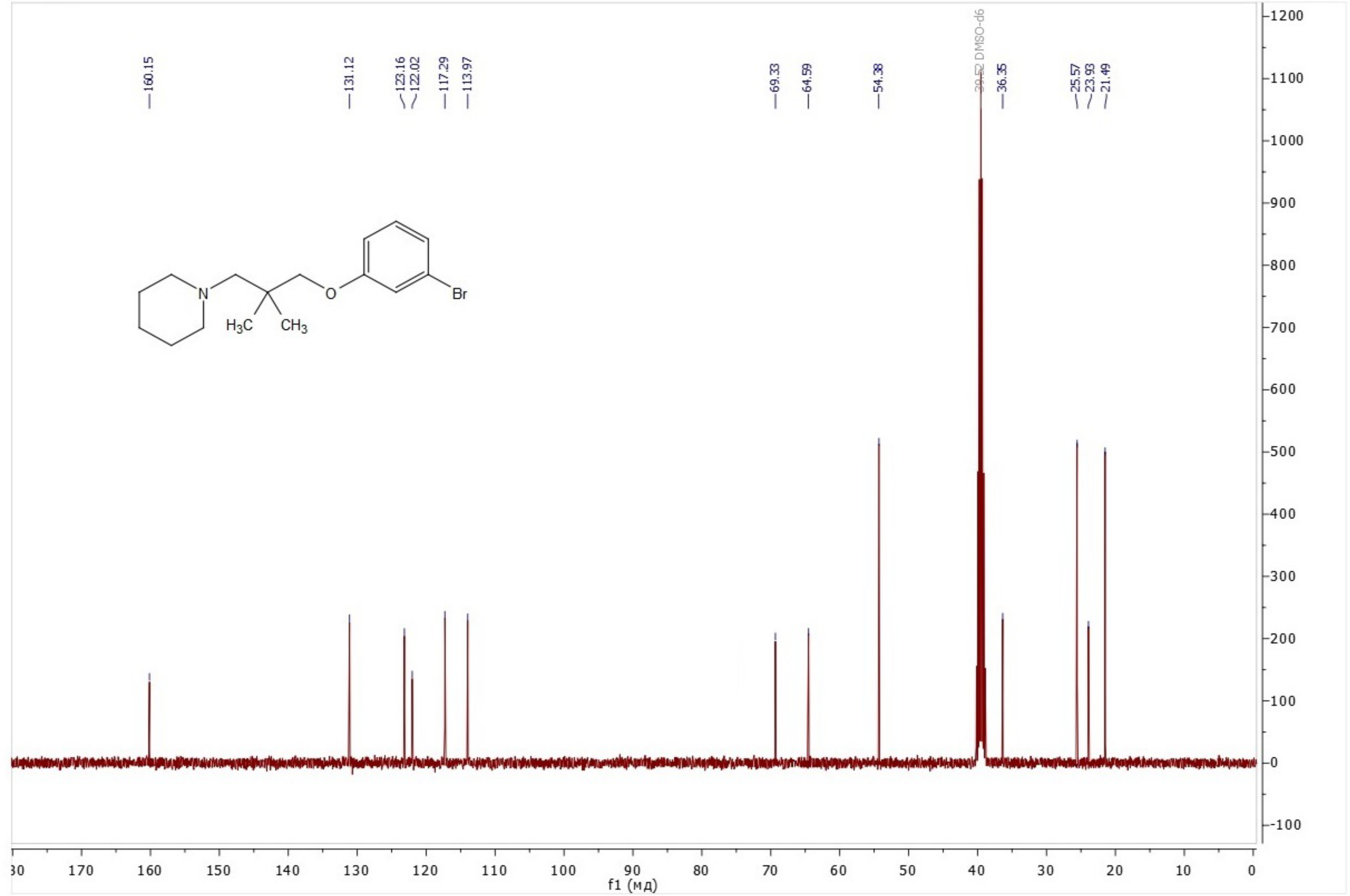

Figure S15. ^{1}H NMR spectrum of **6g**.

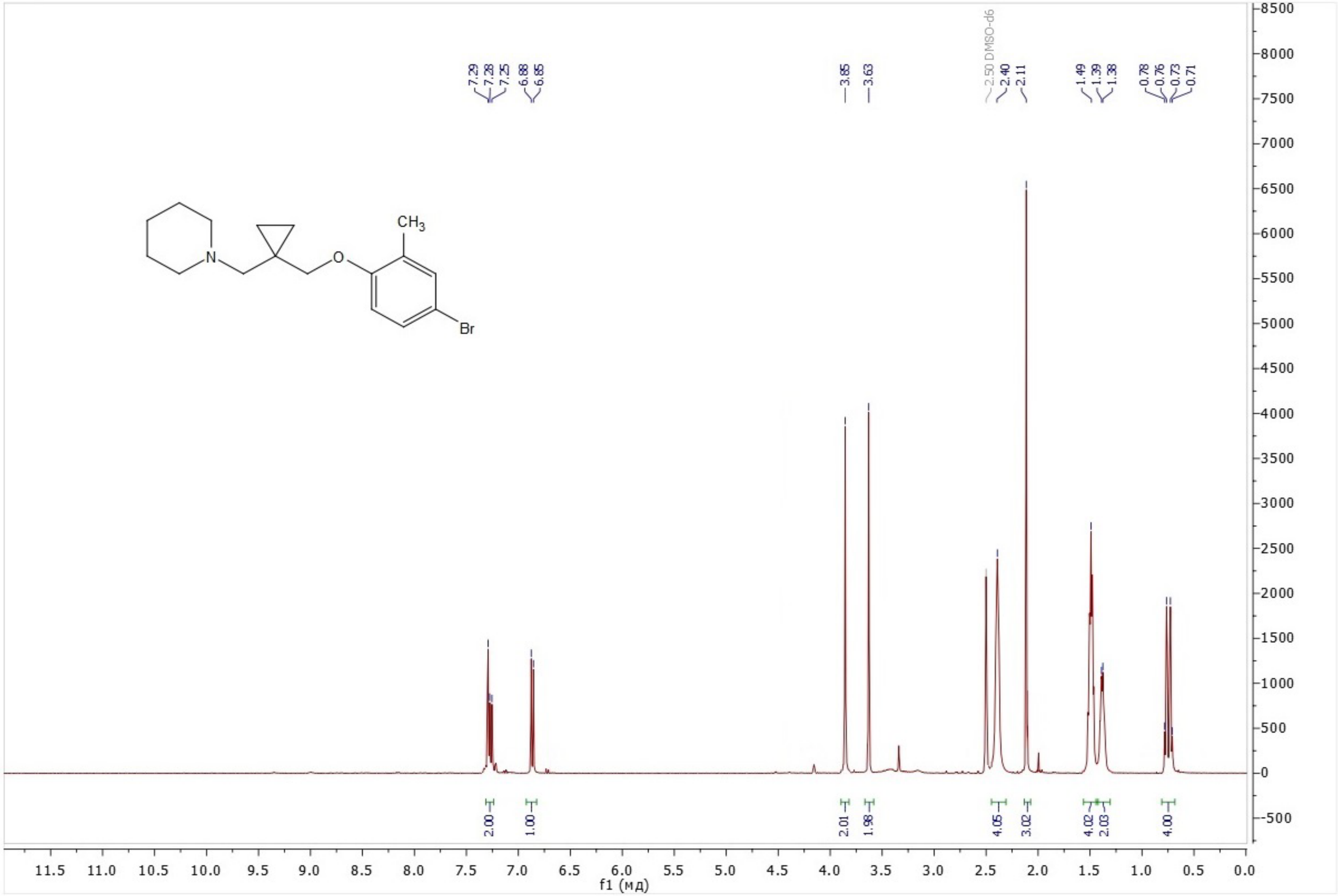

Figure S16. ^{13}C NMR spectrum of **6g**.

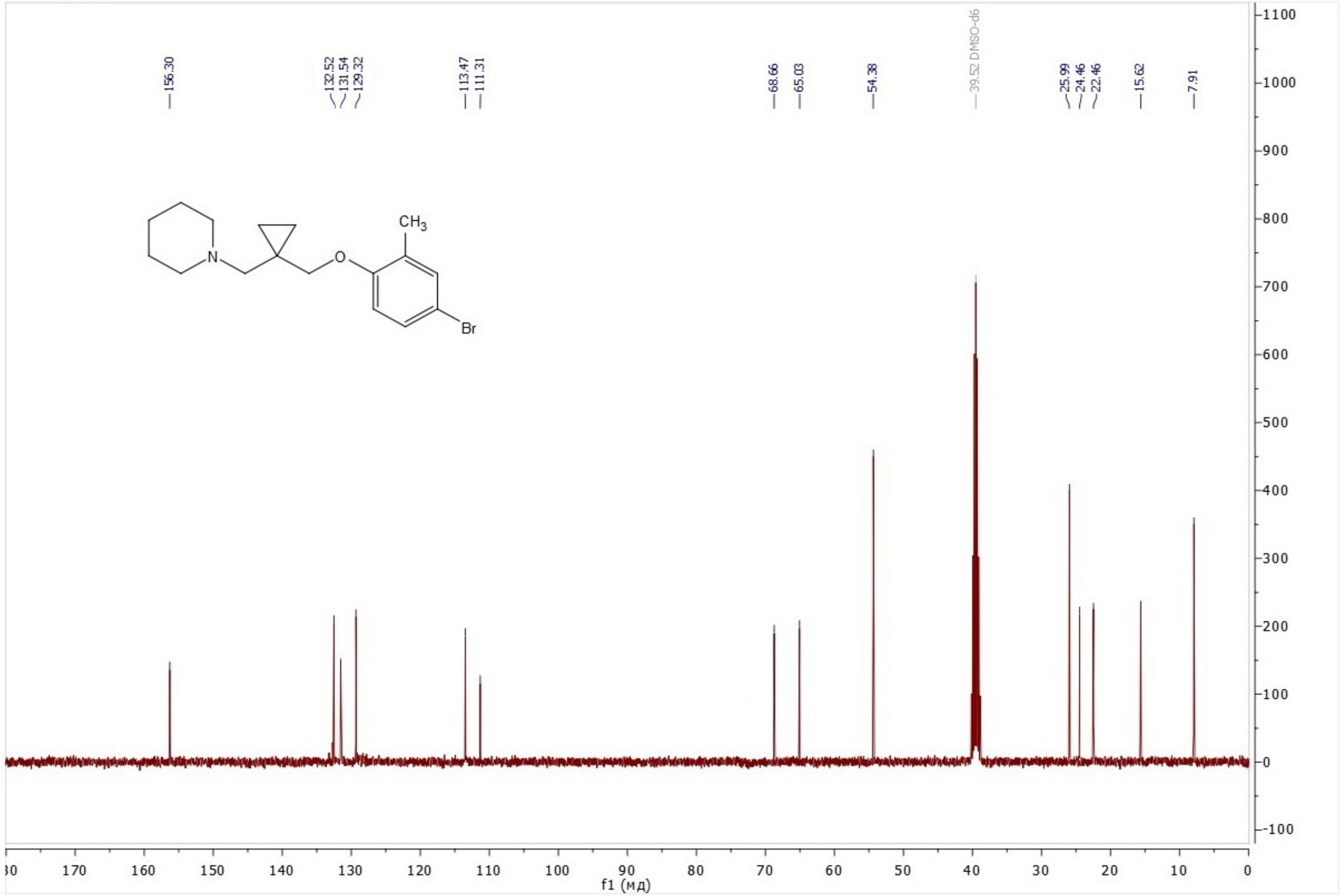

Figure S17. ^{1}H NMR spectrum of **6h**.

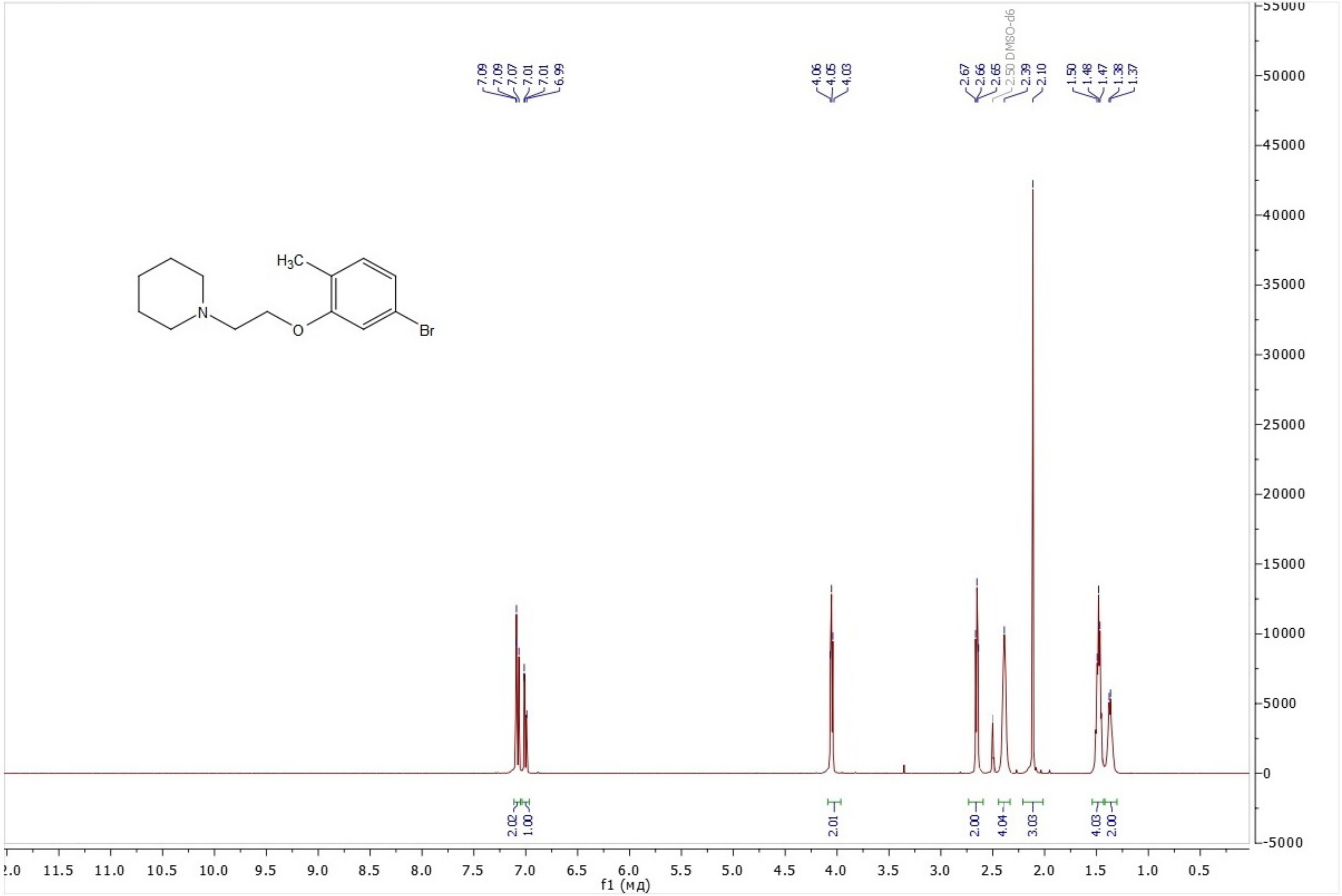

Figure S18. ^{13}C NMR spectrum of **6h**.

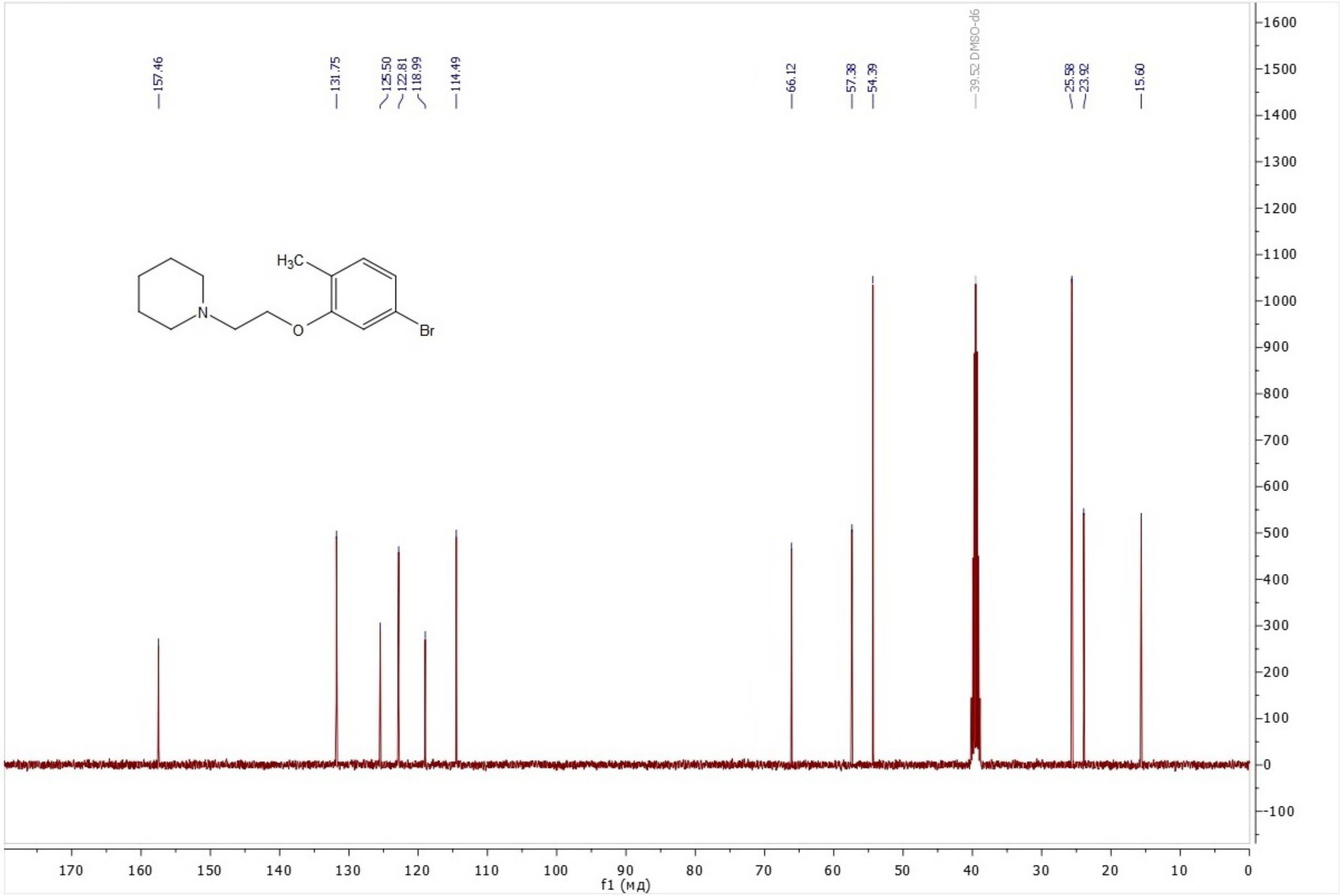

Figure S19. ^{1}H NMR spectrum of **6i**.

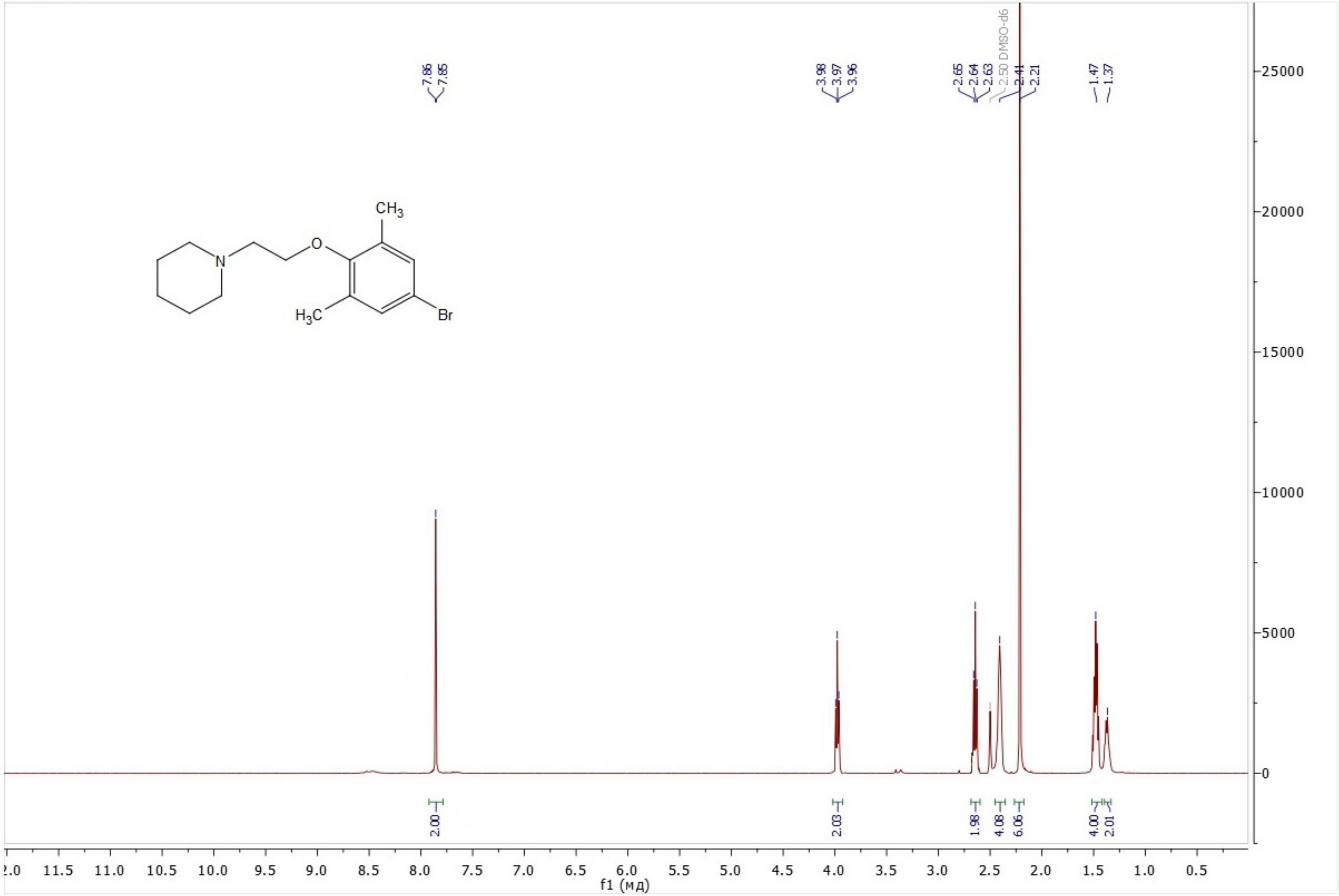

Figure S20. ^{13}C NMR spectrum of **6i**.

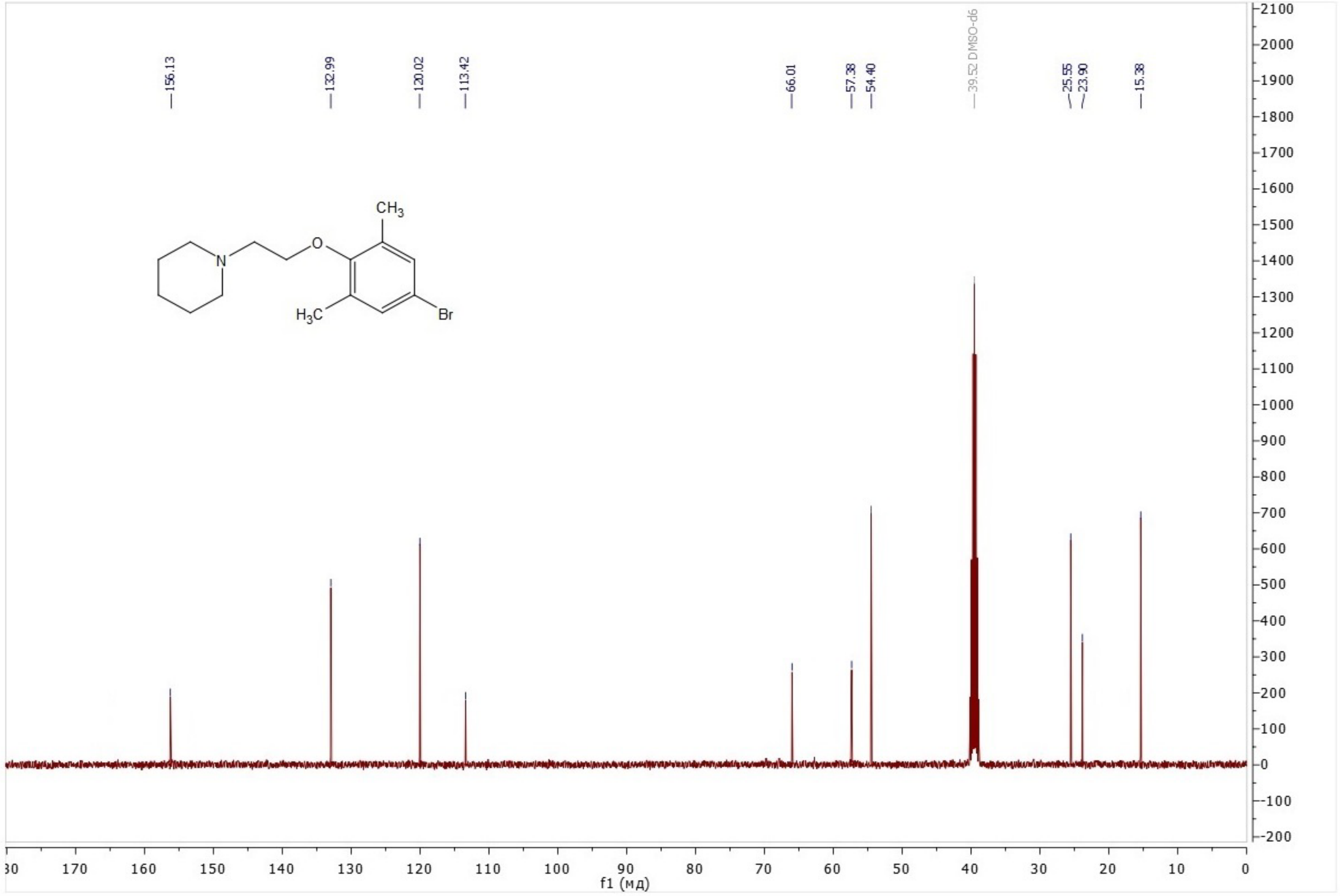

Figure S21. ^{1}H NMR spectrum of **15a**.

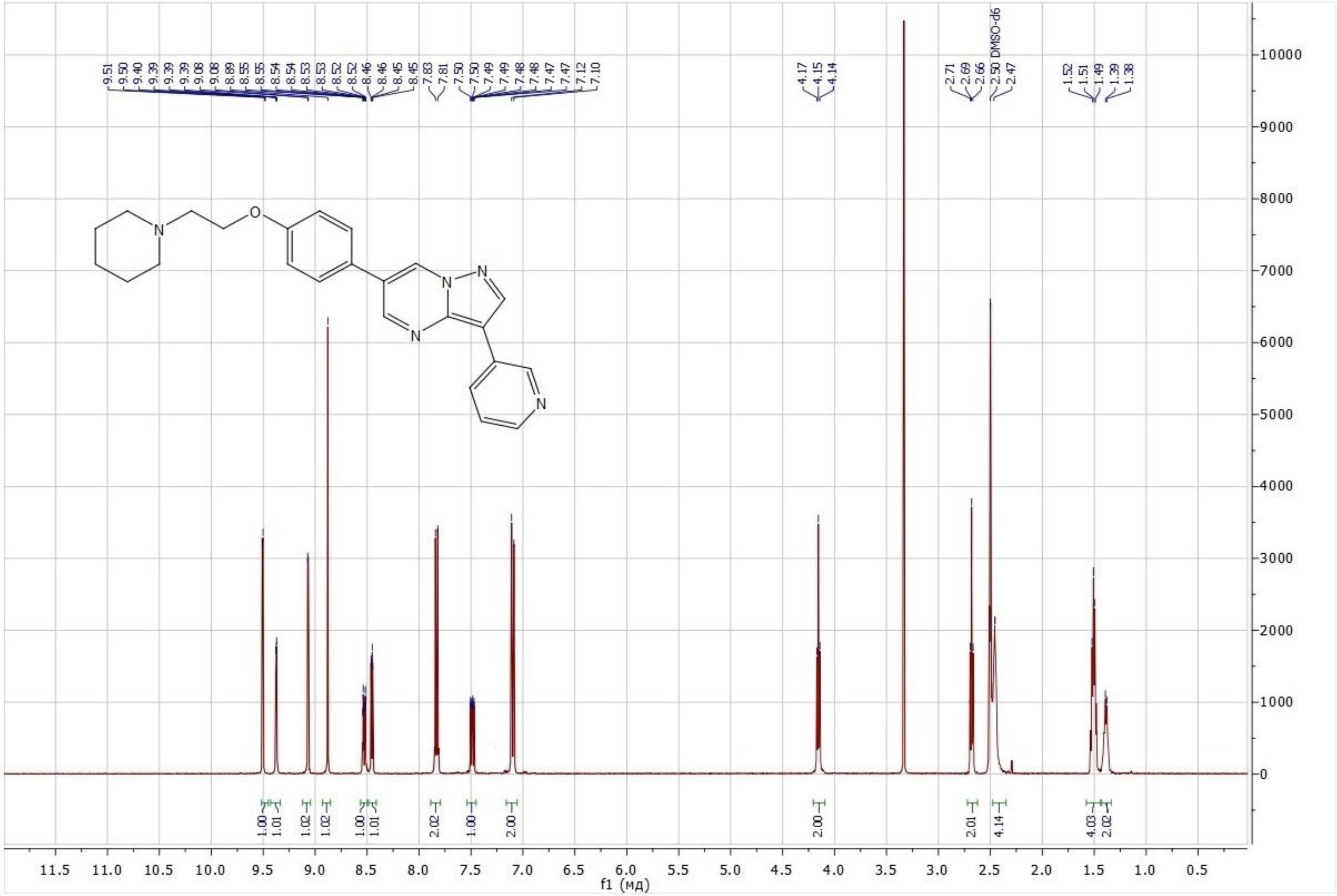

Figure S22. ^{13}C NMR spectrum of **15a**.

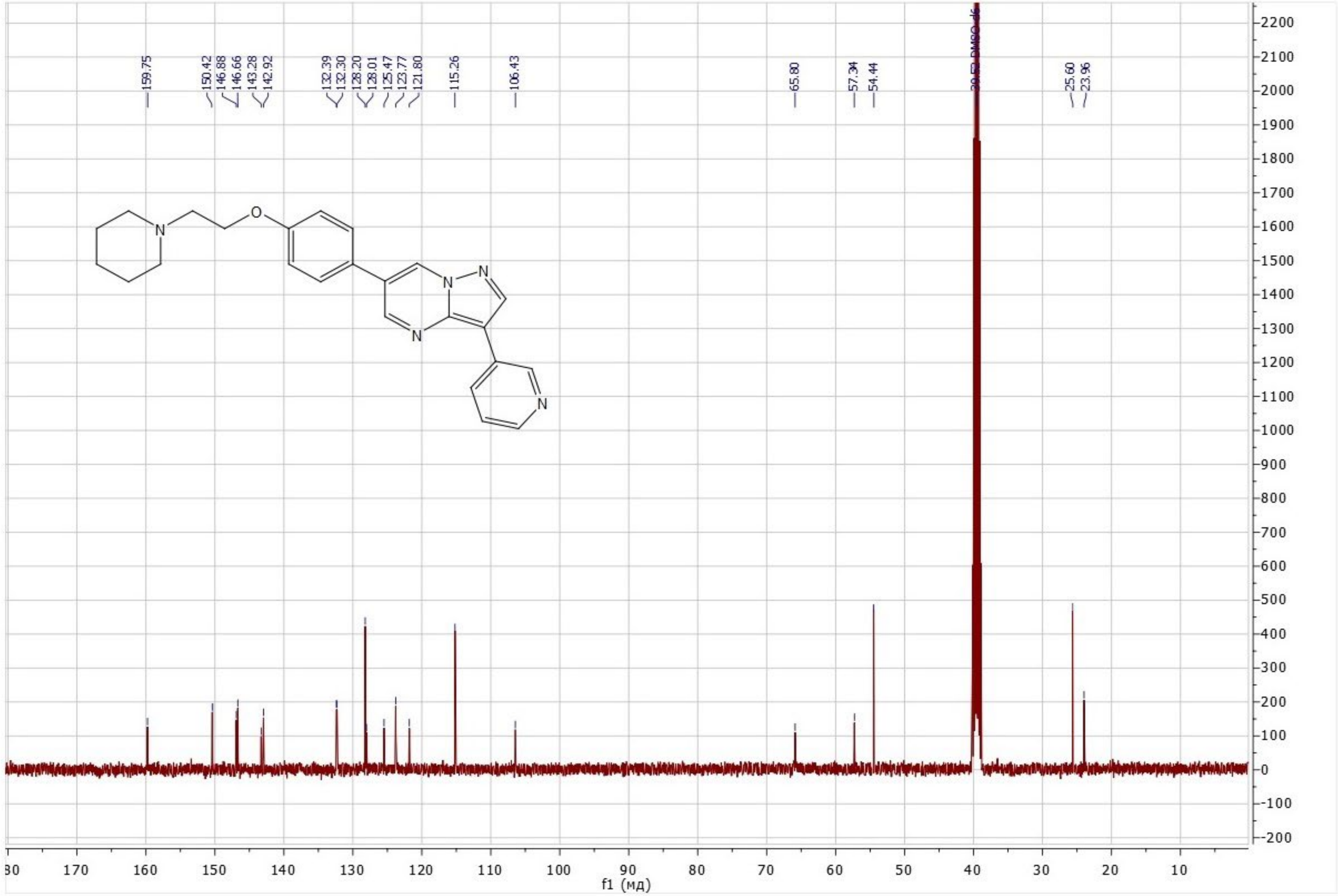

Figure S23. ^{1}H NMR spectrum of **15b**.

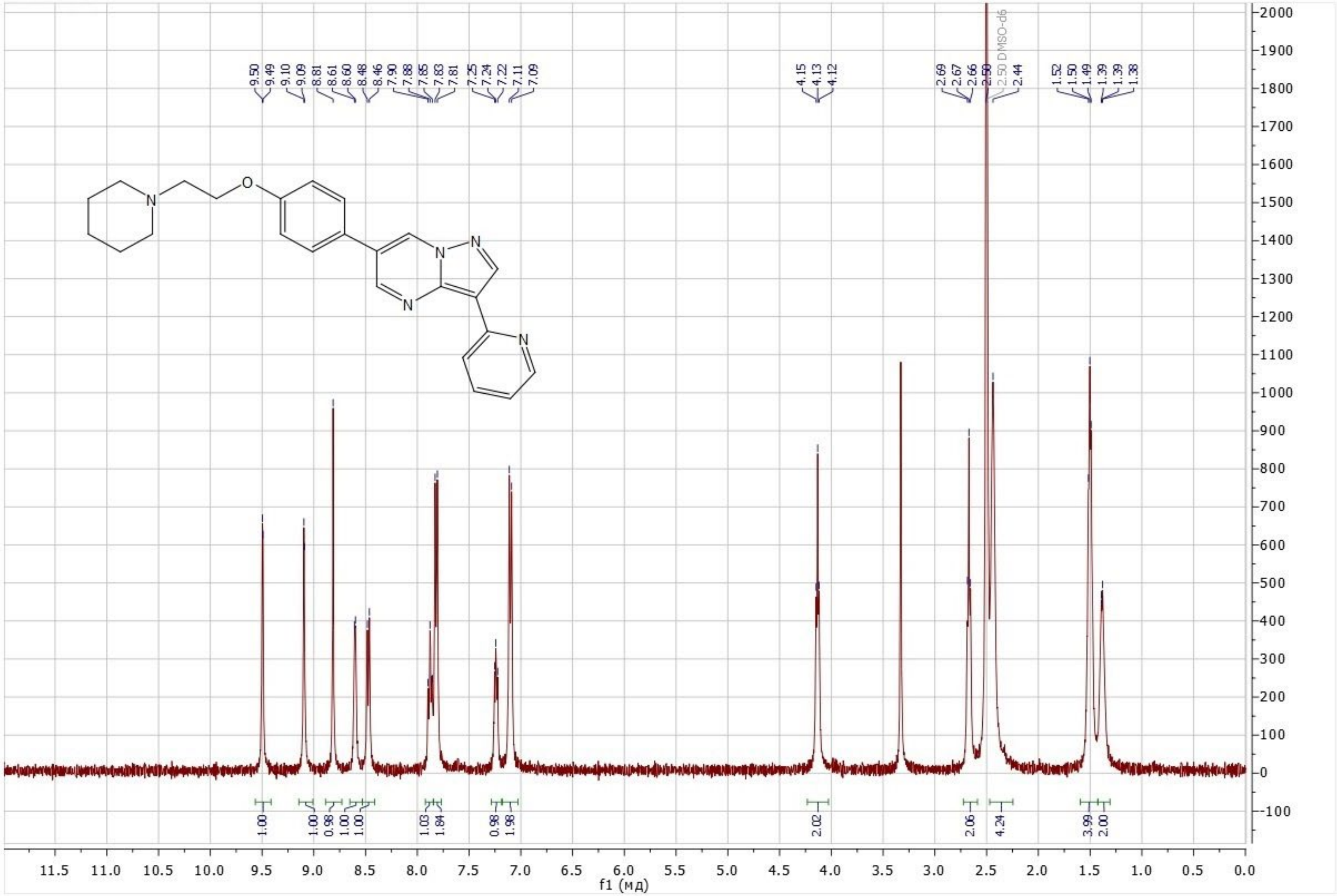

Figure S24. ^{13}C NMR spectrum of **15b**.

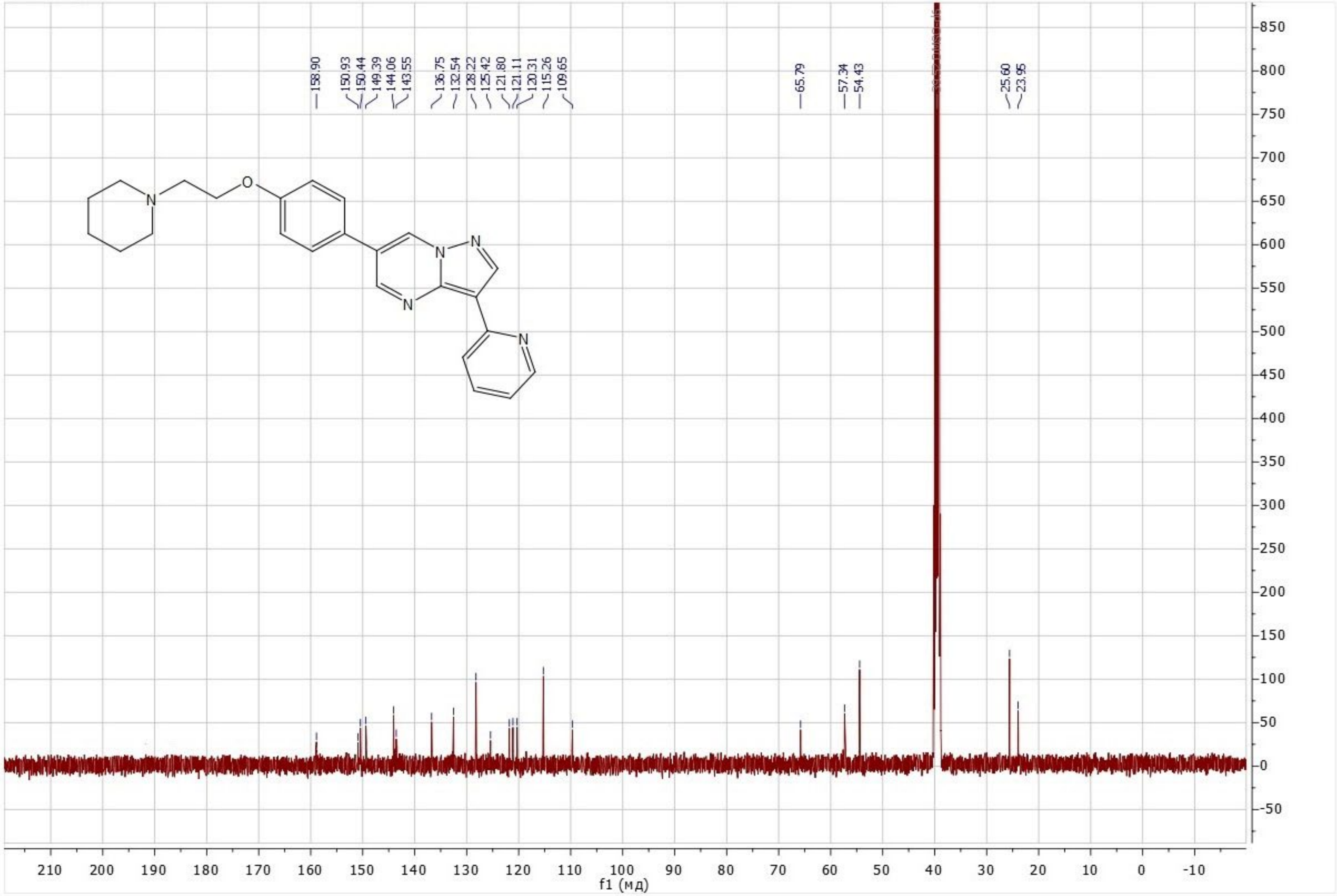

Figure S25. ^{1}H NMR spectrum of **15c**.

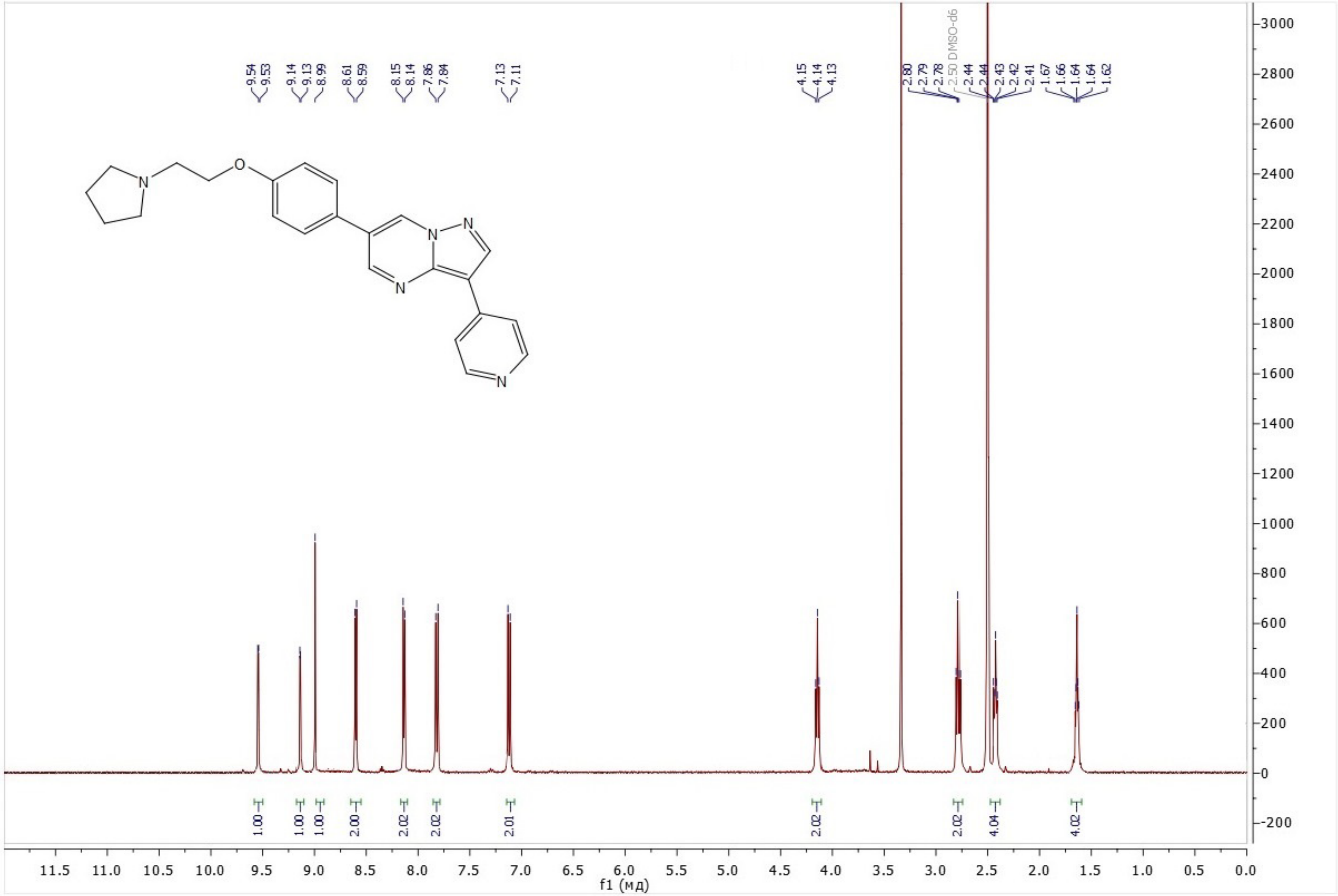

Figure S26. ^{13}C NMR spectrum of **15c**.

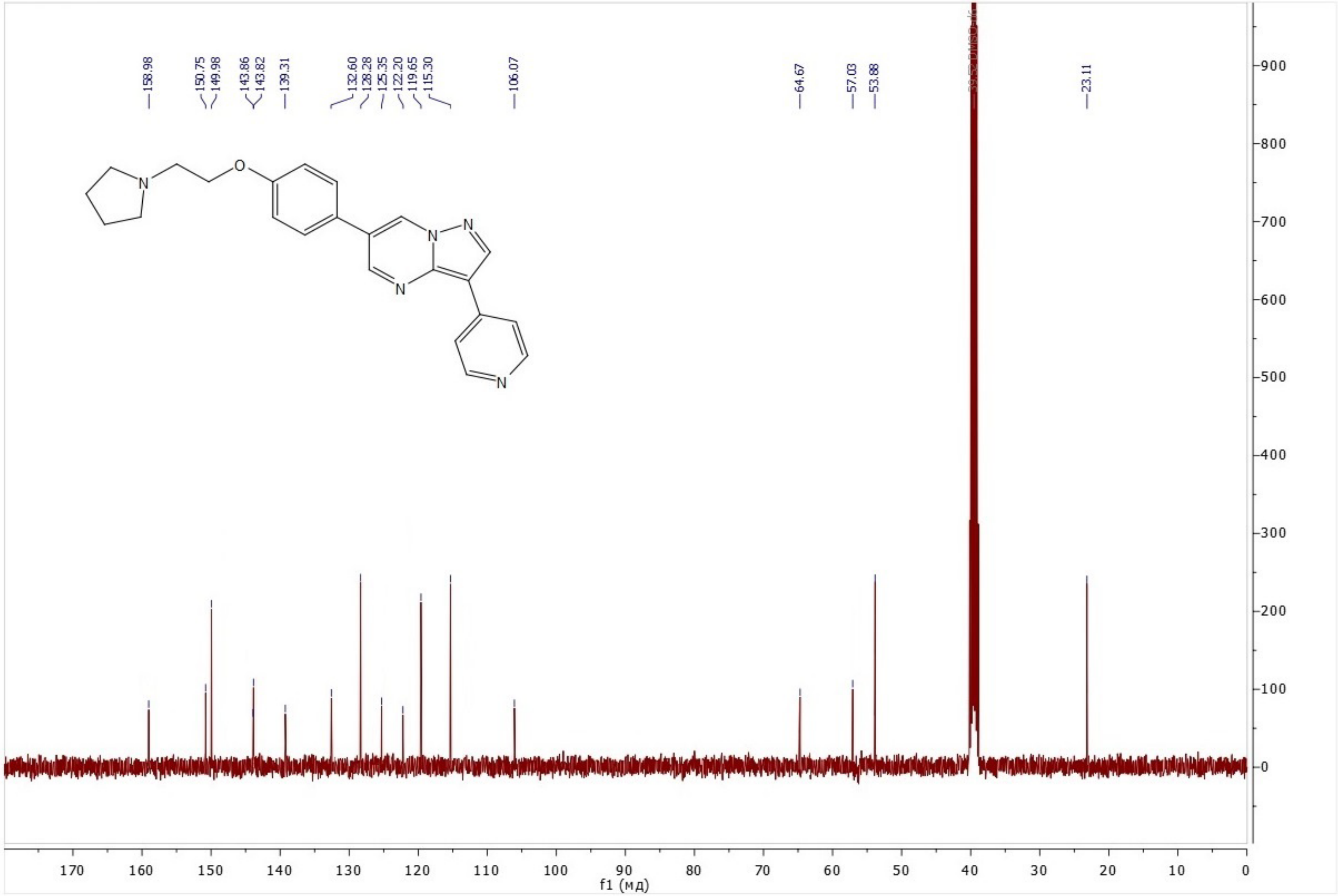

Figure S27. ^{1}H NMR spectrum of **15d**.

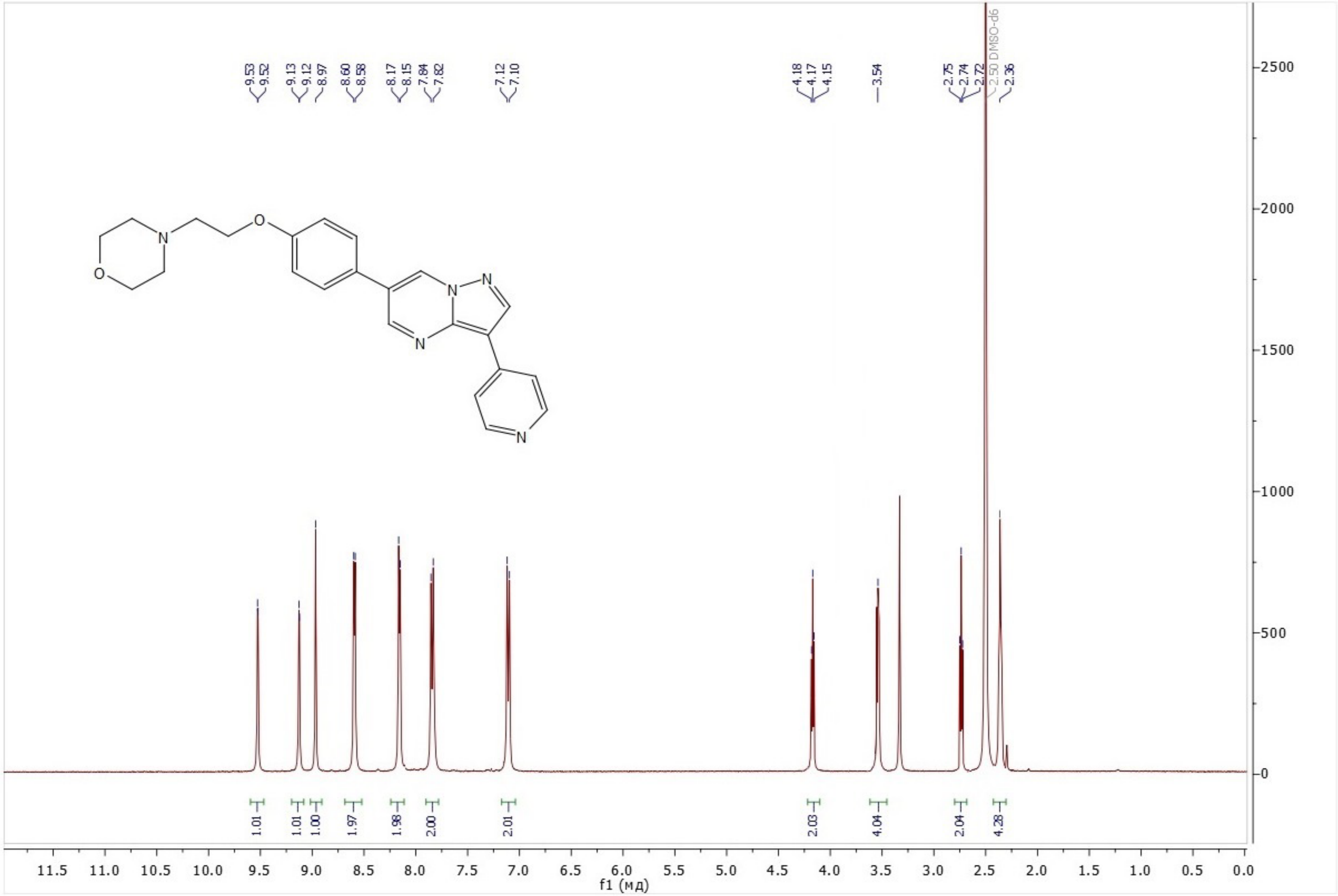

Figure S28. ^{13}C NMR spectrum of **15d**.

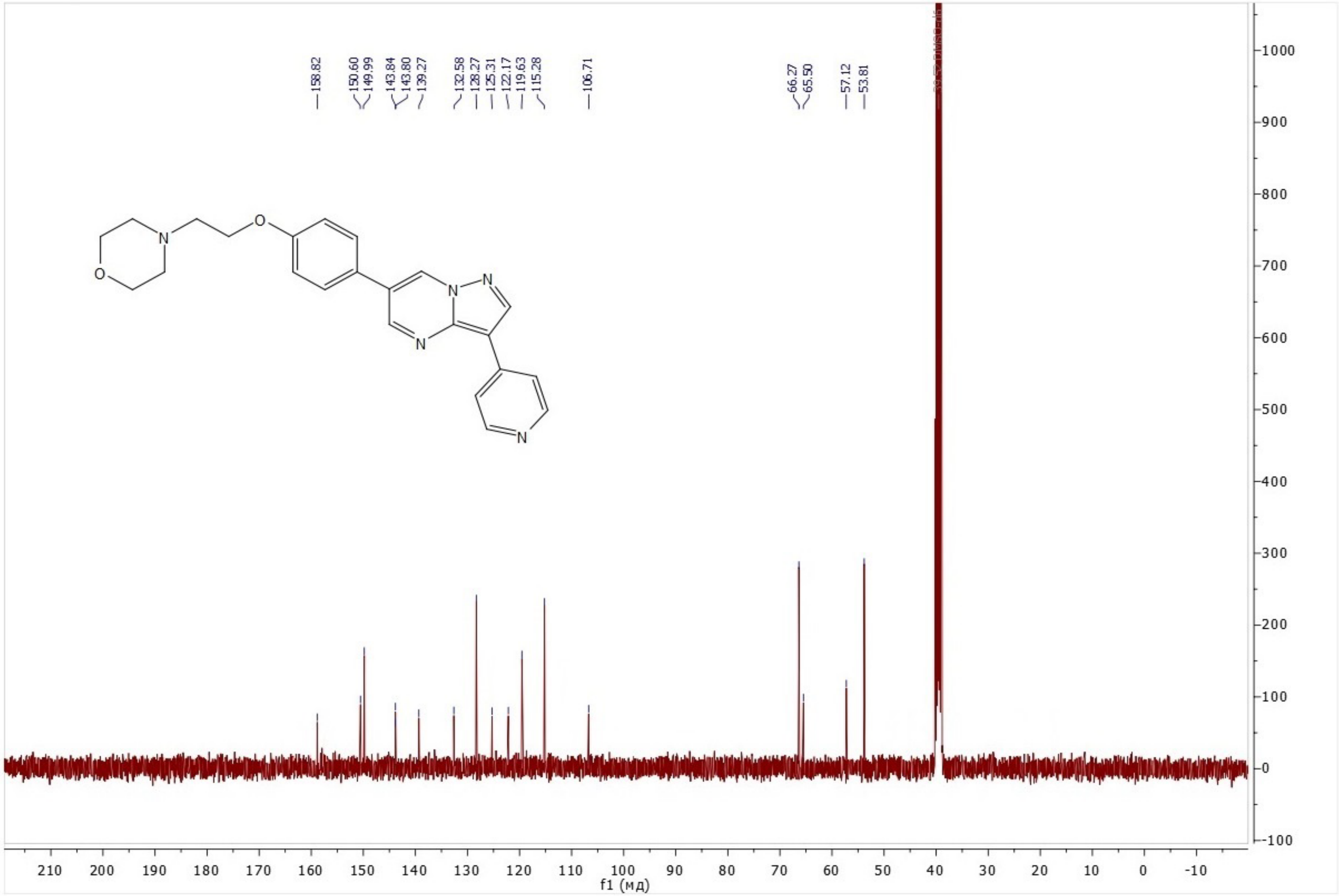

Figure S29. ^{1}H NMR spectrum of **15e**.

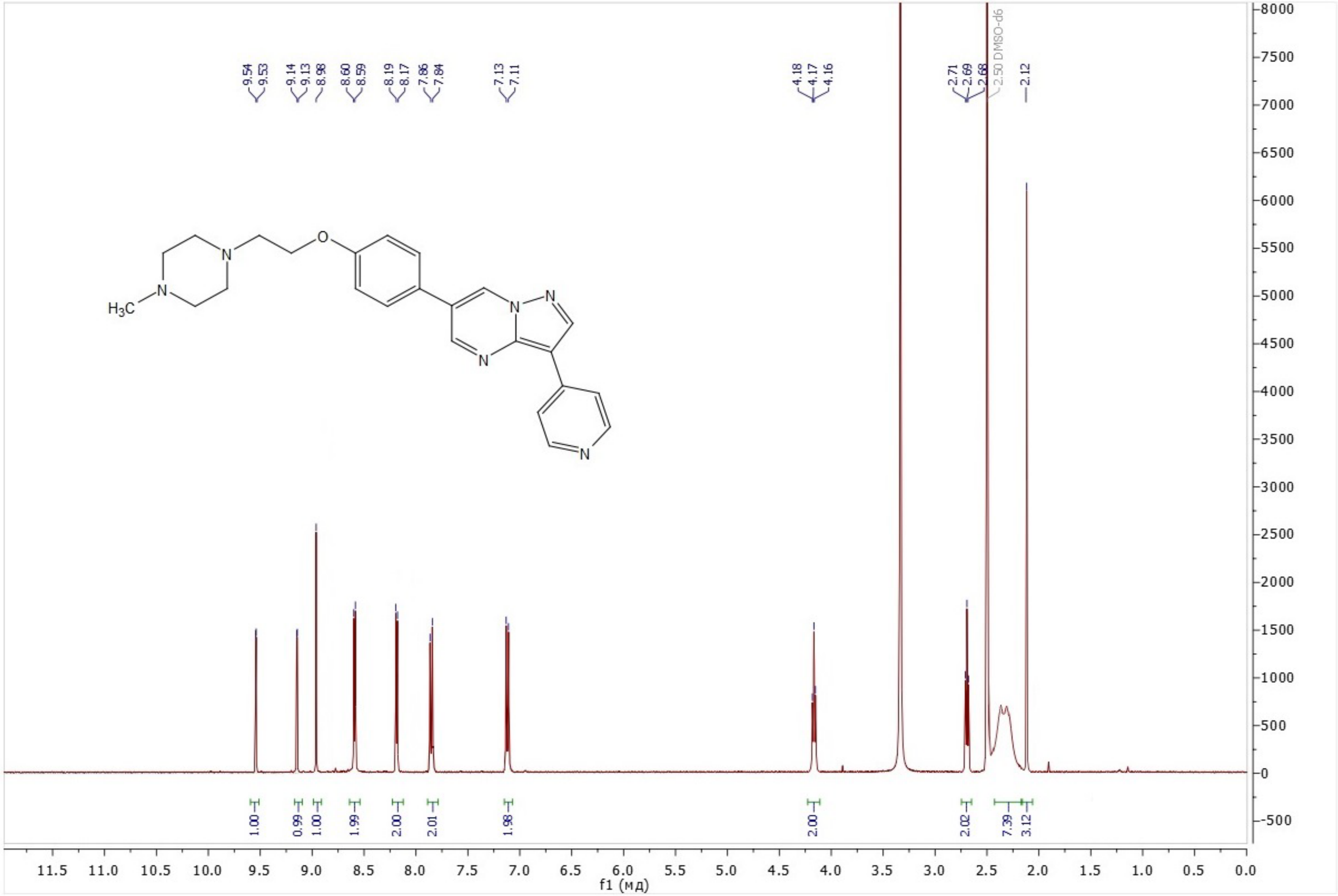

Figure S30. ^{13}C NMR spectrum of **15e**.

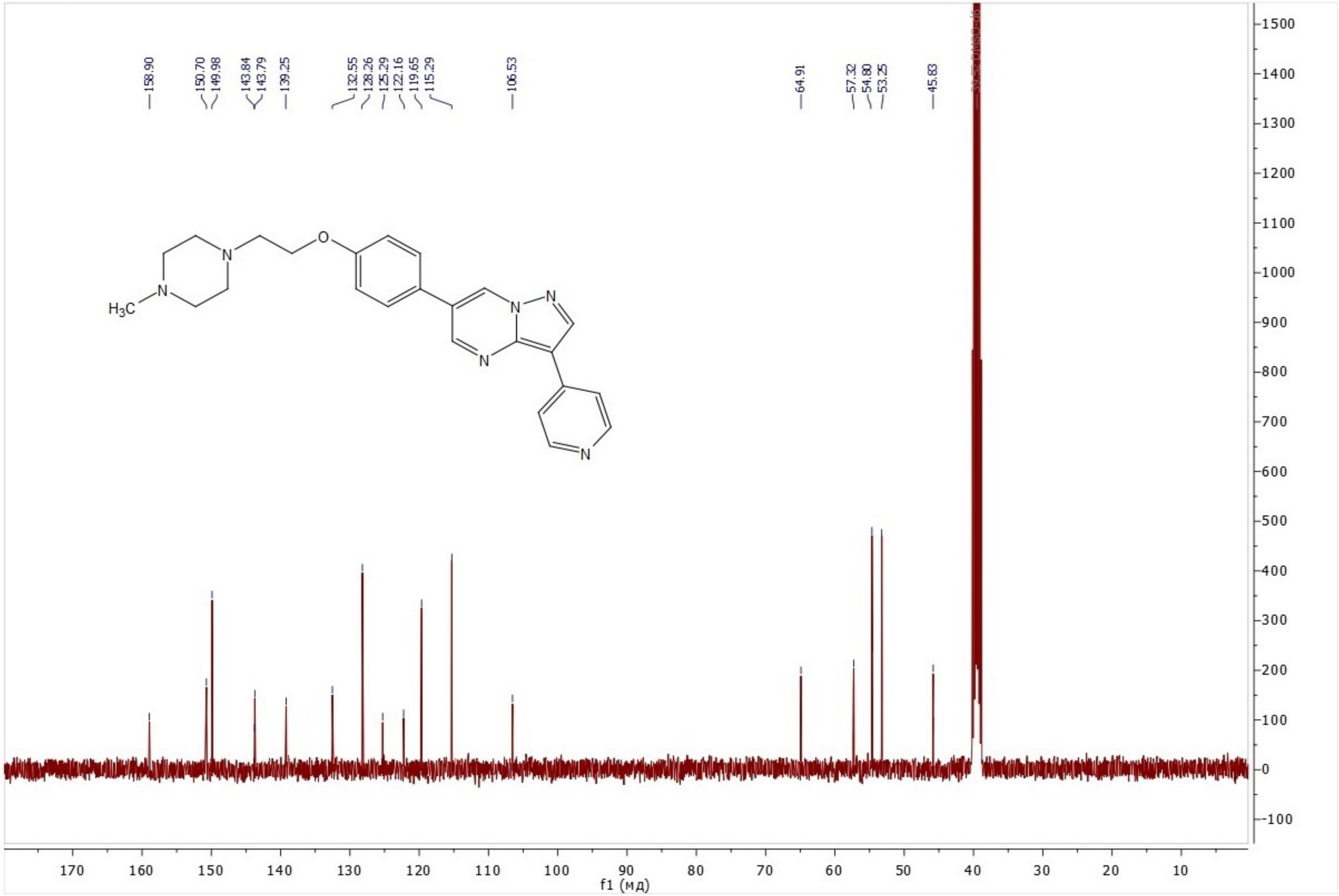

Figure S31. ^{1}H NMR spectrum of **15f**.

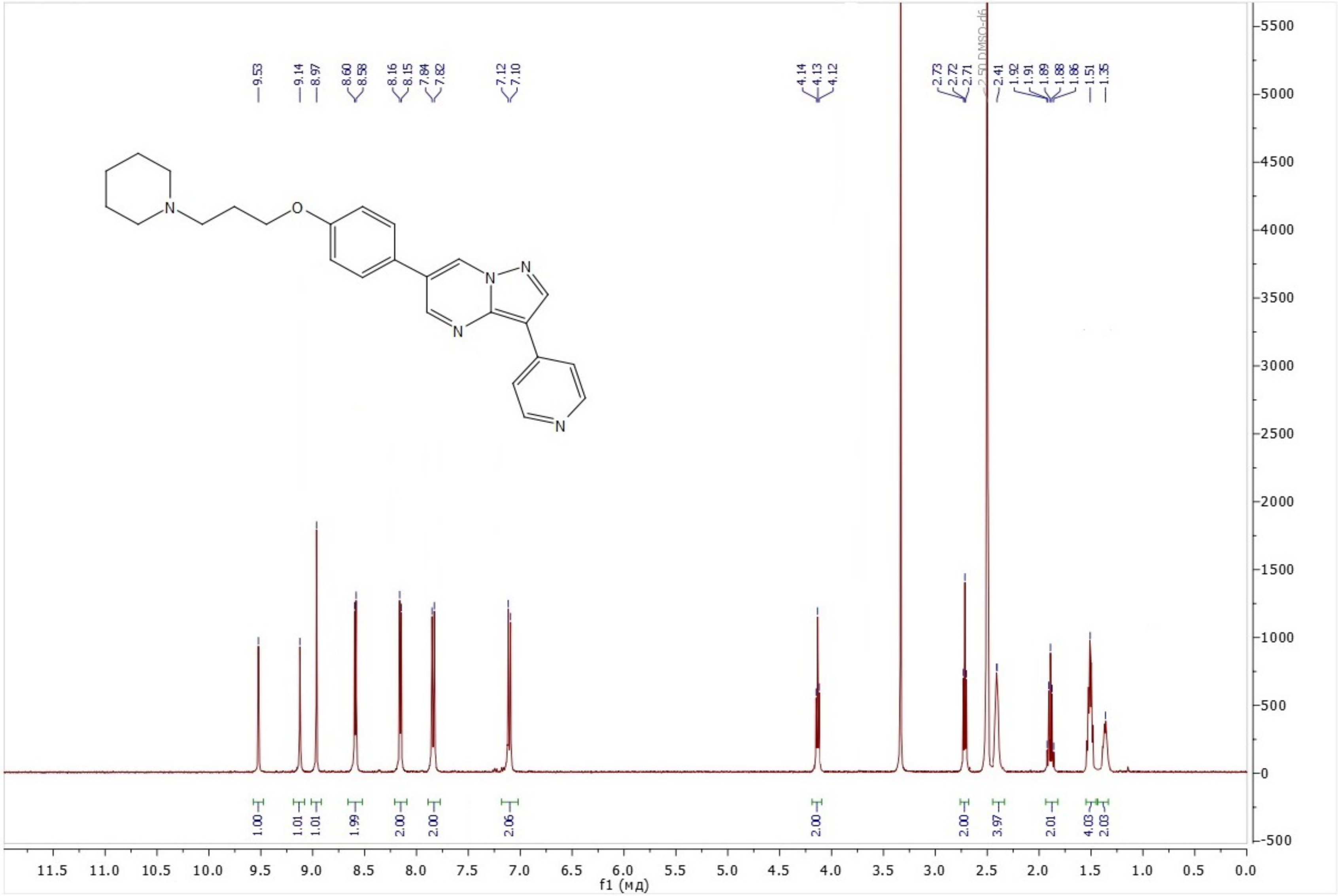

Figure S32. ^{13}C NMR spectrum of **15f**.

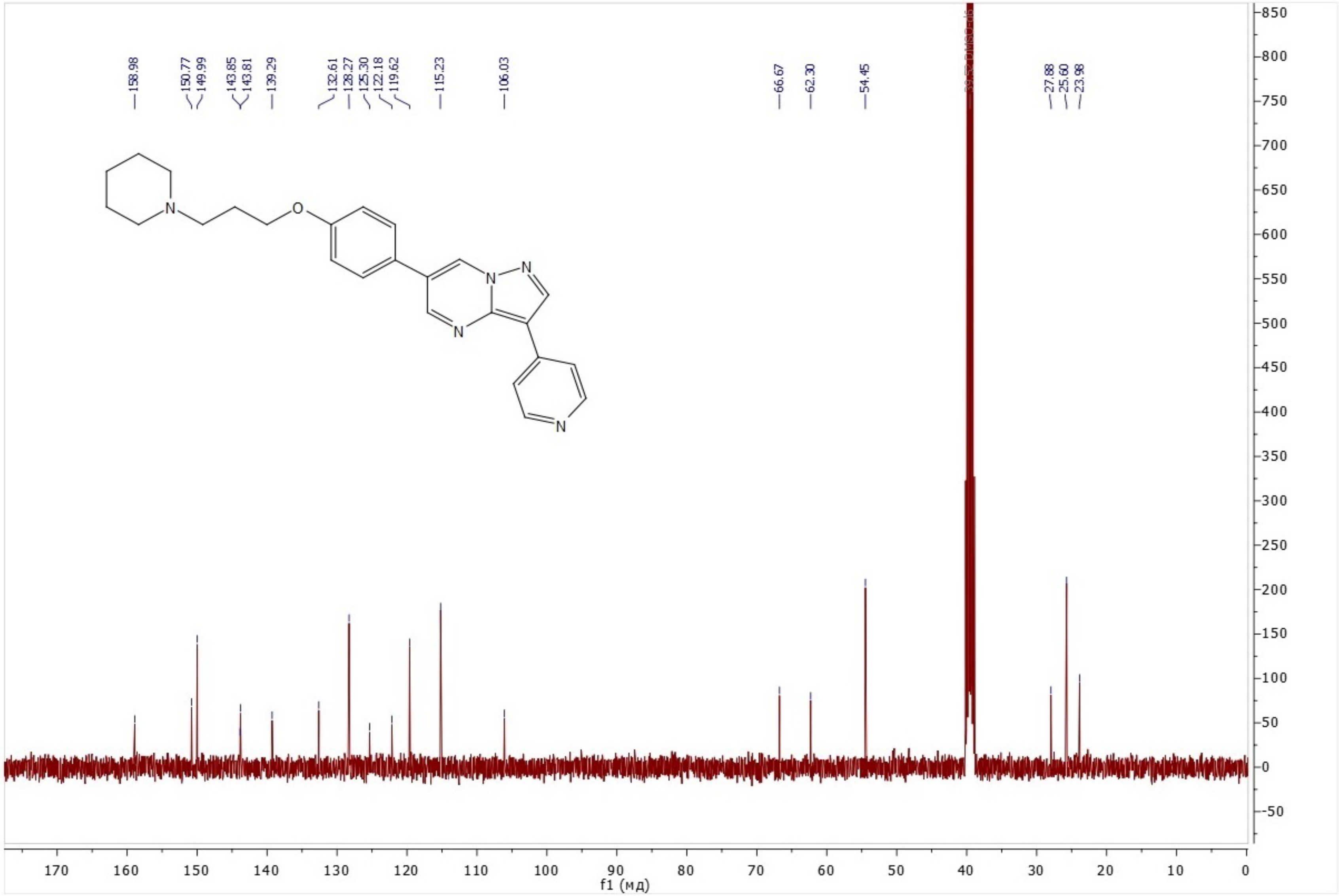

Figure S33. ^{1}H NMR spectrum of **15g**.

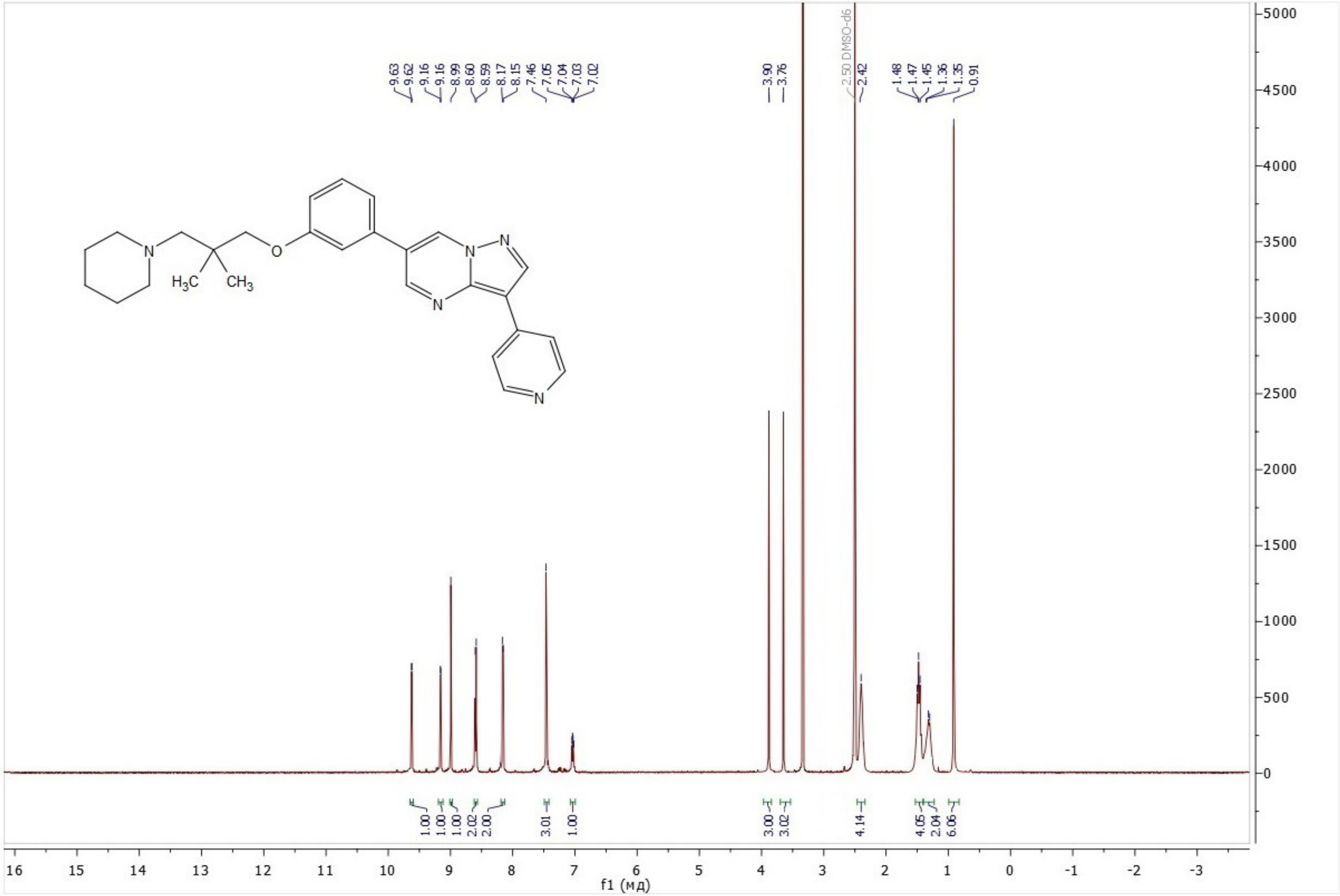

Figure S34. ^{13}C NMR spectrum of **15g**.

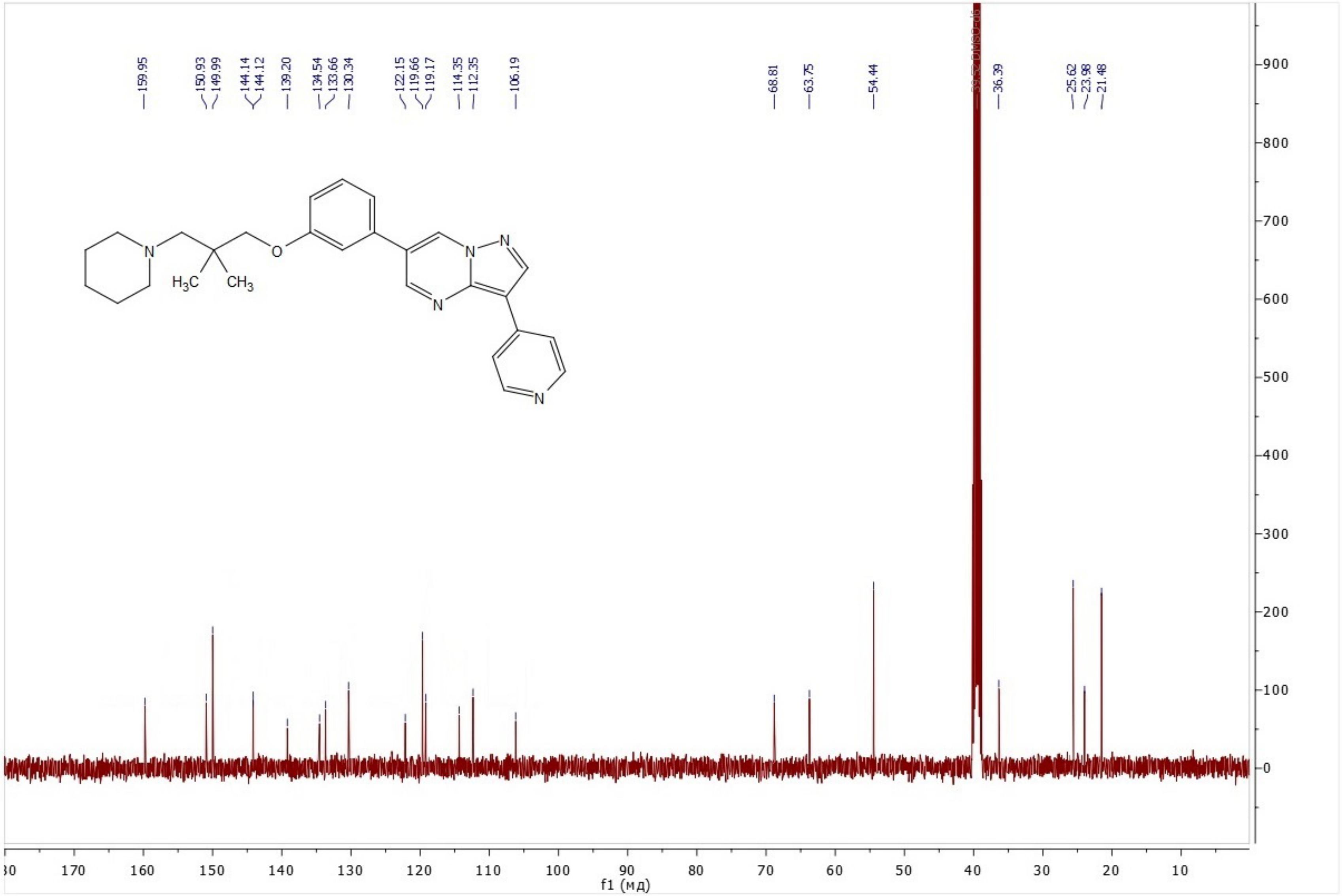

Figure S35. ^{1}H NMR spectrum of **15h**.

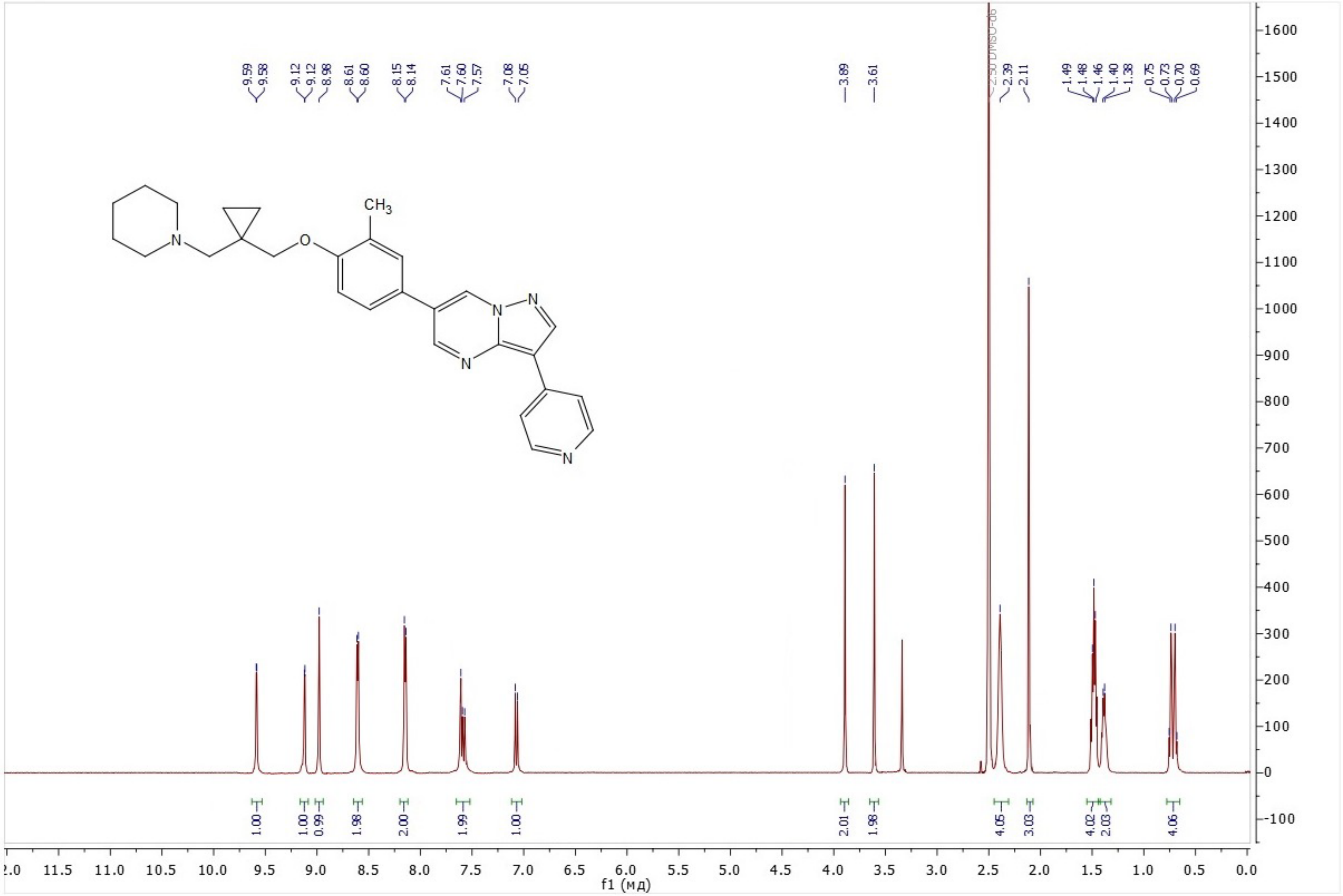

Figure S36. ^{13}C NMR spectrum of **15h**.

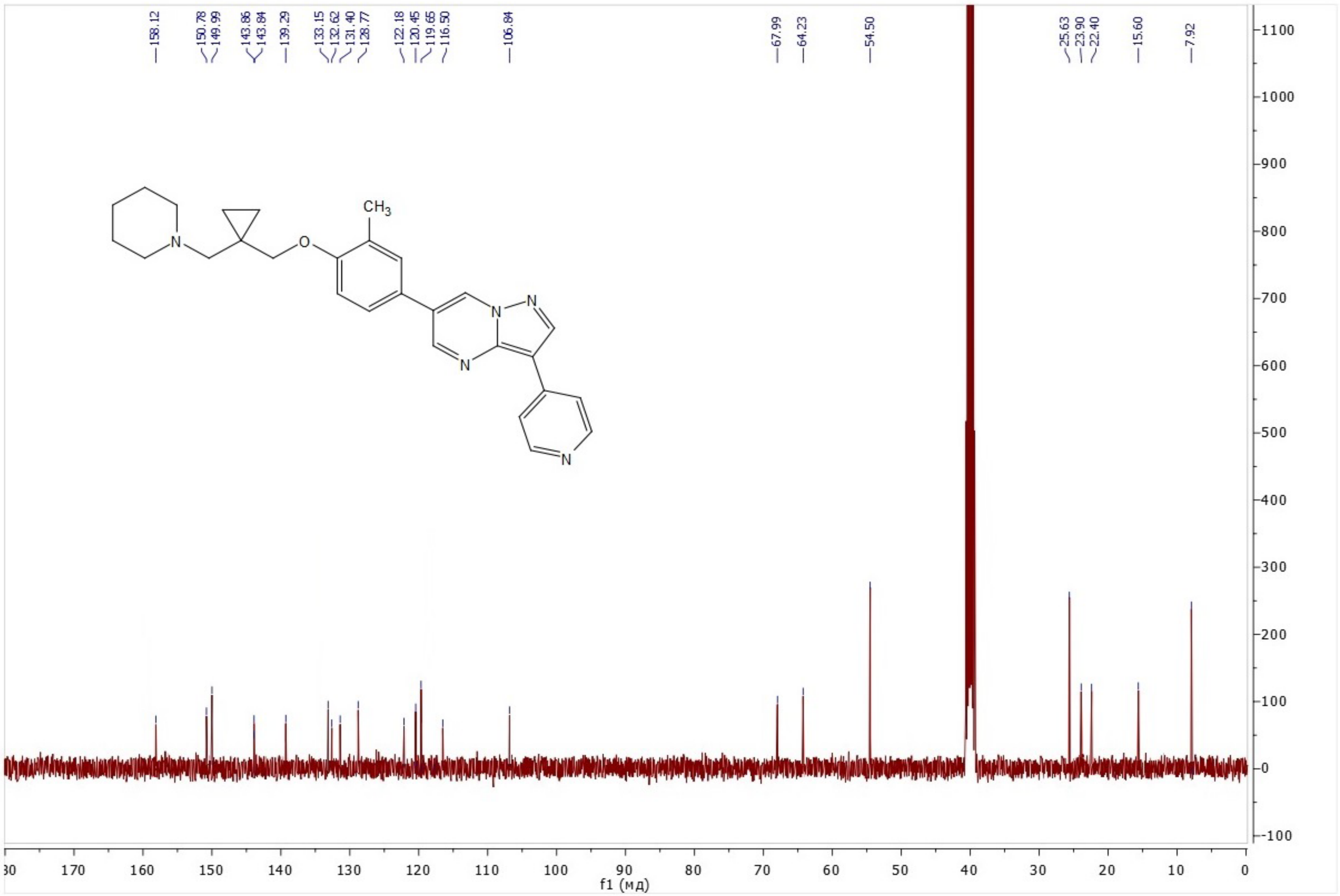

Figure S37. ^{1}H NMR spectrum of **15i**.

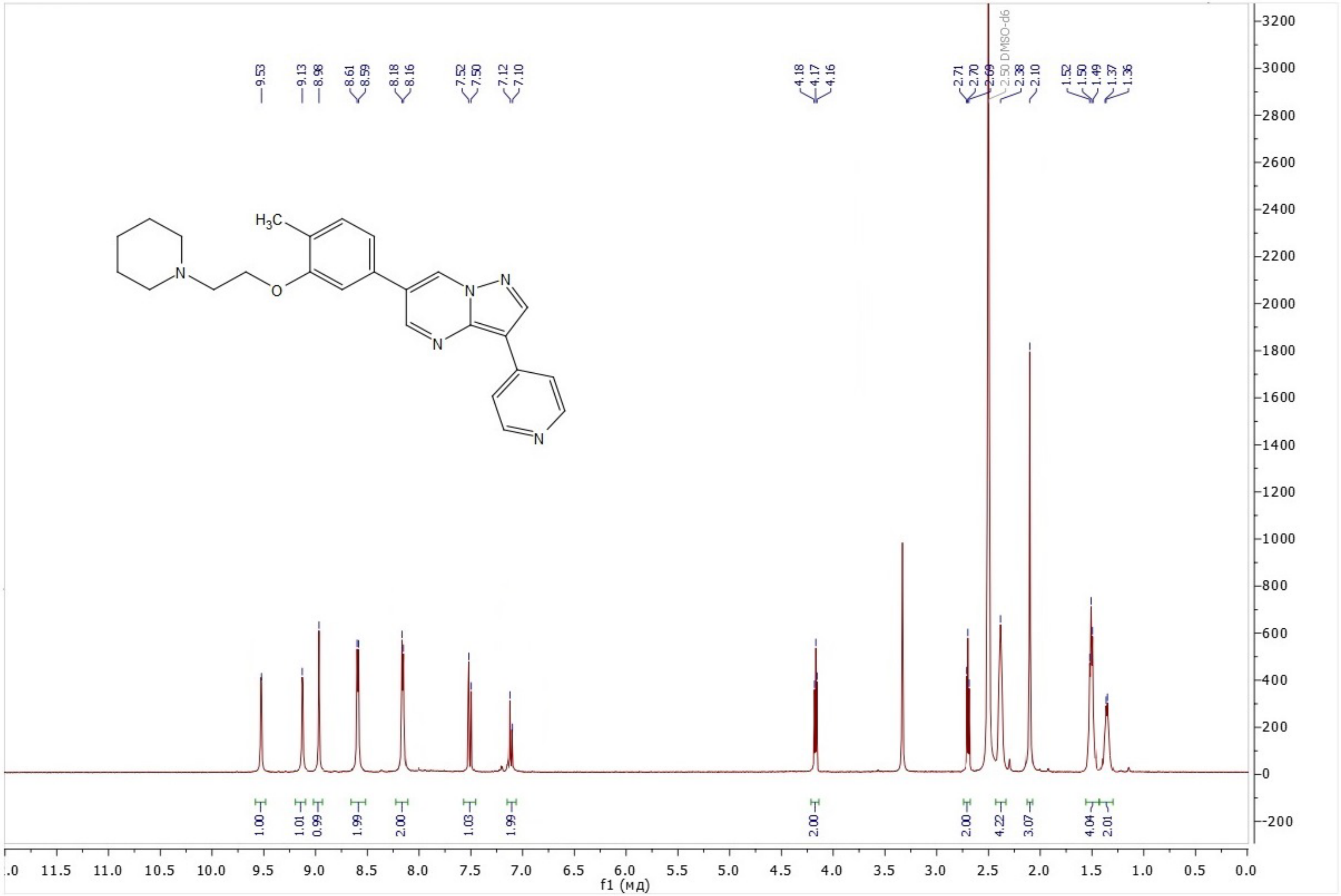

Figure S38. ^{13}C NMR spectrum of **15i**.

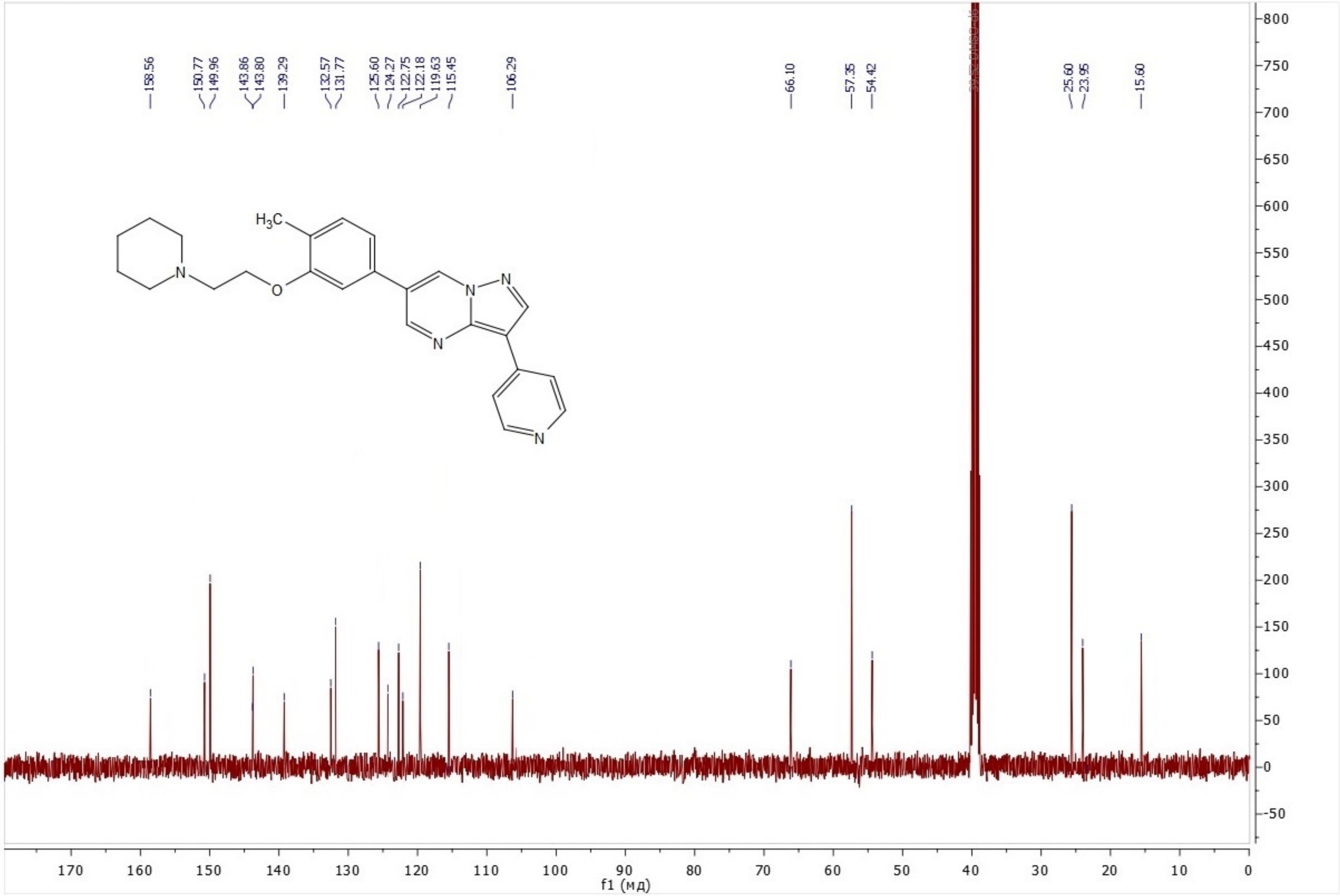

Figure S39. ^{1}H NMR spectrum of **15j**.

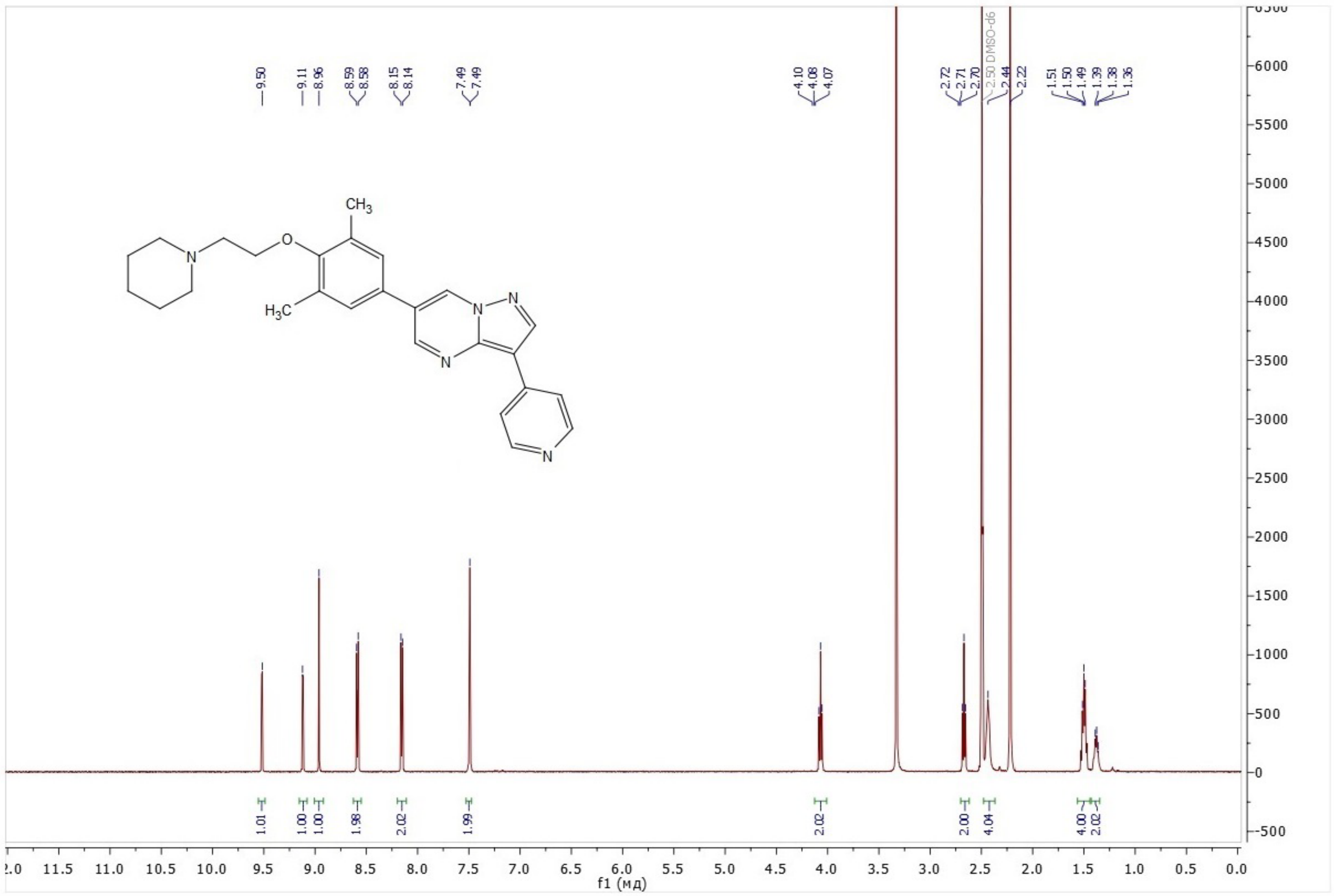

Figure S40. ^{13}C NMR spectrum of **15j**.

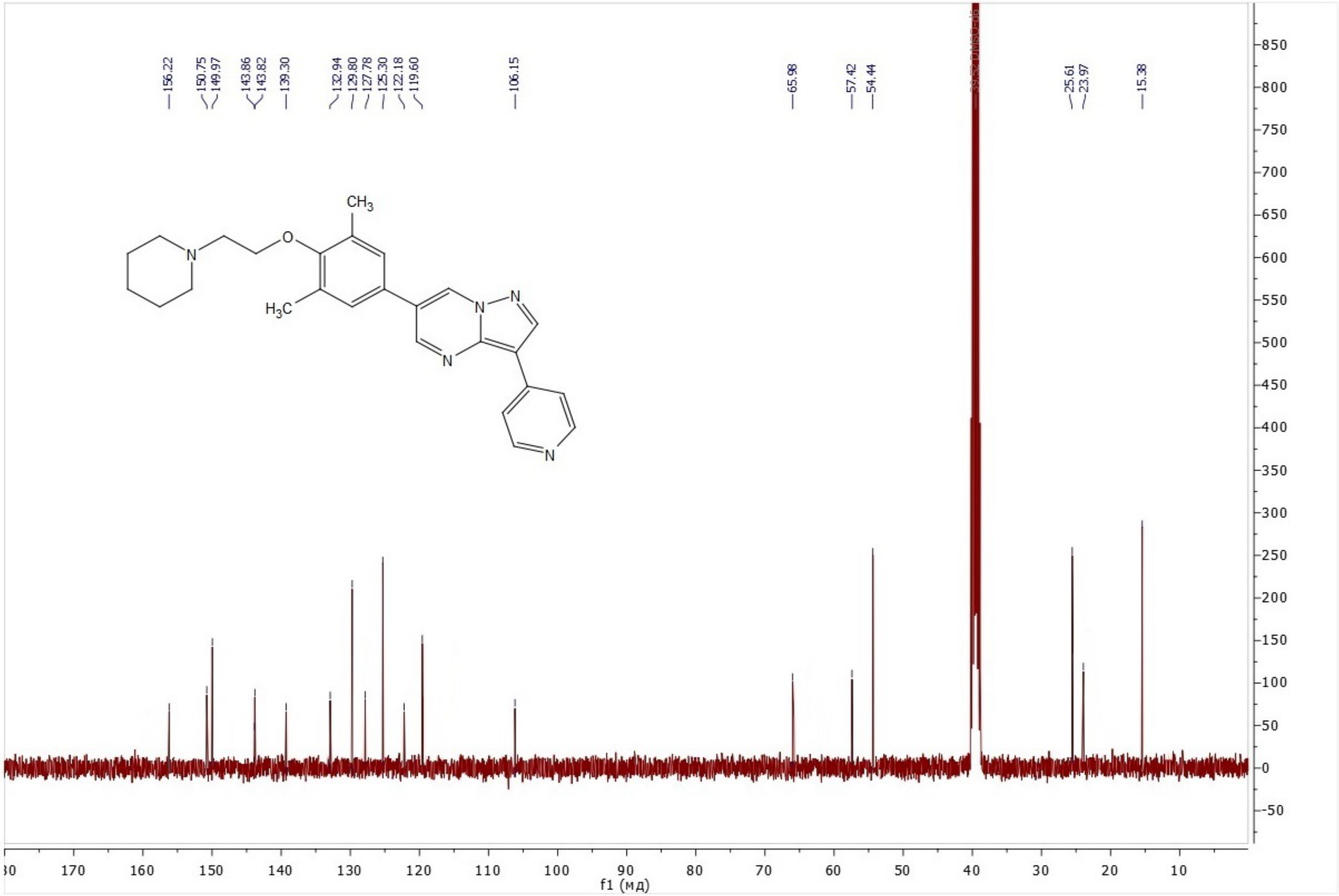

Figure S41. ^{1}H NMR spectrum of **15k**.

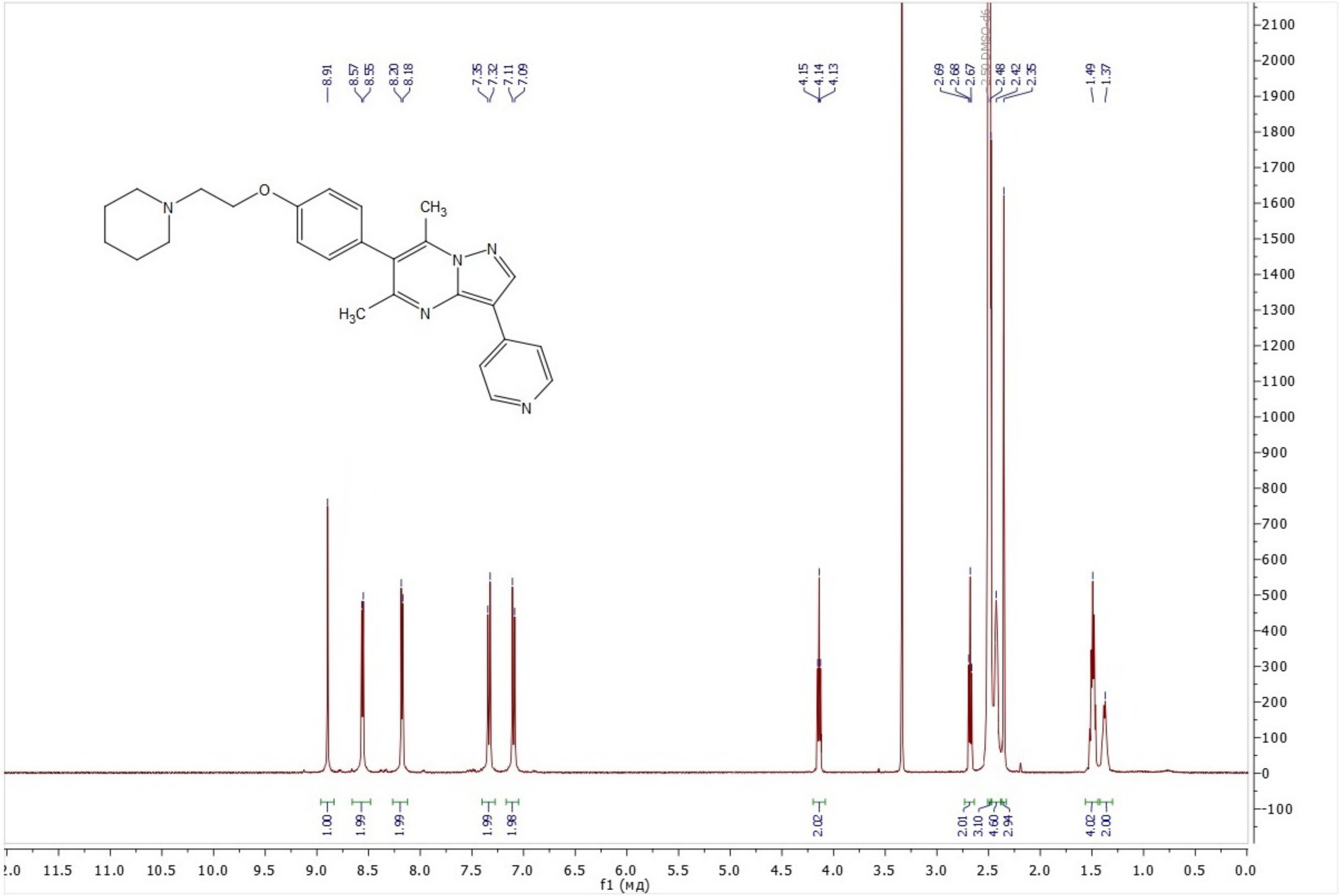

Figure S42. ^{13}C NMR spectrum of **15k**.

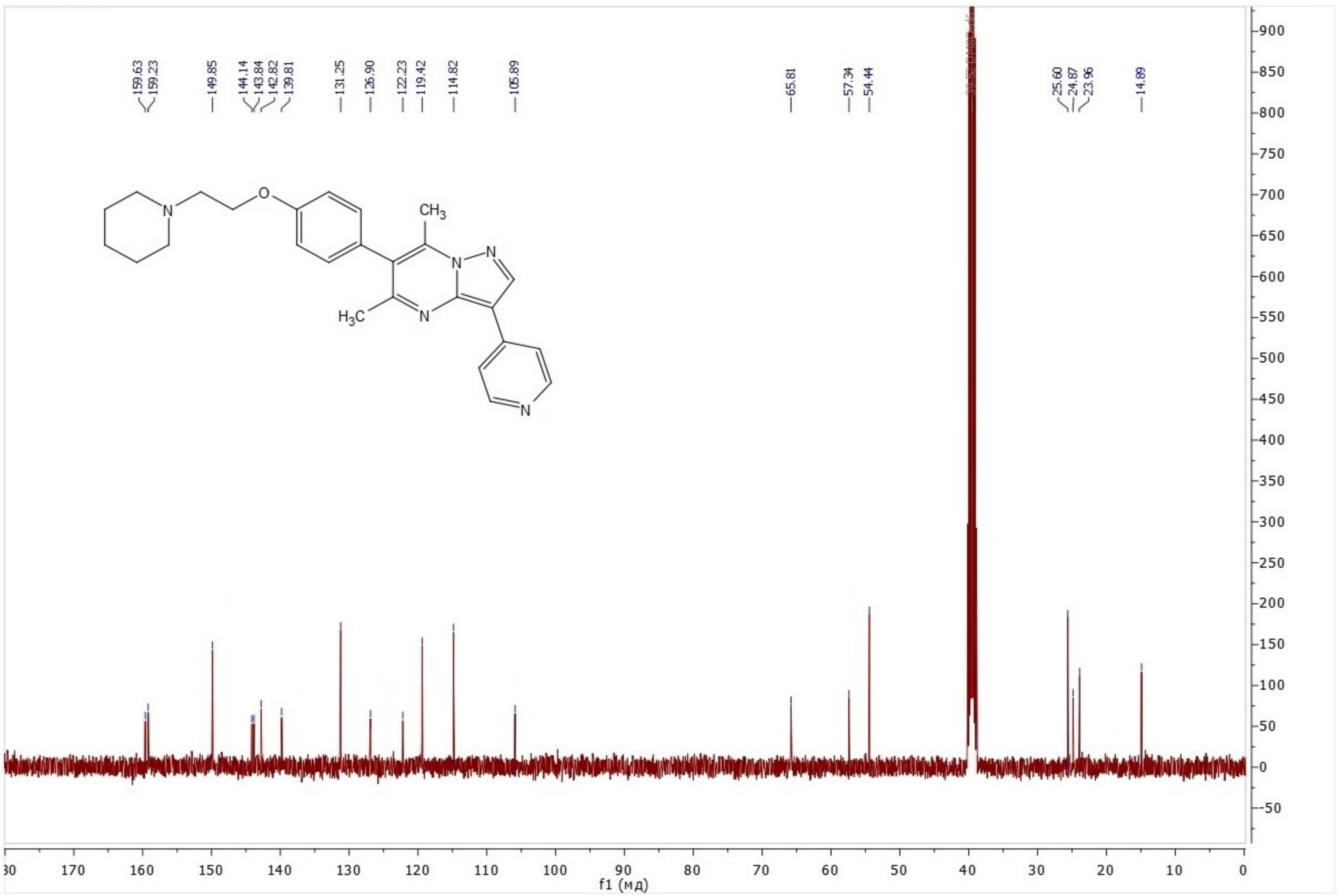

Supplement: Supplementary file 1 [file molecules-30-02258-s001.zip › molecules-3597065-supplementary.pdf]
